# Untargeted metabolomics reveals novel metabolites in *Lotus japonicus* roots during arbuscular mycorrhiza symbiosis

**DOI:** 10.1111/nph.70051

**Published:** 2025-03-17

**Authors:** Josef L. Ranner, Georg Stabl, Andrea Piller, Michael Paries, Sapna Sharma, Tian Zeng, Andrea Spaccasassi, Timo D. Stark, Caroline Gutjahr, Corinna Dawid

**Affiliations:** ^1^ Chair of Food Chemistry and Molecular Sensory Science, TUM School of Life Sciences Technical University of Munich (TUM) Lise‐Meitner‐Str. 34 85354 Freising Germany; ^2^ Plant Genetics, TUM School of Life Sciences Technical University of Munich (TUM) Emil‐Ramann‐Str. 4 85354 Freising Germany; ^3^ Max Planck Institute of Molecular Plant Physiology Am Mühlenberg 1 14476 Potsdam‐Golm Germany; ^4^ TUM CREATE 1 CREATE Way, #10‐02 CREATE Tower Singapore 138602 Singapore; ^5^ Functional Phytometabolomics, TUM School of Life Sciences Technical University of Munich (TUM) Lise‐Meitner‐Str. 34 85354 Freising Germany

**Keywords:** arbuscular mycorrhiza, flavonoids, fungal spore germination, isoflavonoids, *Lotus japonicus*, metabolomics, pterocarpenes, symbiosis

## Abstract

- Arbuscular mycorrhiza (AM) improves mineral nutrient supply, stress tolerance, and growth of host plants through re‐programing of plant physiology.
- We investigated the effect of AM on the root secondary metabolome of the model legume *Lotus japonicus* using untargeted metabolomics. Acetonitrile extracts of AM and control roots were analysed using ultra‐high‐performance liquid chromatography‐electrospray ionization‐ion mobility‐time‐of‐flight‐mass spectrometry (UPLC‐ESI‐IM‐ToF‐MS). We characterized AM‐regulated metabolites using co‐chromatography with authentic standards or isolation and structure identification from *L. japonicus* roots using preparative high‐performance liquid chromatography and nuclear magnetic resonance spectroscopy.
- Arbuscular mycorrhiza triggered major changes in the root metabolome, with most features representing unknown compounds. We identified three novel polyphenols: 5,7‐dihydroxy‐4′‐methoxycoumaronochromone (lotuschromone), 4‐hydroxy‐2‐(2′‐hydroxy‐4′‐methoxyphenyl)‐6‐methoxybenzofuran‐3‐carbaldehyde (lotusaldehyde), and 7‐hydroxy‐3,9‐dimethoxypterocarp‐6a‐ene (lotuscarpene). Further AM‐enhanced secondary metabolites included the previously known lupinalbin A and B, ayamenin D, biochanin A, vestitol, acacetin, coumestrol, and betulinic acid. Lupinalbin A, biochanin A, ayamenin D, liquiritigenin, isoliquiritigenin, lotuscarpene, medicarpin, daidzein, genistein, and 2′‐hydroxygenistein inhibited *Rhizophagus irregularis* spore germination upon direct application.
- Our results show that AM enhances the production of polyphenols in *L. japonicus* roots and highlights a treasure trove of numerous unknown plant secondary metabolites awaiting structural identification and functional characterization.

## Introduction

Arbuscular mycorrhiza (AM), a symbiosis between *c*. 80% of land plant species and fungi of the Glomeromycotina (Spatafora *et al*., 2016), increases mineral nutrient uptake and stress tolerance of plants (Smith & Smith, 2011; Zhang *et al*., 2023; Zou *et al*., 2023), improving overall fitness (Liu *et al*., 2007), boosting photosynthetic rates (Zhu *et al*., 2012), growth, and yield (Ramírez‐Flores *et al*., 2020; Di Tomassi *et al*., 2021; Igiehon *et al*., 2021; Sheteiwy *et al*., 2021).

The fungi collect mineral nutrients from the soil via their extraradical mycelium and release them to the plant inside the root. The plant, in return, nourishes the fungi with photoassimilates, mainly hexoses and lipids (Keymer & Gutjahr, 2018; Wipf *et al*., 2019). This nutrient exchange requires the formation of intracellular, highly branched fungal structures called arbuscules, which are surrounded by a plant membrane called the peri‐arbuscular membrane, across which nutrient exchange takes place (Gutjahr & Parniske, 2013). The environment affects plant development and physiology, and thus considerably influences the state of the symbiosis. Accordingly, the host plant dynamically regulates the formation of intracellular fungal structures such as hyphae and arbuscules and the extent of root colonization to keep the symbiotic advantages at an optimum (Koide & Schreiner, 1992; Gutjahr & Parniske, 2017).

Symbiosis is initiated through an interchange of molecular signals between plant and fungus. Plants grown under phosphate or nitrogen limitation release strigolactones, which activate fungal germination and hyphal branching. In turn, the fungi release chito‐oligosaccharides and lipochito‐oligosaccharides, which activate LysM receptor‐like kinases on the plant side (summarized in Delaux & Gutjahr, 2024). This triggers a symbiotic signalling cascade, including ion channels in the nuclear membrane evoking nuclear calcium oscillations, which are thought to be interpreted by a Ca/calmodulin‐dependent protein kinase (CCamK, Charpentier *et al*., 2016; Miller *et al*., 2013). Activated CCaMK binds and phosphorylates the protein CYCLOPS, a transcription factor that regulates the expression of crucial symbiosis genes (Singh *et al*., 2014). Additionally, CYCLOPS forms a complex with DELLA proteins to activate the transcription factor REQUIRED FOR ARBUSCULAR MYCORRHIZATION 1 (RAM1; Pimprikar *et al*., 2016). The GIBBERELLIC‐ACID INSENSITIVE (GAI), REPRESSOR of GAI (RGA) and SCARECROW (SCR) (GRAS; Pysh *et al*., 1999)) transcription factor RAM1 is involved in activating *RAM2* (Gobbato *et al*., 2012; Park *et al*., 2015; Pimprikar *et al*., 2016), which encodes a glycerol‐3‐phosphate acyltransferase 6 (GPAT6) involved in providing lipids for transfer to the fungus in arbuscule‐containing cells (summarized in Keymer & Gutjahr, 2018). Mutants in *ccamk* block colonization at the hyphopodium stage (Demchenko *et al*., 2004; Pimprikar *et al*., 2016), whereas *cyclops* mutants allow rare events of root entry but no formation of arbuscules (Yano *et al*., 2008). *ram1* and *ram2* allow arbuscule formation, but the arbuscules remain stunted and underdeveloped (Pimprikar *et al*., 2016; Keymer *et al*., 2017).

Colonization of plant roots by AM fungi is accompanied by drastic changes in the transcriptome (reviewed in Pimprikar & Gutjahr, 2018) including many genes involved in secondary metabolism. In *Medicago truncatula* and some other plant species, metabolomics of symbiotic roots, as well as leaves of symbiotic plants, revealed strong AM‐induced changes in the metabolome, but as usual in untargeted metabolomics, many compounds could not be identified (Schliemann *et al*., 2007; Schweiger & Müller, 2015; Kaur *et al*., 2022). In general, the knowledge on alterations of the secondary metabolome in response to AM symbiosis is rather poor (Ranner *et al*., 2023).

Therefore, we analysed extracts from roots of the model legume *L. japonicus* colonized by the model AM fungus *Rhizophagus irregularis* compared to control roots, using untargeted metabolomics by ultraperformance liquid chromatography‐electrospray ionization‐ion mobility‐time‐of‐flight‐mass spectrometry (UPLC‐ESI‐IM‐ToF‐MS). We identified AM‐enhanced compounds via co‐chromatography with authentic standards or isolation followed by spectroscopic and spectrometric analyses. Multi‐omics correlation analysis with metabolomics and transcriptomics data was used to visualize AM‐induced trends. Finally, we evaluated the activity of identified and highly induced compounds on fungal spore germination. Root colonization by AM fungi induced massive metabolome changes. We found novel compounds induced by AM and provided a basis for the elucidation of further novel compounds, metabolic pathways, and their functions in plants and AM symbiosis.

## Materials and Methods

### General experimental procedure

The following chemicals and solvents were obtained commercially: acetonitrile (MeCN), methanol (Honeywell, Seelze, Germany); acacetin, acetic acid, acetone‐*d*6, acetonitrile‐*d*3, betulinic acid, biochanin A, daidzein, dimethyl sulfoxide (DMSO), DMSO‐*d*6, formic acid, formononetin, isoliquiritigenin, liquiritigenin, methanol‐*d*6, potassium hydroxide (Sigma Aldrich, Steinheim, Germany); ethyl acetate, *n*‐pentane (VWR International, Rosny‐sous‐Bois, France); leucine‐enkephalin, Major Mix IMS/ToF Calibration Kit (Waters, Milford, MA, USA); liquid nitrogen (Westfalen, Münster, Germany); coumestrol (Enzo Clinical Labs, Farmingdale, NY, USA); lupinalbin A (Clinivex, Toronto, Canada); 2′‐hydroxyformononetin, isosativan, lupinalbin B, medicarpin, sativan (Biosynth Carbosynth, Compton, UK); genistein, vestitol (Biorbyt, Cambridge, UK), vestitone (TRC, Toronto, Canada), catechin (Extrasynthese, Geny, France), 2′‐hydroxygenistein (Biocrick, Sichuan, China).

Eluants for liquid chromatography‐mass spectrometry (LC‐MS) analysis were of LC‐MS grade. Ethyl acetate and *n*‐pentane were distilled before use. Water used for extraction and chromatography was purified using a Milli‐Q Reference A+ combined with an Elix Essential 3 water system equipped with a 0.2 μm PES high‐flux capsule filter (Millipore, Schwalbach, Germany).

One‐ and two‐dimensional nuclear magnetic resonance (1D/2D NMR) spectroscopy experiments ^1^H, ^1^H‐^1^H‐correlation spectroscopy (^1^H‐^1^H‐COSY), heteronuclear single quantum coherence (HSQC), heteronuclear multiple bond correlation (HMBC), and ^13^C were performed on a 600 MHz Avance NEO spectrometer (Bruker, Rheinstetten, Germany) equipped with a Triple Resonance TCI Cryo probe head (Bruker) or a 950 MHz Avance III spectrometer with a cryo‐TCI probe head (Bruker). Small 3 × 100 mm NMR tubes were used (Hilgenberg, Malsfeld, Germany). Data were acquired using TopSpin (v.3.6.0, Bruker) and analysed using MestReNova (v.12.0.2, Mestrelab Research, Santiago de Compostela, Spain). Chemical shifts were expressed as parts per million (ppm) relative to the solvent signal of acetonitrile‐*d*_3_, methanol*‐d*_4_, or acetone‐*d*_6_. Mass spectral analyses for untargeted measurement and for structure elucidation were conducted on a Waters Vion IMS QToF mass spectrometer (Waters, Manchester, UK) linked to an Acquity UPLC I‐class system consisting of a binary solvent manager, a sample manager, and a column oven (Waters, Milford, MA, USA).

For medium pressure liquid chromatography (MPLC), a Büchi Sepacore system (Flawil, Switzerland) was used, which combines a C‐620 control unit, a C‐605 binary pump module, a C‐660 fraction collector, and a C‐635 UV photometer. The system was equipped with a polypropylene cartridge (40 × 150 mm, Büchi) filled with LiChroprep RP18 (25–40 μm) material (Merck, Darmstadt, Germany). Sepacore Control Chromatography software (v.1.2, Büchi) was used for instrument control and data acquisition. Preparative and semi‐preparative high‐performance liquid chromatography (HPLC) fractionation was performed on a Jasco HPLC system consisting of PU‐2087 Plus binary pumps, a DG‐2080‐53 degasser, a UV 2070 Plus detector (Jasco, Groß‐Umstadt, Germany), and a Rh 7725i‐type Rheodyne injection valve (Rheodyne, Bensheim, Germany). For instrument control and data analysis, the Chrompass Chromatography software (v.1.9.302.1124, Jasco) was used. Before preparative chromatography, all samples were sonicated (15 min) and membrane‐filtered (0.45 μm).

All subfractions were freed from solvent (vacuum, 40°C), lyophilized, and kept at −20°C until further use. For rotary evaporation of organic solvents, a Büchi Rotovapor R‐210 with a Vacuum Controller V‐850, a Heating Bath B‐491, a Vacuum Pump V‐700, and a Recirculating Chiller B‐740 (Büchi, Flawil, Switzerland) was used. Samples, extracts, and fractions were lyophilized using a Delta 1‐24 LSC (Christ, Osterode, Germany).

### Plant material and quantification of root colonization

*Lotus japonicus* (Regel) K. Larsen wild‐type (WT, ecotype Gifu B‐129) and mutants *ccamk‐3*, *ccamk‐13*, *cyclops‐3*, *cyclops‐4*, *ram1‐3*, *ram1‐4*, *ram2‐1*, and *ram2‐2* (Yano *et al*., 2008; Perry *et al*., 2009; Pimprikar *et al*., 2016; Keymer *et al*., 2017) were inoculated with *R. irregularis* spores and cultivated as follows (Torabi *et al*., 2021).

Seeds were scarified with sandpaper for 5 min and surface sterilized using 2% NaClO and 0.1% sodium dodecyl sulphate (Colgate‐Palmolive, New York City, NY, USA) in water for 8 min. The seeds were washed three times with sterile water and incubated in sterile water for 45 min. Imbibed seeds were germinated in the dark on 0.8% agar (Duchefa, Haarlem, the Netherlands) at 24°C for 3 d and then subjected to a 16 : 8 h, light : dark cycle at 24°C for 11 d. Wild‐type seeds for RNA sequencing (RNA‐seq) were germinated on B5 medium (Duchefa). Seedlings were grown in pots filled with autoclaved quartz sand (0.7–1.2 mm; Casafino, Munich, Germany) and a 40 ml of half‐strength Hoagland medium containing 5 mm Ca(NO_3_)_2_, 5 mm KNO_3_, 1 mm MgSO_4_, 50 μm NaFeEDTA (ethylenediaminetetraacetic acid), 20 μm KH_2_PO_4_, 10 μm H_3_BO_3_, 0.2 μm Na_2_MoO_4_, 1 μm ZnSO_4_, 2 μm MnCl_2_, 0.5 μm CuSO_4_, 0.2 μm CoCl_2_, 12.5 μm HCl, and 500 μm 2‐(*N*‐morpholino)ethanesulfonic acid (MES) at pH 6.1 (Hoagland & Arnon, 1938; Torabi *et al*., 2021). For colonization with *R. irregularis*, roots were inoculated with 500 spores (Agronutrition Toulouse, France RI DAOM197198, quality C) per plant. The plants were fertilized once per week with 30 ml half‐strength Hoagland solution per pot and watered twice per week with 30 ml of sterilized water. The plants for metabolomics analysis were grown in two sets, one for 7 wk and one for 10 wk, and the plants for RNA‐seq were grown for 7 wk in a chamber in a long‐day photoperiod (16 h : 8 h, 24°C : 20°C, light : dark) and 60% relative humidity. The plant roots were harvested 7 wk post inoculation (wpi) and 10 wpi, respectively, frozen in liquid nitrogen (77 K), and stored at −80°C. Plants from one pot were pooled to represent one biological replicate. Plants for RNA‐seq were chopped into 1–2 cm pieces, mixed thoroughly, and used partly for microscopy and partly for RNA isolation.

After harvesting, two to three root systems per pot were boiled in 10% KOH for 15 min, washed three times with water and once with 10% acetic acid. The roots were stained using a solution of 5% black ink in 5% acetic acid and destained using 5% acetic acid, as reported previously (Vierheilig *et al*., 1998; Torabi *et al*., 2021). Root pieces of 1 cm length were analysed to quantify root colonization using a modified gridline intersect method (McGonigle *et al*., 1990). The root segments were monitored at 10‐fold magnification under a light microscope, Leica, type 020‐18 500 DM/LS (Leica, Germany).

To prepare RNA, the Spectrum Plant Total RNA‐Kit (Sigma Aldrich) was used according to the manufacturer's instructions. mRNA was enriched using NEBNext Poly(A) mRNA Magnetic Isolation Module (New England Biolabs, Ipswich, MA, USA) followed by library preparations using NEBNext Ultra RNA Library Prep Kit (New England Biolabs). Sequencing was performed on a NovaSeq 6000 S4 platform (Illumina, San Diego, CA, USA) at Beijing Genomics Institute (BGI). After sequencing, raw reads were filtered by BGI using SOAPnuke (Chen *et al*., 2018) to produce clean reads. Clean reads were mapped to the *L. japonicus* Gifu genome v.1.2 (Kamal *et al*., 2020) using Star aligner (Dobin *et al*., 2013). Read count matrices were obtained by counting the number of uniquely mapped reads of each gene using featureCounts (Liao *et al*., 2014). An updated *L. japonicus* genome annotation file (v.1.3) from LotusBase (Mun *et al*., 2016) was used for featureCounts. Normalization and differential gene expression analyses were performed using DESeq2 (Love *et al*., 2014) in R loading the read count matrices as input. Reads were deposited in NCBI with accession no. PRJNA1086535.

### Extraction

*Lotus japonicus* roots used for the untargeted metabolome analysis were lyophilized, and samples (a pool of two to three roots per biological replicate) were extracted twice with MeCN (2 × 2.75 ml) using a Precellys Evolution tissue homogenizer (Bertin Technologies, Montigny‐le‐Bretonneux, France) shaking 6000 times per minute for three 25 s cycles with 20 s breaks. The homogenizer tubes were centrifuged at 3202 rcf (4000 rpm) for 6.5 min using an Eppendorf Centrifuge 5810 R with an A‐4‐62 rotor (Eppendorf, Hamburg, Germany). The supernatant was subjected to membrane filtration (RC 0.45 μm, Sartorius, Göttingen, Germany) and diluted with MeCN to a concentration of 3.5 mg extraction material ml^−1^.

For the isolation of marker compounds, nonmycorrhizal *L. japonicus* plants were grown to maturity (5–6 months) in a glasshouse, and roots of these plants (1050 g) were harvested for compound extraction. Further, mycorrhizal *L. japonicus* plants were grown in a phytochamber (equally to the plants used for the untargeted analysis, Torabi *et al*., 2021), and colonized roots (450 g) were harvested at 7 wpi and used for compound isolation. After harvesting, the roots were dissected, washed with tap water, frozen in liquid nitrogen (77 K), and ground at 4000 rpm for 2 min using a Grindomix GM 300 knife mill (Retsch, Haan, Germany). The powder was extracted three times with twice the amount of solvent, stirred for 10 min, and purified using a borosilicate glass filter. Another filtration step using a folded filter was performed to partition colloids. The solvents used were distilled *n*‐pentane (yield: < 0.1%), distilled ethyl acetate (y: 0.4%), methanol (y: 2.9%), methanol/water (70/30, v/v, y: 0.7%), and water (y: 0.4%) in this order. The filtrates were concentrated until solvent free using rotary evaporation and lyophilized.

### Compound isolation

Medium pressure liquid chromatography and solid‐phase extraction (SPE) fractions were fractionated by preparative HPLC (Supporting Information Table S1; Methods S1) to isolate lupinalbin A (**4**), 5,7‐dihydroxy‐4′‐methoxycoumaronochromone (**13**), ayamenin D (**14**), 4‐hydroxy‐2‐(2′‐hydroxy‐4′‐methoxyphenyl)‐6‐methoxybenzofuran‐3‐carbaldehyde (**15**), 7‐hydroxy‐3,9‐dimethoxypterocarp‐6a‐ene (**16**), 4,6‐dihydroxy‐2‐(2′‐hydroxy‐4′‐methoxyphenyl)‐3‐(methoxymethyl)benzofuran (**25**), 4‐hydroxy‐2‐(2′‐hydroxy‐4′‐methoxyphenyl)‐6‐methoxy‐3‐(methoxymethyl)benzofuran (**26**), and an unknown dimeric polyphenol artefact (**29**).

### Pterocarp‐6a‐ene alcoholysis

An aliquot (100 μl) of a MeCN extract (3.5 mg ml^−1^) of well‐colonized (67%) *L. japonicus* roots was lyophilized and redissolved in MeOH, ethanol (EtOH), and MeCN (100 μl), respectively. The solutions were kept at room temperature under UV light (254 nm) using a Chromato‐Vue C‐75 UV Darkroom Cabinet (UVP LLC, Upland, CA, USA). After 0, 24, 48, and 72 h, the samples were analysed by UPLC‐ToF‐MS to determine normalized peak responses of pterocarp‐6a‐enes and their potential artefacts.

### UPLC‐ESI‐IM‐ToF‐MS

Aliquots (3 μl) of the extracts (3.5 mg ml^−1^) were analysed in three technical replicates by UPLC‐ESI‐IM‐ToF‐MS on a Waters Vion IMS‐QToF mass spectrometer (Waters, Manchester, UK) coupled to an Acquity UPLC I‐class system (Waters, Milford, MA, USA) equipped with a 2 × 150 mm, 1.7 μm, Acquity BEH C18 column (Waters, Milford, MA, USA), and with an upstream VanGuard UPLC BEH C18 pre‐column (2.1 × 5 mm, 1.7 μm, Waters). The sample manager was set to an autosampler temperature of 10°C and a column oven temperature of 45°C. For chromatography, 0.1% formic acid in water (v/v, solvent A) and MeCN (solvent B) were used and the following gradient was applied at a flow rate of 0.4 ml min^−1^: 0% B to 100% B within 9 min, kept at 100% B for 4 min to 0% B within 1 min, and kept at 100% B for 1 min. Scan time for the MS^e^ method was set to 0.2 s. Data acquisition was performed using negative ESI in sensitivity mode with the following source parameters: capillary voltage: −1.50 kV, sampling cone voltage: 40 V, source offset: 80 V, source temperature: 150°C, desolvation temperature: 450°C, cone gas: 50 l h^−1^, desolvation gas: 850 l h^−1^, reference capillary voltage: 2.00 kV. For MS^e^ acquisition, low energy was set to 6.00 eV and high‐energy Ramp was set to −20.00 eV to −60.00 eV. System control, data acquisition, and processing were carried out using UNIFI Scientific Information System (v.1.9.4.053; Waters). Automatic Lock‐Mass correction was performed in an interval of 0.5 min using leucine‐enkephalin (Tyr‐Gly‐Gly‐Phe‐Leu, mass‐to‐charge ratio (*m/z*) 554.2620 [M−H]^−^) in a solution (100 pg ml^−1^) of MeCN/0.1% formic acid in water (50/50, v/v). Mass spectrometry calibration in a range of 50–1500 Da was performed using the Waters MajorMix solution consisting of acetaminophen (10.0 μg ml^−1^), caffeine (1.5 μg ml^−1^), sulfaguanidine (5.0 μg ml^−1^), sulfadimethoxine (1.0 μg ml^−1^), Val‐Tyr‐Val (2.5 μg ml^−1^), verapamil (0.2 μg ml^−1^), terfenadine (0.2 μg ml^−1^), leucine‐enkephalin (2.5 μg ml^−1^), and reserpine (0.6 μg ml^−1^) in MeCN/0.1% formic acid in water (5.7/94.3, v/v).

Raw data from UPLC‐ESI‐IM‐ToF‐MS analysis were processed with Progenesis QI (v.2.4; Waters, Milford, MA, USA) and the Progenesis QI workflow was applied with the different steps being import data, review alignment, experiment design setup, peak picking, review deconvolution, review compounds, and compound statistics. Quality control samples consisting of an equal amount (37 μl) of every analysed sample pooled together served as reference runs for automatic normalization and peak alignment to compensate for retention time (RT) drifts between runs. Peak picking parameters were set to all runs, limits automatic, sensitivity 3, and no RT limits. Features used for principal component analysis (PCA) were filtered by means of ANOVA *P*‐value ≤ 0.05 and a fold change of ≥ 2. The processed data were further analysed by partial least squares discriminant analysis with pareto scaling using EZinfo (3.0.3.0, Waters). Orthogonal partial least squares discriminant analysis (OPLS‐DA), represented as *S* plots, was used to determine group differences. Sum formula calculations for potential marker features were performed using Progenesis QI and the following parameters: H (0–300), C (0–120), N (0–10), O (0–50), precursor tolerance 5 ppm and isotope similarity 95%.

A database containing structural MOL file information for 320 metabolites described in *L. japonicus* (Ranner *et al*., 2023) was created with Progenesis SDF Studio (v.1.0; Waters) and applied to pre‐selected *S*‐plot markers, facilitating *m/z*‐based matching of precursor and fragment ions with *in silico* fragments, and thus tentative compound identification (tolerance 5 ppm). Normalized untargeted UPLC‐ESI‐IM‐ToF‐MS features were subjected to statistical analysis and data visualization in the R language environment for statistical computing (v.4.2.1; R Core Team, 2013). Data were centred and scaled using the base R scale function. Heatmaps were created using the complex heatmap R package (Gu *et al*., 2016).

Compounds were identified by comparing their RT, rotationally averaged collision cross‐sectional areas (CCS), accurate *m*/*z*, and mass fragments to those of purchased or isolated standard compounds. Identities were verified by co‐chromatography of the authentic standard with a well‐colonized *L. japonicus* root extract sample. Analysis of the correlation of metabolomics and transcriptomics data is described in Methods S2.

### Spore germination assay

*Rhizophagus irregularis* spores (Agronutrition Toulouse, France, RI DAOM197198, quality A) were washed two times with sterile _dd_H_2_O and plated on sterile 24 well plates (Sigma Aldrich, CLS3526‐50EA) with modified medium for the asymbiotic culture experiments of *R. clarus* HR1 (table S1 in Tanaka *et al*., 2022; instead of Phytagel, we used Gelrite 3 g l^−1^; DC Chemicals, G1101.1000). Spore germination was assessed in triplicates. Polyphenols were dissolved in DMSO to receive stock solutions of 10 mm. Aliquots of the stock solutions were added to the medium while pouring the plates to a final concentration of 10 μm. Dimethyl sulfoxide blank and medium blank samples were treated in the same way for each plate. The spores were photographed after plating (*t*_0_) and then placed in the dark at 28°C for 7 d. After 7 d, the spores were photographed again (*t*_7_), and the number of germinated and nongerminated spores was counted.

## Results

### Untargeted metabolomics exposes significant effects of AM on the secondary metabolome of *L. japonicus* roots

We used untargeted metabolomics to investigate metabolome changes upon colonization of *L. japonicus* (Gifu) roots with the AM fungus *R. irregularis*. To understand to which extent the progression of AM colonization shapes the AM‐responsive metabolome, we included mutants stalled at different steps of colonization, namely two allelic mutants of *ccamk* and *cyclops* (*ccamk‐3*, *ccamk‐13*, *cyclops‐3*, *cyclops‐4*; Yano *et al*., 2008; Perry *et al*., 2009), which are impaired in root entry and arbuscule formation, respectively, and of *ram1* and *ram2*, both impaired in arbuscule branching (*ram1‐3*, *ram1‐4*, *ram2‐1*, and *ram2‐2*; Pimprikar *et al*., 2016; Keymer *et al*., 2017). Furthermore, we used two independent experiments harvested at two different time points, 7 wpi and 10 wpi.

Ultraperformance liquid chromatography‐electrospray ionization‐ion mobility‐time‐of‐flight‐mass spectrometry results were analysed using the Progenesis QI workflow, and mass spectral features were filtered on the basis of ANOVA *P*‐value ≤ 0.05 and a fold change of ≥ 2. The cutoff reduced features from a total of 11 841 across all genotypes to 1782 for roots harvested at 7 wpi and from a total of 3919 across all genotypes to 3649 for roots harvested at 10 wpi (Table 1).

**Table 1:** Mass spectrometry (MS) features detected by metabolomics analysis of *Lotus japonicus* roots harvested at 7 and 10 wk post inoculation (wpi) before (total) and after applying significance thresholds (ANOVA; *P*‐value ≤ 0.05 and fold change ≥ 2) and reduced numbers of features (*n*) included in orthogonal partial least squares discriminant analysis (OPLS‐DA).

Genotype		Roots harvested at 7 wpi			Roots harvested at 10 wpi		
		MS features total^a^	MS features after cut‐off^a^		MS features total^a^	MS features after cut‐off^a^	
		11, 841	1782		3919	3649	
		*n*	*P*[1] > 0	*P*[1] < 0	*n*	*P*[1] > 0	*P*[1] < 0
Gifu wild‐type	Total^b^	1781	1132 (9.6%)	649 (5.5%)	3426	1690 (43.1%)	1711 (43.7%)
	Markers		9	3		12	0
*ccamk‐3*	Total^b^	1726	1056 (8.9%)	659 (5.6%)	3104	1840 (47.0%)	1255 (32.0%)
	Markers		20	0		4	1
*ccamk‐13*	Total^b^	1737	1022 (8.6%)	709 (6.0%)	3214	2120 (54.1%)	1086 (27.7%)
	Markers		19	2		9	0
*cyclops‐3*	Total^b^	1705	339 (2.9%)	1254 (10.6%)	3198	1848 (47.2%)	1337 (34.1%)
	Markers		1	7		9	6
*cyclops‐4*	Total^b^	1705	677 (5.7%)	1023 (8.6%)	Not evaluated^c^		
	Markers		9	3			
*ram1‐3*	Total^b^	1733	1195 (10.1%)	529 (4.5%)	2839	1160 (29.6%)	1666 (42.5%)
	Markers		11	4		7	6
*ram1‐4*	Total^b^	1750	799 (6.7%)	946 (8.0%)	3172	1837 (46.9%)	1323 (33.8%)
	Markers		3	6		5	5
*ram2‐1*	Total^b^	1640	1007 (8.5%)	627 (5.3%)	3162	1940 (49.5%)	1213 (31.0%)
	Markers		13	3		4	5
*ram2‐2*	Total^b^	1696	892 (7.5%)	800 (6.8%)	3292	1778 (45.4%)	1502 (38.3%)
	Markers		10	3		4	5

Features with a positive loading value (*P*[1] > 0) are positively associated with arbuscular mycorrhizal (AM) roots, whereas features with a negative loading value (*P*[1] < 0) are positively associated with control roots. Their absolute and relative (in brackets) numbers are given. Reliability thresholds of OPLS‐DA loading value |*P*[1]| > 0.1 and correlation value |*P*(corr)[1]| > 0.5 were selected to obtain potential marker feature candidates.

^a^ Across all genotypes.

^b^ Relative number of features in relation to the total number of MS features.

^c^ No evaluation, as only one biological replicate was available per condition.

The features that passed the filtering were subjected to PCA, including control and AM samples of all genotypes. When all samples were combined in one PCA, there was no clustering between mycorrhizal and nonmycorrhizal roots at 7 wpi (Fig. S1a), whereas a slight separation was observed in the PC1 dimension for roots harvested at 10 wpi (Fig. S1b). We also analysed variation for each individual genotype (Figs S1c,d, S2, S3). For roots harvested at 7 wpi, the WT samples showed the clearest separation among all genotypes between AM and control roots (Figs S1c, S2), indicating that the WT responded most strongly with metabolome changes to AM. Wild‐type control and AM roots were separated along PC1, and the AM samples showed an additional separation along PC1 and PC2, reflecting their different levels of colonization ranging from 26% to 67% (refer to colonization data in Table S2). This suggests that the extent of metabolome changes is somewhat correlated with the root colonization level. In roots harvested at 10 wpi, the metabolomes of WT control and AM roots separated along both axes, with some control and less colonized AM roots overlapping in the PC1 dimension (Fig. S1d).

**Fig. 1:**
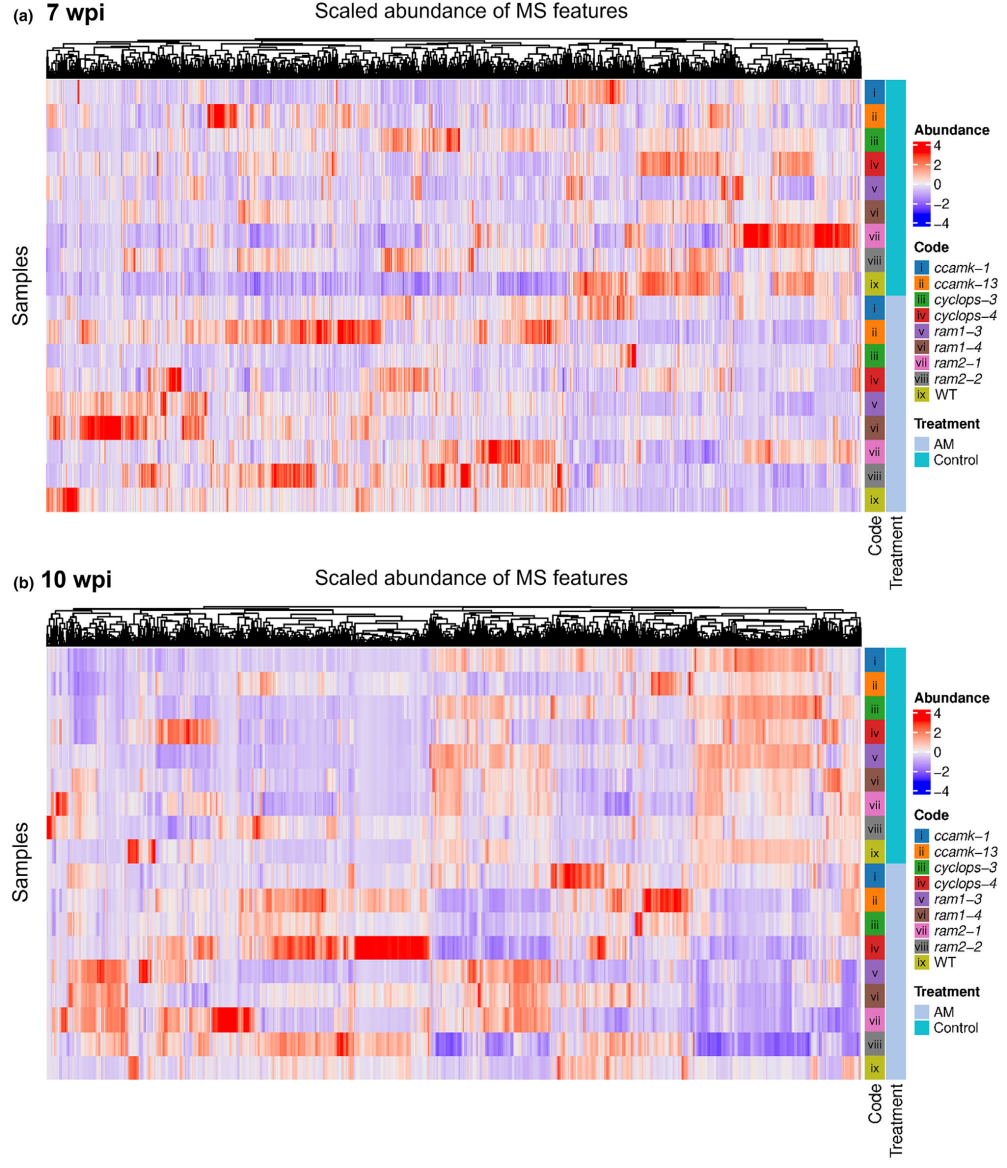
Heatmaps comparing scaled normalized abundance of pre‐filtered mass spectrometry (MS) features (ANOVA *P*‐value ≤ 0.05; fold change ≥ 2) detected by ultraperformance liquid chromatography‐electrospray ionization‐ion mobility‐time‐of‐flight‐mass spectrometry (UPLC‐ESI‐IM‐ToF‐MS) full scan analysis (50–1200 Da, ESI^−^, sensitivity mode) across control and arbuscular mycorrhizal (AM) samples of *Lotus japonicus* wild‐type (WT) and eight mutants (*ccamk‐3*, *ccamk‐13*, *cyclops‐3*, *cyclops‐4*, *ram1‐3*, *ram1‐4*, *ram2‐1*, and *ram2‐2*) harvested at (a) 7 wk post inoculation (wpi) and (b) 10 wpi.

Scaled normalized abundances of the pre‐filtered MS features across AM and control WT and mutant roots revealed clusters of features, which were abundant in control roots and showed reduced levels in AM roots across nearly all genotypes at 7 wpi and all genotypes at 10 wpi (Fig. 1). This indicates that all genotypes reduce biosynthesis of certain compounds in the presence of the fungus, irrespective of whether the genotype can be colonized or not. Additionally, the WT showed several smaller clusters of features with increased abundance in colonized roots, and at 7 wpi, some of these features were also increased in both allelic *ram1* or both *ram2* mutants or mainly in *ram2* mutants at 10 wpi. Interestingly, both allelic *ccamk* mutants, *ram1* and *ram2* mutants, showed AM‐induced increases in a number of features, which were not increased in the WT at 7 wpi, and this effect was observed for all mutants at 10 wpi. It will be interesting to identify these compounds in the future and to understand why they increase in incompatible plant–fungal interactions and not in the compatible and functional AM fungal interaction with the WT. In summary, we observed significant metabolome changes in response to AM, and some changes occurred across all genotypes, whereas others were genotype‐specific or observed in only a subset of genotypes.

**Fig. 2:**
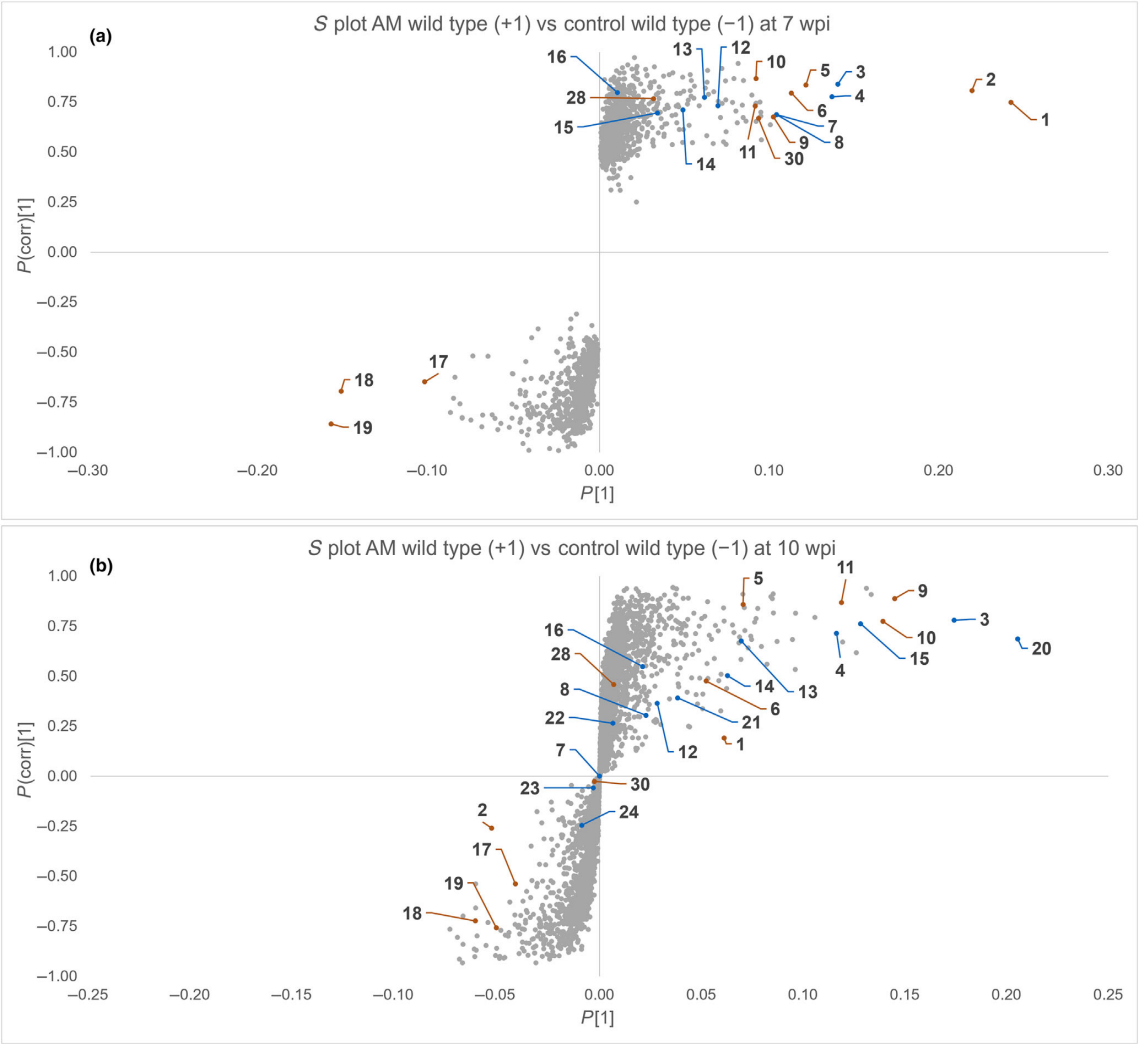
*S* plots of *Lotus japonicus* wild‐type (WT) arbuscular mycorrhizal (AM) roots (+1) vs control (−1) roots (a) harvested at 7 wk post inoculation (wpi) and (b) harvested at 10 wpi with highlighted marker compounds (blue: identified compounds, orange: postulated compounds).

Orthogonal partial least squares discriminant analysis regression was used to visualize differences between control and AM roots as *S* plots of mass spectral features (Figs 2, S4, S5). In the WT and in *ccamk‐3*, *ccamk‐13*, *ram1‐3* (7 wpi), *ram1‐4* (10 wpi), *ram2‐1*, and *ram2‐2*, more features were increased with AM than in *cyclops‐3* and *cyclops‐4*, indicating that the *cyclops* metabolome reacts less strongly to AM inoculation (Table 1). In roots harvested at 10 wpi, fewer MS features were detected overall, but 93% of them passed through the application of significance filters, whereas at 7 wpi, 15% passed the filters (Table 1). Therefore, the absolute and relative numbers of differentially accumulating features using filters according to OPLS‐DA regression rose with time.

To assess the magnitude and reliability of those increases, cut‐offs for the OPLS‐DA loadings value of |*P*[1]| > 0.1 and for the correlation value of |*P*(corr)[1]| > 0.5 are commonly applied in metabolomics (Xue *et al*., 2012; Bao *et al*., 2016; de Clercq *et al*., 2016; Hamany Djande *et al*., 2021; Nizioł *et al*., 2021). Applying these cut‐offs gave 9 candidate compounds that increased with AM in WT roots after 7 wpi and 12 candidate compounds that increased after 10 wpi (Table 1; for their mass spectral IDs, see Table S3). In the mutants, between 1 and 20 compounds reliably increased with AM after 7 wpi, and between 4 and 12 compounds increased after 10 wpi (Tables 1, S4), indicating that all mutants showed a metabolic response to root colonization.

To identify candidate metabolites associated with an intact symbiosis, we focused on isolating single compounds from WT roots (Table S2). The *S* plot of WT *L. japonicus* roots harvested at 7 wpi in Fig. 2a revealed ion *m/z* 297.0766 (**1**) as the most strongly upregulated compound in AM roots at 7 wpi. In addition, the ions *m/z* 563.0982 (**2**), 351.0870 (**3**), 283.0245 (**4**), 609.1396 (**5**), 595.1235 (**6**), 283.0609 (**7** and **8**), and 343.0821 (**9**) were reliably correlated with AM roots harvested at 7 wpi. By contrast, the three ions 563.0981 (**17**), 581.1084 (**18**), and 609.1395 (**19**) were most reliably downregulated in AM roots. The *S* plot resulting from roots harvested at 10 wpi (Fig. 2b) demonstrated a positive correlation of *m/z* 455.3529 (**20**), 351.0870 (**3**), 343.0821 (**9**), 327.0872 (**10**), and 311.0558 (**11**) with 10 wpi AM roots. Ions **3** and **4** were strongly increased with AM in both *S* plots. Accurate mass determination facilitated sum formula calculations for **1–11** and **17–20** (Table 2).

**Table 2:** Identified and postulated *S*‐plot marker compounds with their retention times, observed and calculated mass‐to‐charge ratios (*m/z*), sum formulas, collision cross‐sectional areas (CCS), and diagnostic fragments.

Compound	Retention time (min)	Observed *m/z* (Da)	Calculated *m/z* (Da)	Δ *m/z* (ppm)	Sum formula	CCS (Å^2^)	*m/z* of diagnostic fragments (Da (%))
*Hydroxy‐dimethoxypterocarp‐6a‐ene* (**1**)^a^, ^b^	7.20	297.0766	297.0768	−0.7	C_17_H_14_O_5_	168.5	282.0531 (50), 267.0296 (100), 239.0348 (34), 211.0399 (18)
**2** ^a^, ^b^	6.88	563.0972	563.0984	−2.1	C_32_H_20_O_10_	223.8	548.0744 (18), 535.1019 (23), 520.0795 (11), 413.0657 (71), 398.0420 (21), 385.0709 (15), 370.0472 (26), 267.0297 (8)
Lupinalbin B (**3**)^a^, ^b^	7.21	351.0870	351.0874	−1.1	C_20_H_16_O_6_	185.0	323.0923 (10), 298.1560 (16), 184.0161 (20)
Lupinalbin A (**4**)^a^, ^b^	5.35	283.0245	283.0248	−1.1	C_15_H_8_O_6_	156.4	255.0293 (37), 239.0341 (10), 227.0342 (27), 173.0239 (28)
**5** ^a^	7.43	609.1392	609.1402	−1.6	C_34_H_26_O_11_	251.7	594.1461 (11), 581.1441 (15), 566.1200 (10), 551.0982 (12), 535.1026 (14), 520.0788 (8)
**6** ^a^, ^b^	6.80	595.1244	595.1246	−0.3	C_33_H_24_O_11_	243.2	580.1022 (7), 567.1288 (10), 536.1102 (7), 520.0810 (9), 270.0533 (10)
Acacetin (**7**)^a^, ^b^	6.16	283.0609	283.0612	−1.1	C_16_H_12_O_5_	163.9	212.0455 (5)
Biochanin A (**8**)^a^, ^b^	6.29	283.0609	283.0612	−1.1	C_16_H_12_O_5_	164.8	267.0292 (33), 239.0343 (23), 211.0393 (18)
*Dihydroxytrimethoxyarylbenzofuran* **9** ^a^, ^b^	6.63	343.0821	343.0823	−0.6	C_18_H_16_O_7_	180.0	328.0570 (11), 315.0872 (37), 313.0343 (12), 300.0637 (100), 298.0114 (25), 285.0400 (88), 267.0293 (19), 257.0451 (47), 242.0218 (40), 198.0316 (13)
*Hydroxytrimethoxypterocarp‐6a‐ene* **10** ^a^, ^b^	6.68	327.0872	327.0874	−0.6	C_18_H_16_O_6_	177.9	312.0636 (60), 297.0400 (100), 282.0171 (34), 269.0447 (13), 254.0211 (15), 226.0271 (11)
*Hydroxydimethoxycoumestan* **11** ^a^, ^b^	6.27	311.0558	311.0561	−0.9	C_17_H_12_O_6_	168.6	296.0324 (100), 281.0088 (25), 253.0137 (89), 225.0186 (20), 209.0247 (7), 197.0242 (15)
Coumestrol (**12**)^a^, ^b^	5.03	267.0294	267.0299	−1.9	C_15_H_8_O_5_	152.0	239.0344 (7), 211.0396 (8)
5,7‐Dihydroxy‐4′‐methoxycoumaronochromone (lotuschromone, **13**)^a^, ^b^	6.75	297.0401	297.0405	−1.3	C_16_H_10_O_6_	160.9	282.0163 (100), 254.0211 (86), 226.0265 (33), 198.0315 (16)
Ayamenin D (**14**)^a^, ^b^	5.43	313.0349	313.0354	−1.6	C_16_H_10_O_7_	165.4	298.0113 (100), 269.0086 (30), 242.0211 (6)
4‐Hydroxy‐2‐(2′‐hydroxy‐4′‐methoxyphenyl)‐6‐methoxybenzofuran‐3‐carbaldehyde (lotusaldehyde, **15**)^a^, ^b^	5.61	313.0713	313.0718	−1.6	C_17_H_14_O_6_	173.1	285.0764 (36), 270.0531 (95), 255.0296 (100), 227.0348 (51), 183.0448 (23)
7‐Hydroxy‐3,9‐dimethoxypterocarp‐6a‐ene (lotuscarpene, **16**)^a^, ^b^	5.53	297.0764	297.0768	−1.3	C_17_H_14_O_5_	168.5	282.0528 (46), 267.0293 (100), 255.0293 (22), 239.0344 (33), 211.0394 (22)
**17** ^a^, ^b^	8.30	563.0990	563.0984	+1.1	C_32_H_20_O_10_	223.8	548.0756 (16), 535.1040 (22), 520.0792 (8), 440.0549 (6), 413.0664 (65), 398.0425 (19), 385.0718 (12), 370.0481 (21)
**18** ^a^, ^b^	6.57	581.1084	581.1089	−0.9	C_32_H_22_O_11_	235.7	298.0479 (30), 270.0531 (24), 137.0243 (51)
**19** ^a^, ^b^	7.60	609.1395	609.1402	−1.1	C_34_H_26_O_11_	251.6	593.1439 (12), 579.1295 (33), 562.0912 (20), 535.1032 (12), 520.0795 (7), 453.1711 (47), 317.1552 (20)
Betulinic acid (**20**)^b^	9.10	455.3529	455.3531	−0.4	C_30_H_48_O_3_	218.2	381.3526 (6)
Vestitol (**21**)^b^	5.60	271.0974	271.0976	−0.7	C_16_H_16_O_4_	164.0	256.0736 (22), 241.0503 (27), 197.0603 (15), 147.0449 (15), 135.0451 (30), 109.0293 (18)
Isoliquiritigenin (**22**)^b^	5.38	255.0660	255.0663	−1.2	C_15_H_12_O_4_	160.0	213.0554 (8), 135.0085 (44), 119.0501 (100), 91.0189 (13)
Formononetin (**23**)^b^	5.63	267.0660	267.0663	−1.1	C_16_H_12_O_4_	162.0	252.0423 (100), 223.0398 (45), 195.0450 (27)
Catechin (**24**)^b^	2.95	289.0714	289.0718	−1.4	C_15_H_14_O_6_	156.2	245.0820 (90), 203.0714 (52), 187.0401 (18), 123.0454 (26), 109.0295 (28)
*7*,*9‐Dihydroxy‐3‐methoxypterocarp‐6a‐ene* (**28**)^a^, ^b^	5.24	283.0608	283.0612	−1.4	C_16_H_12_O_5_	163.0	268.0373 (100), 240.0412 (7), 239.0347 (8), 224.0476 (25), 211.0390 (11)
**30** ^a^, ^b^	7.19	565.1136	565.1140	−0.7	C_32_H_22_O_10_	244.2	547.1032 (100), 532.0787 (33), 523.1025 (42), 283.0576 (25), 267.0296 (24)

Names in italics indicate compounds postulated based on MS^e^ data.

^a^ Identified in the 7‐wk post inoculation (wpi) *S* plot.

^b^ Identified in the 10‐wpi *S* plot.

Although there was no significant difference in the root colonization after 7 and 10 wk (refer to Table S2), the most reliably upregulated metabolites changed between the two harvesting time points, with compounds **1**–**6** being the most significant features in mycorrhizal roots harvested at 7 wpi and compounds **20**, **3**, **9**, and **10** being the most significant features in mycorrhizal roots harvested at 10 wpi. This indicates a temporal change in the root metabolome during AM.

For compound identification, we created an in‐house database using Progenesis SDF Studio (Table S4). The database contains structural information on 319 compounds described in *L. japonicus* (Ranner *et al*., 2023) and 149 additional metabolites from closely related species or structural derivatives thereof. Based on the *m/z* values of precursor and fragment ions, 459 database compounds and *in silico* fragments were assigned to 168 distinct metabolites from roots harvested at 7 wpi because isomers with identical sum formulae were frequently matched to the same metabolite (Table S5). In roots harvested at 10 wpi, 186 distinct metabolites were linked to 427 database compounds (Table S6). Co‐chromatography of authentic standard compounds with a well‐colonized *L. japonicus* root extract sample verified the assignment of lupinalbin B (**3**), acacetin (**7**), biochanin A (**8**), coumestrol (**12**), betulinic acid (**20**), vestitol (**21**), isoliquiritigenin (**22**), formononetin (**23**), and catechin (**24**). Further unknown metabolites **4**, **13**–**16** were isolated to elucidate their structures.

### Metabolite isolation from *L. japonicus* roots reveals three novel polyphenols

**Fig. 3:**
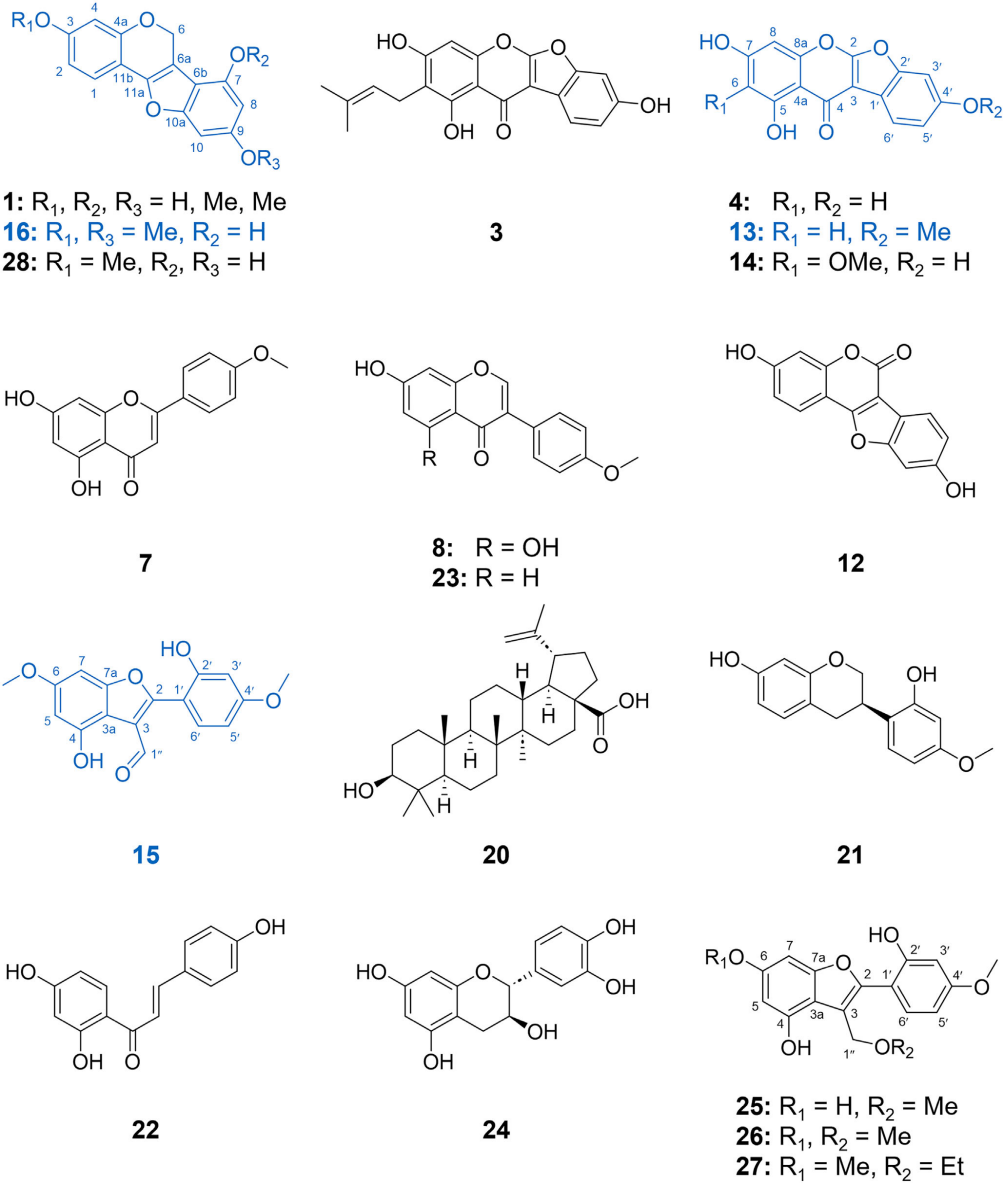
Chemical structures of a postulated hydroxy‐dimethoxypterocarp‐6a‐ene (**1**)*; 7‐hydroxy‐3,9‐dimethoxypterocarp‐6a‐ene (lotuscarpene, **16**)**, 7,9‐dihydroxy‐3‐methoxypterocarp‐6a‐ene (**28**)**; lupinalbin B (**3**)*; lupinalbin A (**4**)*; 5,7‐dihydroxy‐4′‐methoxycoumaronochromone (lotuschromone, **13**)**; ayamenin D (**14**)*; acacetin (**7**)*; biochanin A (**8**); formononetin (**23**); coumestrol (**12**)*; 4‐hydroxy‐2‐(2′‐hydroxy‐4′‐methoxyphenyl)‐6‐methoxybenzofuran‐3‐carbaldehyde (lotusaldehyde, **15**)**; betulinic acid (**20**); vestitol (**21**); isoliquiritigenin (**22**); catechin (**24**)*; 4,6‐dihydroxy‐2‐(2′‐hydroxy‐4′‐methoxyphenyl)‐3‐(methoxymethyl)benzofuran (**25**)***; 4‐hydroxy‐2‐(2′‐hydroxy‐4′‐methoxyphenyl)‐6‐methoxy‐3‐(methoxymethyl)benzofuran (**26**)***; and 4‐hydroxy‐2‐(2′‐hydroxy‐4′‐methoxyphenyl)‐6‐methoxy‐3‐(ethoxymethyl)benzofuran (**27**)***. *Described in *Lotus japonicus* for the first time; **Novel compound; ***Novel artefact.

Compounds *m/z* 283.0245 (**4**) and 313.0349 (**14**) were extracted from *L. japonicus* roots using MeOH and purified by MPLC and HPLC. Ultraperformance liquid chromatography‐electrospray ionization‐ion mobility‐time‐of‐flight‐mass spectrometry and NMR spectroscopy (Figs S6, S7; Table S7) facilitated the identification of the coumaronochromones lupinalbin A (**4**) and ayamenin D (**14**, Fig. 3). Mass spectrometry and ^1^H and ^13^C NMR data from **4** and ^1^H NMR data from **14** were consistent with previously published results (Hanawa *et al*., 1991a,b; Selepe *et al*., 2011; Ateba *et al*., 2014).

Methanolic extraction of *L. japonicus* control roots and purification with MPLC and HPLC yielded compound **13** with a pseudo‐molecular ion peak of *m/z* 297.0401 [M–H]^−^, corresponding to the molecular formula C_16_H_10_O_6_. Characteristic high collision energy MS^e^ fragments (Fig. S8h) indicated the cleavage of a methyl group ([M–H–CH_3_]^−^, *m*/*z* 282), a cascade of triple neutral CO loss (*m*/*z* 283 → 254 → 226 → 198), and a CO_2_ neutral loss (*m*/*z* 226 → 182). Remarkably, following the CH_3_ loss, the fragmentation pattern of **13** resembled that of lupinalbin A (**4**), suggesting that **13** is a methylated derivative of **4** (Figs S6g, S8h). Nuclear magnetic resonance spectroscopy (Fig. S8) facilitated the full structural elucidation of **13** (Notes S1). The full assignment of ^1^H and ^13^C NMR signals is available in Table S7. Accordingly, **13** was identified as the novel compound 5,7‐dihydroxy‐4′‐methoxycoumaronochromone and named lotuschromone.

Metabolites **15** and **16** were obtained through the methanolic extraction of mycorrhizal *L. japonicus* roots and purified by chromatography. The pseudo‐molecular ion peak of **15** with *m/z* 313.0713 energy MS^e^ fragments (Fig. S9i) revealed an initial neutral CO loss (*m/z* 313 → 285), a double CH_3_ loss (*m/z* 285 → 270 → 255), and a neutral CO and CO_2_ loss (*m/z* 255 → 227 → 183). By means of extensive NMR spectroscopy (Fig. S9; Table S7), **15** was identified as the novel compound 4‐hydroxy‐2‐(2′‐hydroxy‐4′‐methoxyphenyl)‐6‐methoxybenzofuran‐3‐carbaldehyde (Notes S2) and named lotusaldehyde. For **16**, a pseudo‐molecular ion peak with *m/z* 297.0763 [M–H]^−^ was observed, suggesting the molecular formula C_17_H_14_O_5_. High‐energy MS^e^ fragmentation (Fig. S10i) indicated two methoxy functions in the molecule (*m/z* 297 → 282 → 267) and a double neutral CO loss (*m/z* 267 → 239 → 211). The chemical structure of **16** was identified via NMR spectroscopy (Fig. S10; Table S7; Notes S3) to be 7‐hydroxy‐3,9‐dimethoxypterocarp‐6a‐ene, a novel metabolite, which was named lotuscarpene.

Methanolic extraction of mycorrhizal *L. japonicus* roots and HPLC purification yielded three more compounds, **25**, **26**, and **29**, which were structurally characterized using UPLC‐ToF‐MS and NMR spectroscopy (Figs S11–S13). Low‐energy MS^e^ analysis of **25** showed a pseudo‐molecular ion peak of *m/z* 315.0872 [M–H]^−^ (calculated: 315.0874; −0.6 ppm) corresponding to a sum formula of C_17_H_16_O_6_. Diagnostic fragments indicated a neutral loss of MeOH (*m/z* 283), a CH_3_ loss (*m/z* 268), and a CO loss (*m/z* 240). **26** contained an additional methyl group with *m/z* 329.1029 [M–H]^−^ (calculated: 329.1031; −0.6 ppm) and a calculated sum formula of C_18_H_18_O_6_. It mirrored the fragmentation pattern of **25** but with a double CH_3_ loss (*m*/*z* 282 and 267; Figs S11i, S12i).

One‐ and two‐dimensional nuclear magnetic resonance spectroscopy of **26** displayed close similarities to the signals of lotusaldehyde (**15**), with the main difference being the lack of an aldehyde function but the presence of an additional methoxymethyl function in the structure (Fig. S12; Table S7). The methoxymethyl group was located at position C(3) instead of the aldehyde, revealing **26** as 4‐hydroxy‐2‐(2′‐hydroxy‐4′‐methoxyphenyl)‐6‐methoxy‐3‐(methoxymethyl)benzofuran. Nuclear magnetic resonance spectroscopy (Fig. S11; Table S7) confirmed that **25** was a derivative of **26** bearing a hydroxy function instead of a methoxy function at position C(6). Thus, **25** was characterized as 4,6‐dihydroxy‐2‐(2′‐hydroxy‐4′‐methoxyphenyl)‐3‐(methoxymethyl)benzofuran.

Compound **29** indicated a low‐energy MS^e^ pseudo‐molecular ion with *m/z* 597.1396 [M–H]^−^ (calculated: 597.1402; −1.0 ppm) and a sodium adduct ion with *m/z* 619.1215 [M + Na − 2H]^−^ (calculated: 619.1222; −1.1 ppm) with a proposed sum formula of C_33_H_26_O_11_ and a double‐bond equivalent (DBE) value of 21. As observed for **25** and **26**, neutral loss of MeOH was observed (*m/z* 597 → 565) along with an H_2_O loss (*m/z* 565 → 547) and a double CH_3_ loss (*m/z* 547 → 532 → 517). Fragment *m/z* 283 indicated that the ion *m/z* 597 splits into two isobaric fragments following MeOH loss and a subsequent CH_3_ loss (*m/z* 283 → 268; Fig. S13h). Nuclear magnetic resonance spectroscopy revealed the dimeric nature of the structure (Fig. S13; Table S7; Notes S4). The ‘first part’ of the component was disclosed as 2‐(hydroxymethoxyphenyl)‐3‐(methoxymethyl)benzofuran, similar to **25** and **26**. The ‘second part’ may be a tetracyclic pterocarpan structure with a double linkage of both moieties, as in procyanidin A_1_ and A_2_ (Schilling *et al*., 1973). We could not deduce the exact linkage because decisive HMBC correlations were missing, and further HMBC experiments with varying coupling constants as well as rotating‐frame overhauser enhancement spectroscopy (ROSEY) did not provide more insights. Therefore, three structural predictions were made (Fig. S14; Notes S4). Neither **25**, **26**, nor **29** were detected in the 7 wpi and 10 wpi root samples.

### Pterocarp‐6a‐enes are degraded in alcoholic solutions

The absence of **25**, **26**, and **29** from the MeCN extracts of *L. japonicus* roots and previous reports of aryl methoxymethylbenzofurans as photolytic pterocarp‐6a‐ene artefacts (Perrin & Bottomley, 1962; Dewick, 1977) suggested that **25**, **26**, and **29** were generated during the compound isolation from methanolic *L. japonicus* root extracts. To test this assumption, an aliquot of *L. japonicus* MeCN root extract containing the hypothetical precursor of **26** – the pterocarp‐6a‐ene lotuscarpene (**16**) – was lyophilized; redissolved in MeOH, EtOH, and MeCN; and kept under UV light (254 nm) for 24, 48, and 72 h. At each time point, the sample was analysed by UPLC‐ToF‐MS to determine the peak responses of **25**, **16**, and the hypothetical 3‐ethoxymethylbenzofuran artefact **27** (C_19_H_20_O_6_; *m/z* 343.1182 [M−H]^−^; calculated: 343.1187; −1.5 ppm; RT: 6.90 min, Fig. 3), expected to be formed in EtOH. **27** was tentatively assigned based on MS^e^ data (Fig. S15). The high‐energy MS^e^ spectrum of **27** showed a loss of 46 Da (*m/z* 343 → 297), revealing the neutral loss of EtOH from the compound. Over time, a decrease in the signal response of **16** in both MeOH and EtOH was accompanied by an increase in the response of **26** in MeOH and **27** in EtOH (Fig. S16a), supporting the notion that **26** and **27** are the degradation products of **16**, where the alcohol was added at position C(6) to open the ring. Additionally, the signal response of **16** remained constant in the MeCN solution.

Similarly, normalized responses of **25** and **29** increased in MeOH over time and were not observed in MeCN (Fig. S16b,c). A precursor of **25** may be postulated as 7,9‐dihydroxy‐3‐methoxypterocarp‐6a‐ene, and a corresponding ion (**28**, C_16_H_12_O_5_; *m/z* 283.0608 [M−H]^−^) was detected in 7 and 10 wpi *L. japonicus* roots (Fig. 3). Compound **28** exhibited a fragmentation behaviour similar to that of **16**, with a CH_3_ loss (*m/z* 268) and a double neutral CO loss (*m/z* 239 and 211; Fig. S17). It was degraded during storage in MeOH or EtOH but remained stable in MeCN (Fig. S16). A potential precursor of **29** lacking the methoxymethyl function would have a sum formula of C_32_H_22_O_10_ and a calculated mass of *m/z* 565.1140 [M−H]^−^. A corresponding ion with *m/z* 565.1136 [M−H]^−^ (**30**, Table 2; Fig. S18) was observed in both *S* plots (Fig. 2). The normalized *m/z* response of **30** decreased in MeOH or EtOH and remained constant in MeCN (Fig. S16c). Thus, we confirmed that **25**, **26**, and **29** are breakdown products generated during compound isolation.

### ToF‐MS^e^ fragmentation allows the postulation of additional polyphenols

The most significantly AM‐induced compound from 7 wpi roots, compound **1** (C_17_H_14_O_5_; *m*/*z* 297.0766 [M−H]^−^; RT: 7.20 min), exhibited the same fragmentation pattern as **16** (RT: 5.53 min) with almost identical diagnostic fragments and fragment intensities (Table 2; Fig. S19). We were not able to obtain **1** as a pure compound. Consistent with the chemical behaviour of **16**, MeOH and EtOH treatment under UV light completely degraded **1** (Fig. S16d), explaining why its isolation from methanolic *L. japonicus* root extract was not achievable. The *m/z* response levels of **1** remained constant during storage in MeCN. Therefore, we postulated that **1** is a constitutional isomer of **16**, as a hydroxy‐dimethoxypterocarp‐6a‐ene (Fig. 3).

We also made structural predictions for compounds **9** (C_18_H_16_O_7_; *m*/*z* 343.0821 [M−H]^−^), **10** (C_18_H_16_O_6_; *m*/*z* 327.0872 [M−H]^−^), and **11** (C_17_H_12_O_6_; *m*/*z* 311.0558 [M−H]^−^), which turned out to be unstable during isolation. For compound **9** (Fig. S20), fragmentation started with a neutral CO loss (*m/z* 315), a double CH_3_ loss (*m/z* 300 and 285), followed by another CO loss (*m/z* 257), another CH_3_ loss (*m/z* 242), and a CO_2_ loss (*m/z* 198). Therefore, a polyphenol compound with three methoxy groups was deduced. The initial CO loss was also characteristically recorded for lotusaldehyde (**15**), and the overall fragmentation pattern with a final CO_2_ loss was highly similar. Therefore, we postulated that **9** is a derivative of **15** with an additional methoxy function, that is, dihydroxy‐trimethoxy‐arylbenzofuran aldehyde. UV stability testing showed no photolytic degradation for both aldehydes **9** and **15** (Fig. S16e).

The fragmentation of **10** (Fig. S21) was initiated with a double CH_3_ loss (*m/z* 312 and 297). The ion *m/z* 254 was formed after CH_3_ (*m/z* 282) and CO loss or after CO (269) and CH_3_ loss. The DBE value of 11 suggested two aromatic rings in the core structure and either two more rings and a double bond to form a pterocarpene or one more ring and two double bonds to form an (iso)flavonoid, both with one hydroxy and three methoxy functions. Because the fragmentation pattern was similar to that of pterocarp‐6a‐enes **1** and **16**, it seemed more likely that **10** was a pterocarpene. The normalized *m/z* signal response of **10** remained constant in MeCN for up to 72 h but declined in MeOH and EtOH, indicating pterocarp‐6a‐ene degradation (Fig. S16d). Therefore, we postulated that **10** is a hydroxytrimethoxypterocarp‐6a‐ene.

The MS fragmentation of **11** (Fig. S22) revealed a double CH_3_ loss (*m/z* 296 and 281) followed by a triple CO loss (*m/z* 253, 225, and 197). Fragment intensities were similar to those of the corresponding signals for **13** (see Table 2). Therefore, it is likely that **11** contains two aryl rings with one hydroxy and two methoxy functions in a tetracyclic structure, including a pyrone ring. A 4‐pyrone ring would produce a coumaronochromone with one methyl group more than in **13**. However, **11** had a lower RT (6.27 min) than **13** (6.75 min), indicating a more polar compound. Thus, we speculated that **11** is a coumestan containing a 2‐pyrone ring. It has been suggested that arylbenzofuran aldehydes such as lotusaldehyde (**15**) are derived from coumestans (Macías *et al*., 1999), which suggests that **11** is a precursor of **15**. Therefore, we postulated that **11** is 7‐hydroxy‐3,9‐dimethoxycoumestan.

### Effect of AM gene mutations on the marker polyphenols

Although the analysed mutants *ccamk*, *cyclops*, *ram1*, and *ram2* are impaired at different stages of AM formation, AM fungal presence affected their metabolome. Applying the same reliability thresholds for the *S*‐plot analysis as for the WT, between 1 and 20 features were significantly increased in AM mutant roots as compared to their controls (Tables 1, S3). Even though no general or specific ‘mutant markers’ emerged, a common accumulation of varying polyphenolics was observed. For example, compound **1**, which was suggested to be a pterocarpene, and lupinalbin B (**3**) was found to be on average more abundant in AM WT roots than in control WT roots harvested at 7 wpi (Fig. S23). Both compounds also accumulated in all mutant roots regardless of AM. Interestingly, compound **1** almost consistently accumulated to higher levels in control roots across all mutants than the WT (Fig. S23a), suggesting that the mutations may lift regulatory breaks on the accumulation of this compound in the absence of AM. Compound **3** consistently accumulated to higher levels in control roots of both *ram1* and *ram2* mutants than the WT (Fig. S23b).

Most of the other compounds passing the reliability thresholds were not reproducibly changed in both alleles of each mutant, but some of them were induced in one of the allelic mutants. However, some unidentified features behaved reproducibly, as for example, ions *m/z* 681.1050 and *m/z* 710.1082, which accumulate more in the WT control roots than the control roots of *ram1* and *ram2* mutants harvested at 7 wpi (Fig. S24). To further evaluate changes in the metabolome of WT and mutant roots, quantitative data using internal standards and calibration curves instead of signal intensities, as shown here, will be helpful in the future.

### Expression of genes involved in polyphenol biosynthesis correlates with metabolome data

Several polyphenols were increased in mycorrhizal *L. japonicus* roots (Table S3) and genes involved in polyphenol biosynthesis are known to be induced by AM (Harrison & Dixon, 1994). To understand whether polyphenol accumulation in *L. japonicus* during an AM symbiosis is backed up by transcriptome data, we compared transcriptome changes in roots colonized by *R. irregularis* obtained by RNA‐sequencing (Table S8) with the metabolomics data by analysing their correlation. Annotated genes were filtered using *Arabidopsis thaliana* IDs from the Kyoto Encyclopedia of Genes and Genomes (KEGG) database of genes involved in polyphenol metabolism (Kanehisa & Goto, 2000), applying the significance (*P*‐value < 0.05) and fold change (> 1.5) thresholds.

**Fig. 4:**
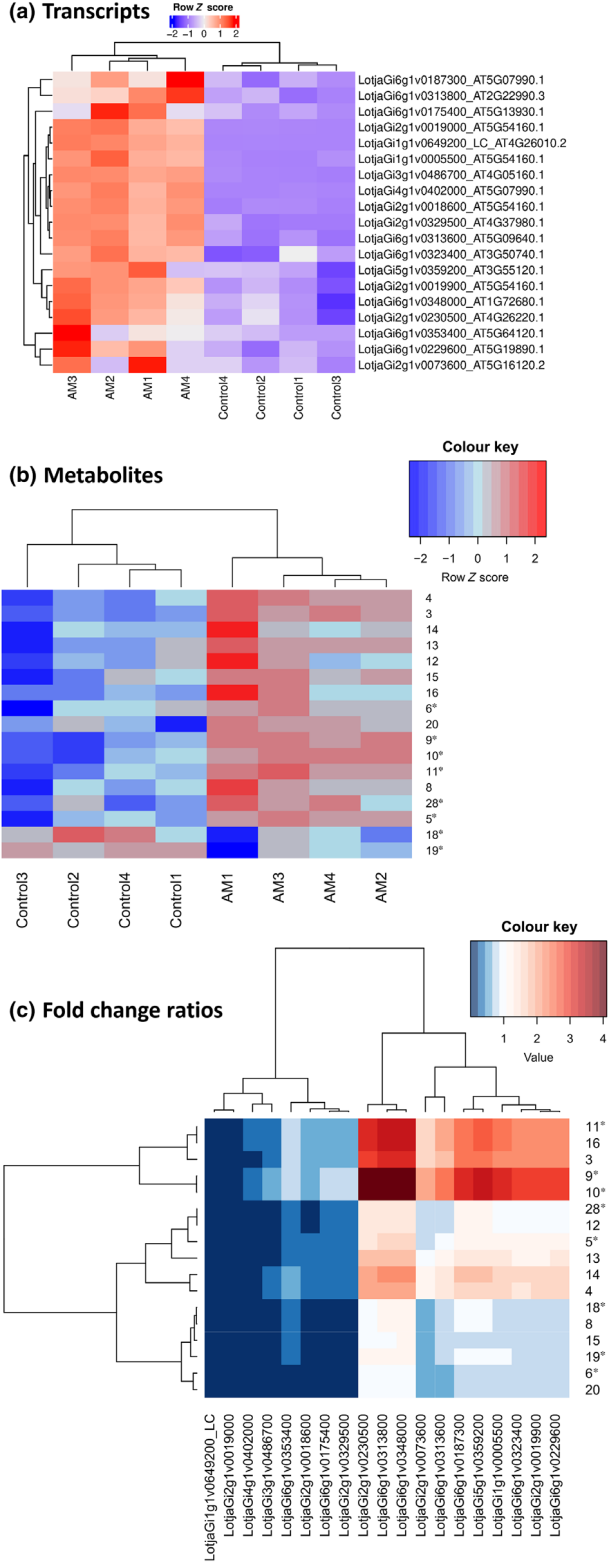
Heatmap visualization of pre‐filtered (*P*‐value < 0.05; fold change > 1.5) (a) *Lotus japonicus* genes (*n* = 19) and (b) nine identified and eight postulated metabolites across four control and four arbuscular mycorrhizal (AM) root samples, with cluster analysis showing a clear distinction between AM and control samples. (c) Heatmap displaying the fold change ratios of metabolites to genes and cluster analysis showing the associations between the two omics data sets. Blue areas indicate a higher fold change of the gene, whereas red areas show a higher fold change of the metabolite. The gene cluster on the left is induced more strongly by AM than the annotated metabolites. The gene cluster on the right is induced by AM in a similar manner as the metabolite cluster comprising metabolites **20**–**28**, whereas the metabolite cluster comprising metabolites **10**, **9**, **3**, **16**, and **11** exceeds the abundance of the genes. Identities of metabolites denoted with an asterisk (*) were predicted based on mass spectral analysis. Compound identifications: lupinalbin B (**3**), lupinalbin A (**4**), biochanin A (**8**), dihydroxytrimethoxyarylbenzofuran (**9**), hydroxytrimethoxypterocarp‐6a‐ene (**10**), hydroxydimethoxycoumestan (**11**), coumestrol (**12**), lotuschromone (**13**), ayamenin D (**14**), lotusaldehyde (**15**), lotuscarpene (**16**), betulinic acid (**20**), and 7,9‐dihydroxy‐3‐methoxypterocarp‐6a‐ene (**28**). Compounds **5**, **6**, **18**, and **19** are unidentified dimers.

Nineteen polyphenol pathway genes (Fig. 4a; Table S9) were clearly induced in *L. japonicus* WT roots upon AM. Nine identified (**3**, **4**, **8**, **12**–**16**, **20**) and eight postulated metabolites (**5**, **6**, **9**–**11**, **18**, **19**, **28**) out of 26 annotated compounds were significantly more abundant in AM samples than in control samples at 10 wpi (Fig. 4b), as also shown in the *S*‐plot trends. To assess the relative degree of AM‐derived polyphenol and gene accumulation, fold change ratios were calculated for each metabolite and gene (Fig. 4c). One gene cluster (Fig. 4c, left) was induced more strongly than the corresponding metabolites. LotusBase annotation (Mun *et al*., 2016) suggested that this cluster comprises two genes (LotjaGi1g1v0649200_LC and LotjaGi6g1v0353400) encoding peroxidases, one gene encoding a P450 monooxygenase (LotjaGi4g1v0402000), one gene encoding a chalcone synthase (LotjaGi6g1v0175400), and two *O*‐methyltransferase genes (LotjaGi2g1v0019000 and LotjaGi2g1v0018600), which are interesting candidates for genetic analysis in the future. In summary, the trends observed by metabolomics were confirmed by transcriptomics data.

### Several identified polyphenols and their biosynthetic precursors inhibit *R. irregularis* spore germination

**Fig. 5:**
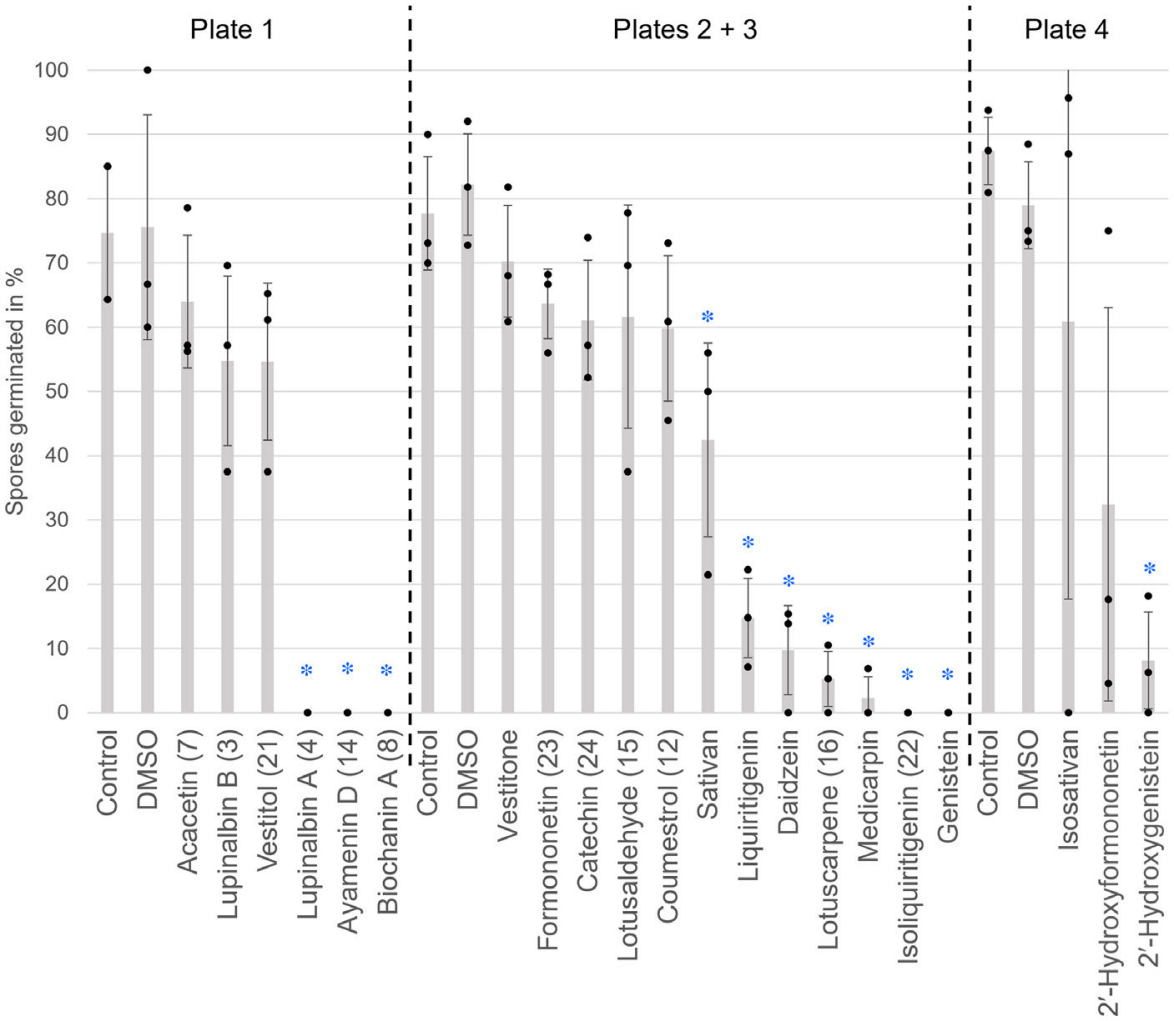
Inhibition of *Rhizophagus irregularis* spore germination by selected polyphenols (added at 10 μmol l^−1^, based on Bécard *et al*., 1992). Plate numbers indicate individual 24‐well plates, in which each replicate assay was run in one individual well. Dashed lines separate experiments performed at different points in time with their own pure medium and dimethyl sulfoxide (DMSO) controls. Grey bars show the mean spore germination, with black dots representing individual results and error bars representing SD. Blue asterisks (*) indicate significant (α = 0.05; *n* = 3; determined by Tukey's test) differences in comparison with pure medium and DMSO controls.

To understand whether AM‐induced *L. japonicus* polyphenols could affect the fungus, we examined *R. irregularis* spore germination upon treatment with AM‐induced (**3**, **4**, **7**, **8**, **12**, **14**–**16**) and other polyphenols from *L. japonicus* (**21**–**24**). We included biosynthetic precursors and derivatives thereof, including daidzein, medicarpin, and sativan, which were previously identified in *L. japonicus* (Kaducová *et al*., 2019), as well as genistein, 2′‐hydroxyformononetin, 2′‐hydroxygenistein, isosativan, and vestitone. In comparison with pure *R. irregularis* growth medium and DMSO solvent control treatments, almost half of the compounds did not affect spore germination. However, **4**, **8**, **14**, **16**, **22**, liquiritigenin, medicarpin, daidzein, genistein, 2′‐hydroxygenistein, and sativan significantly inhibited spore germination (α = 0.05; Fig. 5), suggesting that these compounds could potentially have a negative effect on AM development if they were released into the rhizosphere or possibly also towards intra‐radical fungal structures.

## Discussion

Using untargeted metabolomics, we detected substantial secondary metabolome changes in *L. japonicus* WT roots upon colonization by *R. irregularis*. In roots harvested at 7 wpi, 15.0% of the total MS features changed upon AM (Table 1). Almost all detected MS features were affected by AM. When applying reliability thresholds to identify reliably changed compounds, it was evident that symbiosis more significantly affected a larger amount of the metabolome in older plants (10 wpi), although the degree of colonization was not significantly different after 7 and 10 wk. This suggests that AM affects the metabolome differently throughout an ongoing symbiosis. Time course metabolite monitoring may be required to gain further insights into the temporal dynamics of AM influence on the metabolome.

We observed that mutations in genes required for AM development severely affected the metabolite response to root colonization. Compounds **1** and **3** (lupinalbin B), which are upregulated in WT roots upon AM, accumulated to higher levels in the controls of all mutant roots than WT roots, indicating that their biosynthetic pathway is already activated in the mutants in the absence of AM. Other yet unidentified features were reproducibly reduced in some mutants, such as the ions *m/z* 681.1050 and *m/z* 710.1082 in *ram1* and *ram2* mutant control roots and were not induced upon AM. This indicates that, interestingly, *L. japonicus* RAM1 and RAM2 play a role in modifying the secondary metabolome already in the absence of AM, although their encoding genes are expressed only at low levels under this condition.

The majority of metabolites detected by untargeted metabolomics remain still unknown, impeding their functional characterization and making future structure identification mandatory. Here we identified three novel polyphenols named lotusaldehyde (**15**), lotuschromone (**13**), and lotuscarpene (**16**) in *L. japonicus* roots, which are increased upon AM in the WT but not consistently in all mutants (Fig. S23). In addition, we identified three previously known coumaronochromones, two flavonoids, three isoflavonoids, one coumestan, and one chalcone. We tentatively assigned three pterocarp‐6a‐enes, one arylbenzofuran aldehyde, and one coumestan (Table 2). Arylbenzofurans, coumaronochromones, and pterocarp‐6a‐enes are widespread in legumes (Tahara *et al*., 1985, 1991; Tanaka *et al*., 2002, 2003; Wang *et al*., 2003, 2009; Sobolev *et al*., 2010) but have not been reported in *L. japonicus* before. Generally, they co‐occur with similarly derivatized isoflavonoids and coumestans, suggesting their biosynthetic routes may be linked (Tahara *et al*., 1985; Macías *et al*., 1999; Kraft *et al*., 2001; Lawson *et al*., 2008; Ha *et al*., 2019). To scrutinize this assumption, it will be important to identify the genes involved in their biosynthesis.

### Potential role of the identified compounds during AM symbiosis

Polyphenols are crucial secondary metabolites and are especially highly abundant in legumes where they act, for example, in communication with nitrogen‐fixing rhizobia (Cooper, 2004), but many have phytoalexin activity (Ahuja *et al*., 2012; Khanzada *et al*., 2021). We examined the effect of a selected number of AM‐induced polyphenols or their precursors on AM fungal spores – based on their commercial availability or ease of purification – and observed a significant inhibition of *R. irregularis* spore germination by lupinalbin A (**4**), biochanin A (**8**), ayamenin D (**14**), lotuscarpene (**16**), isoliquiritigenin (**22**), liquiritigenin, medicarpin, daidzein, genistein, 2′‐hydroxygenistein, and sativan at a concentration of 10 μm. Compared with pure medium and DMSO controls, other metabolites including lupinalbin B (**3**), acacetin (**7**), coumestrol (**12**), lotusaldehyde (**15**), formononetin (**23**), catechin (**24**), vestitol (**21**), isosativan, and vestitone showed no significant effect on spore germination. 2′‐Hydroxyformononetin showed inhibition in two of the three biological replicates. Interestingly, in previous studies, genistein inhibited hyphal growth of *Gigaspora margerita* (Chabot *et al*., 1992), which belongs to a different clade within the glomeromycotina than *R. irregularis*, thus showing that it affects distantly related fungi. Acacetin (**7**) inhibited hyphal branching of four fungi including *R. irregularis* (previously known as *Glomus intraradices*), *Glomus mosseae*, *Gigaspora margerita*, *Gigaspora rosea*, and their colonization of tomato roots; however, spore germination was not tested (Scervino *et al*., 2005a,b), thus the results cannot be directly compared with our data. Medicarpin 3‐*O*‐glucoside has been reported to inhibit *R. irregularis* spore germination (Guenoune *et al*., 2001), and we found that the aglycone has the same effect. Coumestrol (**12**) and formononetin (**23**) have been reported to stimulate AM fungal growth (Morandi *et al*., 1984; Nair *et al*., 1991; Siqueira *et al*., 1991; Catford *et al*., 2006; Da Silva *et al*., 2017), and we found no inhibition of *R. irregularis* spore germination. Stimulating effects on the spore germination were not observable, as the germination rate was already close to 100% in the control. However, we found an inhibition by biochanin A (**8**), which has previously been reported to stimulate colonization of *T. repens* by *R. irregularis* (formerly *G. intraradices*) at exogenously applied concentrations of 17.6 μm (Nair *et al*., 1991; Siqueira *et al*., 1991), indicating that the activity depends on the concentration or plant and fungal species involved (Poulin *et al*., 1997). Likewise, daidzein concentrations of 2–5 μm have been reported to stimulate *R. irregularis* spore germination (Kape *et al*., 1992), whereas we found a significant inhibition by 10 μm. To the best of our knowledge, this is the first report of an inhibiting effect of lupinalbin A (**4**), ayamenin D (**14**), lotuscarpene (**16**), isoliquiritigenin (**22**), liquiritigenin, 2′‐hydroxygenistein, and sativan on AM fungal spore germination. Notably, formal prenylation of **4** led to a loss of activity on germination (lupinalbin B, **3**). Polyphenols, such as flavonoids, are exuded into the rhizosphere (Sugiyama & Yazaki, 2014). Thus, inhibitory flavonoids and other polyphenols may be involved in attenuating symbiosis once the root is well colonized through inhibition of further spore germination.

Recently, it has been shown that levels of specific polyphenols are positively and negatively affected in arbuscular mycorrhizal *Elymus nutans* and walnut (*Juglans regia*), suggesting involvement in the cold and drought stress response (Zhang *et al*., 2023; Zou *et al*., 2023). Flavonoids may also take part in AM‐induced plant defense against root pathogens (Veresoglou & Rillig, 2012). It is yet unclear whether the fungus can come into contact with inhibitory polyphenols inside the root. Direct contact with fungal hyphae would only be possible if the compounds accumulate in sufficient concentrations in the apoplast of colonized tissue. However, they may accumulate in regions that are not colonized (e.g., the root elongation zone or in root meristems as suggested for some flavonoids in *Arabidopsis*) (Lewis *et al*., 2011; Chapman & Muday, 2021), and in plant subcellular compartments, which may not be in contact with the fungus, most likely the vacuole (Sugiyama & Yazaki, 2014). Tissue‐specific localization of flavonoids by spatial metabolomics and subcellular localization of compounds using selective stains and/or sensors (that may become available in the future) will be important to understand the relative localization of specific secondary metabolites vs AM fungi inside the root.

The role of triterpenoids, such as betulinic acid (**20**), in AM has not been explored in depth. Foliar analysis of mycorrhizal *M. truncatula* revealed an enhancement of the phenylpropanoid and terpenoid pathway (Adolfsson *et al*., 2017). Some triterpenoid glycosides, termed soyasaponins, are widespread in legumes, which are secreted in the root exudate to potentially boost root nodule symbiosis with nitrogen‐fixing bacteria (Tsuno *et al*., 2018; Buoso *et al*., 2022). While saponins exhibit antifungal, antibacterial, and antiviral activities (Mugford & Osbourn, 2013), their potential interaction with AM fungi has not yet been reported.

### Dimeric polyphenols

While the exact structures of **29** and **30** remain unknown, the isolation of **29** suggests the presence of additional dimeric polyphenols containing at least one pterocarpene moiety. Notably, ions **2** (*m/z* 563.0972, C_32_H_20_O_10_, Fig. S25), **5** (*m/z* 609.1402, C_34_H_26_O_11_, Fig. S26), and **6** (*m/z* 595.1244, C_33_H_24_O_11_, Fig. S27) – which were increased in mycorrhizal roots – and ions **17** (*m/z* 563.0990, C_32_H_20_O_10_, Fig. S28), **18** (*m/z* 581.1084, C_32_H_22_O_11_, Fig. S29), and **19** (*m/z* 609.1395, C_34_H_26_O_11_, Fig. S30) – increased in WT control roots (see Table 2) – share a mass range of 560–610 Da and mass spectral features with **29** and **30**. Multiple losses of CH_3_ and CO were commonly detected for all these compounds, implying a polyphenolic and possibly dimeric nature. A dimeric pterocarpene – hedysarimpterocarpene C – isolated from the roots of *H. multijugum* has been reported by Wang *et al*. (2003) with two identical monomers being linked at C(10). These findings suggest that potentially novel dimeric pterocarpenes are present in *L. japonicus* roots and await their precise structural identification.

### AM‐induced formation of bioactive polyphenols

The correlation of the metabolomics with transcriptomics data revealed a strong induction of two *O*‐methyltransferase genes in AM roots. In combination with the identification of multiple methoxylated polyphenols (**1**, **2**, **5**–**11**, **13**–**19**, **21**, **23**, **28**), this finding suggests a crucial role of polyphenol *O*‐methylation in AM symbiosis. Some of these methylated polyphenols (**8**, **14**, **16**) significantly inhibited fungal spore germination in the present study.

In future studies, a more comprehensive analysis of the polyphenol profile of mycorrhizal plants and also their root exudates could expand our understanding of how plants respond to AM symbiosis by metabolome changes. Importantly, the elucidation of novel structures and of biosynthetic pathways to allow using plant mutants perturbed in the biosynthesis of certain compounds will enable elucidation of their function in AM symbiosis or other aspects of AM‐induced plant performance.

## Competing interests

None declared.

## Author contributions

CG, TDS, and CD designed the study and revised the manuscript. GS and MP provided *L. japonicus* roots and quantified root length colonization; GS performed spore germination assays. JLR performed metabolomics experiments and data analysis, and JLR and AP performed metabolite isolation. AS created Fig. 1. TZ prepared samples for RNA‐seq and provided transcriptomics data, and SS performed omics correlation analysis. JLR wrote the manuscript.

## Disclaimer

The New Phytologist Foundation remains neutral with regard to jurisdictional claims in maps and in any institutional affiliations.

## Supporting information


**Fig. S1** Principal component analysis score loading plots of wild‐type and mutant samples.
**Fig. S2** Score loading plots of UPLC‐ESI‐IM‐ToF‐MS full scan analysis of *Lotus japonicus* roots harvested at 7 wk post inoculation (wpi) of mutants *ccamk‐3*, *ccamk‐13*, *cyclops‐3*, *cyclops‐4*, *ram1‐3*, *ram1‐4*, *ram2‐1*, and *ram2‐2*.
**Fig. S3** Score loading plots of UPLC‐ESI‐IM‐ToF‐MS full scan analysis of *Lotus japonicus* roots harvested at 10 wk post inoculation (wpi) of mutants *ccamk‐3*, *ccamk‐13*, *cyclops‐3*, *ram1‐3*, *ram1‐4*, *ram2‐1*, and *ram2‐2*.
**Fig. S4** *S* plots of *Lotus japonicus* root extracts harvested at 7 wk post inoculation (wpi) comparing mycorrhizal and control samples of mutants *ccamk‐3*, *ccamk‐13*, *cyclops‐3*, *cyclops‐4*, *ram1‐3*, *ram1‐4*, *ram2‐1*, and *ram2‐2*.
**Fig. S5** *S* plots of *Lotus japonicus* root extracts harvested at 10 wk post inoculation (wpi) comparing mycorrhizal and control samples of mutants *ccamk‐3*, *ccamk‐13*, *cyclops‐3*, *ram1‐3*, *ram1‐4*, *ram2‐1*, and *ram2‐2*.
**Fig. S6** ^1^H, ^13^C, COSY, HSQC, and HMBC NMR spectra and low‐ and high‐energy ToF‐MS^e^ spectra of lupinalbin A (**4**).
**Fig. S7** ^1^H, ^13^C, COSY, HSQC, and HMBC NMR spectra and low‐ and high‐energy ToF‐MS^e^ spectra of ayamenin D (**14**).
**Fig. S8** ^1^H, ^13^C, COSY, HSQC, and HMBC NMR spectra, key HMBC correlations, and low‐ and high‐energy ToF‐MS^e^ spectra of 5,7‐dihydroxy‐4′‐methoxycoumaronochromone (lotuschromone, **13**).
**Fig. S9** ^1^H, ^13^C, COSY, HSQC, and HMBC NMR spectra, key HMBC correlations, and low‐ and high‐energy ToF‐MS^e^ spectra of 4‐hydroxy‐2‐(2′‐hydroxy‐4′‐methoxyphenyl)‐6‐methoxybenzofuran‐3‐carbaldehyde (Lotusaldehyde, **15**).
**Fig. S10** ^1^H, ^13^C, COSY, HSQC, and HMBC NMR spectra, key HMBC correlations, and low‐ and high‐energy ToF‐MS^e^ spectra of 7‐hydroxy‐3,9‐dimethoxypterocarp‐6a‐ene (Lotuscarpene, **16**).
**Fig. S11** ^1^H, ^13^C, COSY, HSQC, and HMBC NMR spectra, key HMBC correlations, and low‐ and high‐energy ToF‐MS^e^ spectra of 4,6‐dihydroxy‐2‐(2′‐hydroxy‐4′‐methoxyphenyl)‐3‐(methoxymethyl)benzofuran (**25**).
**Fig. S12** ^1^H, ^13^C, COSY, HSQC, and HMBC NMR spectra, key HMBC correlations, and low‐ and high‐energy ToF‐MS^e^ spectra of 4‐Hydroxy‐2‐(2′‐hydroxy‐4′‐methoxyphenyl)‐6‐methoxy‐3‐(methoxymethyl)benzofuran (**26**).
**Fig. S13** ^1^H, ^13^C, COSY, HSQC, and HMBC NMR spectra and low‐ and high‐energy ToF‐MS^e^ spectra of a dimeric polyphenol artefact (**29**).
**Fig. S14** Structure proposals for the dimeric polyphenol artefact **29**.
**Fig. S15** Low‐ and high‐energy ToF‐MS^e^ spectra of postulated 4‐hydroxy‐2‐(2′‐hydroxy‐4′‐methoxyphenyl)‐6‐methoxy‐3‐(ethoxymethyl)benzofuran (**27**).
**Fig. S16** Normalized response of **1**, **9**, **10**, **15**, **16**, and **25**–**30** in MeOH, EtOH, and MeCN after 0, 24, 48, and 72 h of UV light exposure.
**Fig. S17** Low‐ and high‐energy ToF‐MS^e^ spectra of postulated 7,9‐dihydroxy‐3‐methoxypterocarp‐6a‐ene (**28**).
**Fig. S18** Low‐ and high‐energy ToF‐MS^e^ spectra of postulated dimeric polyphenol **30**, a precursor to isolated artefact **29**.
**Fig. S19** Low‐ and high‐energy ToF‐MS^e^ spectra of a postulated hydroxy‐dimethoxypterocarp‐6a‐ene (**1**).
**Fig. S20** Low‐ and high‐energy ToF‐MS^e^ spectra of a postulated dihydroxy‐trimethoxy aryl benzofuran‐3‐carbaldehyde (**9**).
**Fig. S21** Low‐ and high‐energy ToF‐MS^e^ spectra of a postulated hydroxy trimethoxypterocarp‐6a‐ene (**10**).
**Fig. S22** Low‐ and high‐energy ToF‐MS^e^ spectra of a postulated 7‐hydroxy‐3,9‐dimethoxycoumestan (**11**).
**Fig. S23** Mean normalized abundance of compounds **1**, **3** (lupinalbin B), **13** (lotuschromone), **15** (lotusaldehyde), and **16** (lotuscarpene) in control and AM wild‐type and mutant roots harvested at 7 wpi.
**Fig. S24** Mean normalized abundance of unidentified features *m/z* 681.1050 and *m/z* 710.1082 in control and AM wild‐type and mutant roots harvested at 7 wpi.
**Fig. S25** Low‐ and high‐energy ToF‐MS^e^ spectra of compound **2**.
**Fig. S26** Low‐ and high‐energy ToF‐MS^e^ spectra of compound **5**.
**Fig. S27** Low‐ and high‐energy ToF‐MS^e^ spectra of compound **6**.
**Fig. S28** Low‐ and high‐energy ToF‐MS^e^ spectra of compound **17**.
**Fig. S29** Low‐ and high‐energy ToF‐MS^e^ spectra of compound **18**.
**Fig. S30** Low‐ and high‐energy ToF‐MS^e^ spectra of compound **19**.
**Methods S1** Marker compound isolation.
**Methods S2** Omics correlation analysis.
**Notes S1** Analysis of NMR spectra of 5,7‐dihydroxy‐4′‐methoxycoumaronochromone (lotuschromone, **13**).
**Notes S2** Analysis of NMR spectra of 4‐hydroxy‐2‐(2′‐hydroxy‐4′‐methoxyphenyl)‐6‐methoxybenzofuran‐3‐carbaldehyde (lotusaldehyde, **15**).
**Notes S3** Analysis of NMR spectra of 7‐hydroxy‐3,9‐dimethoxypterocarp‐6a‐ene (lotuscarpene, **16**).
**Notes S4** Analysis of NMR spectra of the dimeric polyphenol artefact **29**.
**Table S1** Chromatographic conditions for compound isolation.

**Table S2** Colonization data including intraradical hyphae, arbuscules, vesicles, total root length colonization, and mean colonization per genotype.
**Table S3** Potential marker candidates in *Lotus japonicus* wild‐type and mutant roots.
**Table S4** Metabolites included in the in‐house *in silico* database.
**Table S5** *In silico* database hits for mass spectral features in 7 wpi root samples.
**Table S6** *In silico* database hits for mass spectral features in 10 wpi root samples.
**Table S7** ToF‐MS^e^ and NMR data of isolated compounds.
**Table S8** Annotated transcriptome data of *Lotus japonicus* AM and control root samples.
**Table S9** Significantly AM‐induced polyphenol biosynthesis genes.
Please note: Wiley is not responsible for the content or functionality of any Supporting Information supplied by the authors. Any queries (other than missing material) should be directed to the *New Phytologist* Central Office.

## Data Availability

RNA‐sequencing reads are available in NCBI with accession no. PRJNA1086535 (https://pmc.ncbi.nlm.nih.gov/articles/PMC2801379/).
