# Supplementary material for: Untargeted metabolomics reveals novel metabolites in Lotus japonicus roots during arbuscular mycorrhiza symbiosis
Source: New Phytol. 2025 Mar 17;246(3):1256–75. doi: 10.1111/nph.70051 (PMC11982791; doi:10.1111/nph.70051)
Supplement: Supplementary file 1 — Fig. S1 Principal component analysis score loading plots of wild‐type and mutant samples. Fig. S2 Score loading plots of UPLC‐ESI‐IM‐ToF‐MS full scan analysis of Lotus japonicus roots harvested at 7 wk post inoculation (wpi) of mutants ccamk‐3, ccamk‐13, cyclops‐3, cyclops‐4, ram1‐3, ram1‐4, ram2‐1, and ram2‐2. Fig. S3 Score loading plots of UPLC‐ESI‐IM‐ToF‐MS full scan analysis of Lotus japonicus roots harvested at 10 wk post inoculation (wpi) of mutants ccamk‐3, ccamk‐13, cyclops‐3, ram1‐3, ram1‐4, ram2‐1, and ram2‐2. Fig. S4 S plots of Lotus japonicus root extracts harvested at 7 wk post inoculation (wpi) comparing mycorrhizal and control samples of mutants ccamk‐3, ccamk‐13, cyclops‐3, cyclops‐4, ram1‐3, ram1‐4, ram2‐1, and ram2‐2. Fig. S5 S plots of Lotus japonicus root extracts harvested at 10 wk post inoculation (wpi) comparing mycorrhizal and control samples of mutants ccamk‐3, ccamk‐13, cyclops‐3, ram1‐3, ram1‐4, ram2‐1, and ram2‐2. Fig. S6 1H, 13C, COSY, HSQC, and HMBC NMR spectra and low‐ and high‐energy ToF‐MSe spectra of lupinalbin A (4). Fig. S7 1H, 13C, COSY, HSQC, and HMBC NMR spectra and low‐ and high‐energy ToF‐MSe spectra of ayamenin D (14). Fig. S8 1H, 13C, COSY, HSQC, and HMBC NMR spectra, key HMBC correlations, and low‐ and high‐energy ToF‐MSe spectra of 5,7‐dihydroxy‐4′‐methoxycoumaronochromone (lotuschromone, 13). Fig. S9 1H, 13C, COSY, HSQC, and HMBC NMR spectra, key HMBC correlations, and low‐ and high‐energy ToF‐MSe spectra of 4‐hydroxy‐2‐(2′‐hydroxy‐4′‐methoxyphenyl)‐6‐methoxybenzofuran‐3‐carbaldehyde (Lotusaldehyde, 15). Fig. S10 1H, 13C, COSY, HSQC, and HMBC NMR spectra, key HMBC correlations, and low‐ and high‐energy ToF‐MSe spectra of 7‐hydroxy‐3,9‐dimethoxypterocarp‐6a‐ene (Lotuscarpene, 16). Fig. S11 1H, 13C, COSY, HSQC, and HMBC NMR spectra, key HMBC correlations, and low‐ and high‐energy ToF‐MSe spectra of 4,6‐dihydroxy‐2‐(2′‐hydroxy‐4′‐methoxyphenyl)‐3‐(methoxymethyl)benzofuran (25). Fig. S12 1H, 13C, COSY, HSQC, and HMBC NMR spe [file NPH-246-1256-s001.pdf]

New Phytologist

***New Phytologist* Supporting Information**

Article Title: Untargeted Metabolomics Reveals Novel Metabolites in *Lotus japonicus* Roots during Arbuscular Mycorrhiza Symbiosis

Authors: Josef L. Ranner, Georg Stabl, Andrea Piller, Michael Paries, Sapna Sharma, Tian Zeng, Andrea Spaccasassi, Timo D. Stark, Caroline Gutjahr, Corinna Dawid

Article acceptance date: 10 February 2025

The following Supporting Information is available for this article:

Fig. S1: Principal component analysis (PCA) score loading plots of wild-type and mutant samples

Fig. S2: Score loading plots of UPLC-ESI-IM-ToF-MS full scan analysis of *Lotus japonicus* roots harvested at 7 weeks post inoculation (wpi) of mutants *ccamk-3*, *ccamk-13*, *cyclops-3*, *cyclops-4*, *ram1-3*, *ram1-4*, *ram2-1*, and *ram2-2*.

Fig. S3: Score loading plots of UPLC-ESI-IM-ToF-MS full scan analysis of *Lotus japonicus* roots harvested at 10 weeks post inoculation (wpi) of mutants *ccamk-3*, *ccamk-13*, *cyclops-3*, *ram1-3*, *ram1-4*, *ram2-1*, and *ram2-2*.

Fig. S4: S-plots of *Lotus japonicus* root extracts harvested at 7 weeks post inoculation (wpi) comparing mycorrhizal and control samples of mutants *ccamk-3*, *ccamk-13*, *cyclops-3*, *cyclops-4*, *ram1-3*, *ram1-4*, *ram2-1*, and *ram2-2*.

Fig. S5: S-plots of *Lotus japonicus* root extracts harvested at 10 weeks post inoculation (wpi) comparing mycorrhizal and control samples of mutants *ccamk-3*, *ccamk-13*, *cyclops-3*, *ram1-3*, *ram1-4*, *ram2-1*, and *ram2-2*.

Fig. S6: ^{1}H, ^{13}C, COSY, HSQC, and HMBC NMR spectra and low- and high-energy ToF-MS^e spectra of lupinalbin A (**4**)

Fig. S7: ^{1}H, ^{13}C, COSY, HSQC, and HMBC NMR spectra and low- and high-energy ToF-MS^e spectra of ayamenin D (**14**)

Fig. S8: ^{1}H, ^{13}C, COSY, HSQC, and HMBC NMR spectra, key HMBC correlations, and low- and high-energy ToF-MS^{e} spectra of 5,7-dihydroxy-4′-methoxycoumaronochromone (lotuschromone, **13**)

Fig. S9: ^{1}H, ^{13}C, COSY, HSQC, and HMBC NMR spectra, key HMBC correlations, and low- and high-energy ToF-MS^{e} spectra of 4-hydroxy-2-(2′-hydroxy-4′-methoxyphenyl)-6-methoxybenzofuran-3-carbaldehyde (Lotusaldehyde, **15**)

Fig. S10: ^{1}H, ^{13}C, COSY, HSQC, and HMBC NMR spectra, key HMBC correlations, and low- and high-energy ToF-MS^{e} spectra of 7-hydroxy-3,9-dimethoxypterocarp-6a-ene (Lotuscarpene, **16**)

Fig. S11: ^{1}H, ^{13}C, COSY, HSQC, and HMBC NMR spectra, key HMBC correlations, and low- and high-energy ToF-MS^{e} spectra of 4,6-dihydroxy-2-(2′-hydroxy-4′-methoxyphenyl)-3-(methoxymethyl)benzofuran (**25**)

Fig. S12: ^{1}H, ^{13}C, COSY, HSQC, and HMBC NMR spectra, key HMBC correlations, and low- and high-energy ToF-MS^{e} spectra of 4-Hydroxy-2-(2′-hydroxy-4′-methoxyphenyl)-6-methoxy-3-(methoxymethyl)benzofuran (**26**)

Fig. S13: ^{1}H, ^{13}C, COSY, HSQC, and HMBC NMR spectra and low- and high-energy ToF-MS^{e} spectra of a dimeric polyphenol artefact (**29**)

Fig. S14: Structure proposals for the dimeric polyphenol artefact **29**

Fig. S15: Low- and high-energy ToF-MS^{e} spectra of postulated 4-hydroxy-2-(2′-hydroxy-4′-methoxyphenyl)-6-methoxy-3-(ethoxymethyl)benzofuran (**27**)

Fig. S16: Normalized response of **1**, **9**, **10**, **15**, **16**, and **25-30** in MeOH, EtOH and MeCN after 0, 24, 48, and 72 h of UV light exposure

Fig. S17: Low- and high-energy ToF-MS^{e} spectra of postulated 7,9-dihydroxy-3-methoxypterocarp-6a-ene (**28**)

Fig. S18: Low- and high-energy ToF-MS^{e} spectra of postulated dimeric polyphenol **30**, a precursor to isolated artefact **29**

Fig. S19: Low- and high-energy ToF-MS^{e} spectra of a postulated hydroxy dimethoxypterocarp-6a-ene (**1**)

Fig. S20: Low- and high-energy ToF-MS^{e} spectra of a postulated dihydroxy trimethoxy aryl benzofuran-3-carbaldehyde (**9**)

Fig. S21: Low- and high-energy ToF-MS^{e} spectra of a postulated hydroxy trimethoxypterocarp-6a-ene (**10**)

Fig. S22: Low- and high-energy ToF-MS^e spectra of a postulated 7-hydroxy-3,9-dimethoxycoumestan (**11**)

Fig. S23: Mean normalized abundance of compounds **1**, **3** (lupinalbin B), **13** (lotuschromone), **15** (lotusaldehyde), and **16** (lotuscarpene) in control and AM wild-type and mutant roots harvested at 7 wpi

Fig. S24: Mean normalized abundance of unidentified features *m/z* 681.1050 and *m/z* 710.1082 in control and AM wild-type and mutant roots harvested at 7 wpi

Fig. S25: Low- and high-energy ToF-MS^e spectra of compound **2**

Fig. S26: Low- and high-energy ToF-MS^e spectra of compound **5**

Fig. S27: Low- and high-energy ToF-MS^e spectra of compound **6**

Fig. S28: Low- and high-energy ToF-MS^e spectra of compound **17**

Fig. S29: Low- and high-energy ToF-MS^e spectra of compound **18**

Fig. S30: Low- and high-energy ToF-MS^e spectra of compound **19**

Table S1: Chromatographic conditions for compound isolation

Table S2: Colonization data including intraradical hyphae, arbuscules, vesicles, total root length colonization and mean colonization per genotype (cf. xlsx-file)

Table S3: Potential marker candidates in *Lotus japonicus* wild-type and mutant roots (cf. xlsx-file)

Table S4: Metabolites included in the in-house *in silico* database (cf. xlsx-file)

Table S5: *In silico* database hits for mass spectral features in 7 wpi root samples (cf. xlsx-file)

Table S6: *In silico* database hits for mass spectral features in 10 wpi root samples (cf. xlsx-file)

Table S7: ToF-MS^e and NMR data of isolated compounds

Table S8: Annotated transcriptome data of *Lotus japonicus* AM and control root samples (cf. xlsx-file)

Table S9: Significantly AM-induced polyphenol biosynthesis genes (cf. xlsx-file)

Methods S1: Marker compound isolation

Methods S2: Omics correlation analysis

Notes S1: Analysis of NMR Spectra of 5,7-dihydroxy-4′-methoxycoumaronochromone (lotuschromone, **13**)

Notes S2: Analysis of NMR Spectra of 4-hydroxy-2-(2′-hydroxy-4′-methoxyphenyl)-6-methoxybenzofuran-3-carbaldehyde (lotusaldehyde, **15**)

Notes S3: Analysis of NMR Spectra of 7-hydroxy-3,9-dimethoxypterocarp-6a-ene (lotuscarpene, **16**)

Notes S4: Analysis of NMR Spectra of the dimeric polyphenol artefact **29**

Fig. S1: Principal component analysis (PCA) score loading plots (PC1 vs. PC2; ANOVA *p*-value ≤0.05; fold change ≥2) of ultraperformance liquid chromatography–electrospray ionization–ion mobility–time-of-flight–mass spectrometry (UPLC-ESI-IM-ToF-MS) full scan analysis (50–1200 Da, ESI^{-}, sensitivity mode) of **a)** root extracts of *Lotus japonicus* wild-type Gifu (WT), eight mutants (*ccamk-3*, *ccamk-13*, *cyclops-3*, *cyclops-4*, *ram1-3*, *ram1-4*, *ram2-1*, and *ram2-2*) harvested at 7 weeks post inoculation (wpi), and quality control (QC) pools; **b)** root extracts of wild type and the eight mutants harvested at 10 wpi, and QC pools; **c)** *L. japonicus* wild-type root extracts harvested at 7 wpi; and **d)** wild-type root extracts harvested at 10 wpi. One (only *cyclops-4*, 10 wpi) to four biological replicates and three technical replicates per biological replicate were analyzed. Percentage numbers indicate the total root length colonization of the corresponding biological replicate. Non-mycorrhizal control roots are highlighted in shades of blue, and arbuscular mycorrhizal (AM) roots are highlighted in shades of brown, with allelic mutants colored in the same shade.

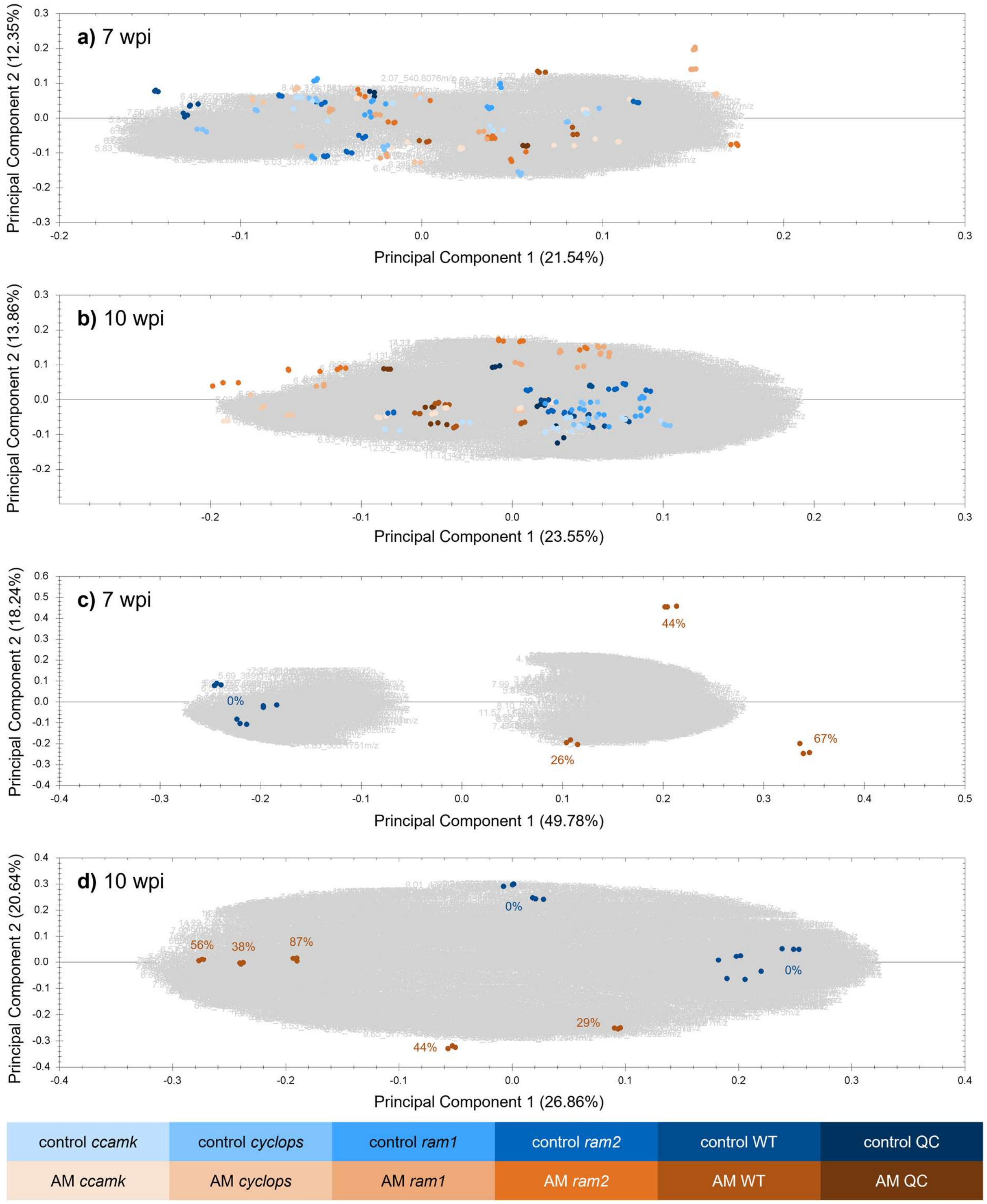
a) 7 wpi
Principal Component 1 (21.54%)
Principal Component 2 (12.35%)
b) 10 wpi
Principal Component 1 (23.55%)
Principal Component 2 (13.86%)
c) 7 wpi
0%
44%
26%
67%
Principal Component 1 (49.78%)
Principal Component 2 (18.24%)
d) 10 wpi
0%
56%
38%
87%
0%
29%
44%
Principal Component 1 (26.86%)
Principal Component 2 (20.64%)
control ccamk
control cyclops
control ram1
control ram2
control WT
control QC
AM ccamk
AM cyclops
AM ram1
AM ram2
AM WT
AM QC

Fig. S2: Score loadings plots (PC1 vs. PC2; ANOVA *p*-value ≤0.05; fold change ≥2) of ultraperformance liquid chromatography–electrospray ionization–ion mobility–time-of-flight–mass spectrometry (UPLC-ESI-IM-ToF-MS) full scan analysis (50–1200 Da, ESI⁻, sensitivity mode) of *Lotus japonicus* roots harvested at 7 weeks post inoculation (wpi) of mutants **a)** *ccamk-3*, **b)** *ccamk-13*, **c)** *cyclops-3*, **d)** *cyclops-4*, **e)** *ram1-3*, **f)** *ram1-4*, **g)** *ram2-1*, and **h)** *ram2-2*, with control samples highlighted in blue and mycorrhizal (AM) samples highlighted in purple. Three technical replicates were used per biological replicate.

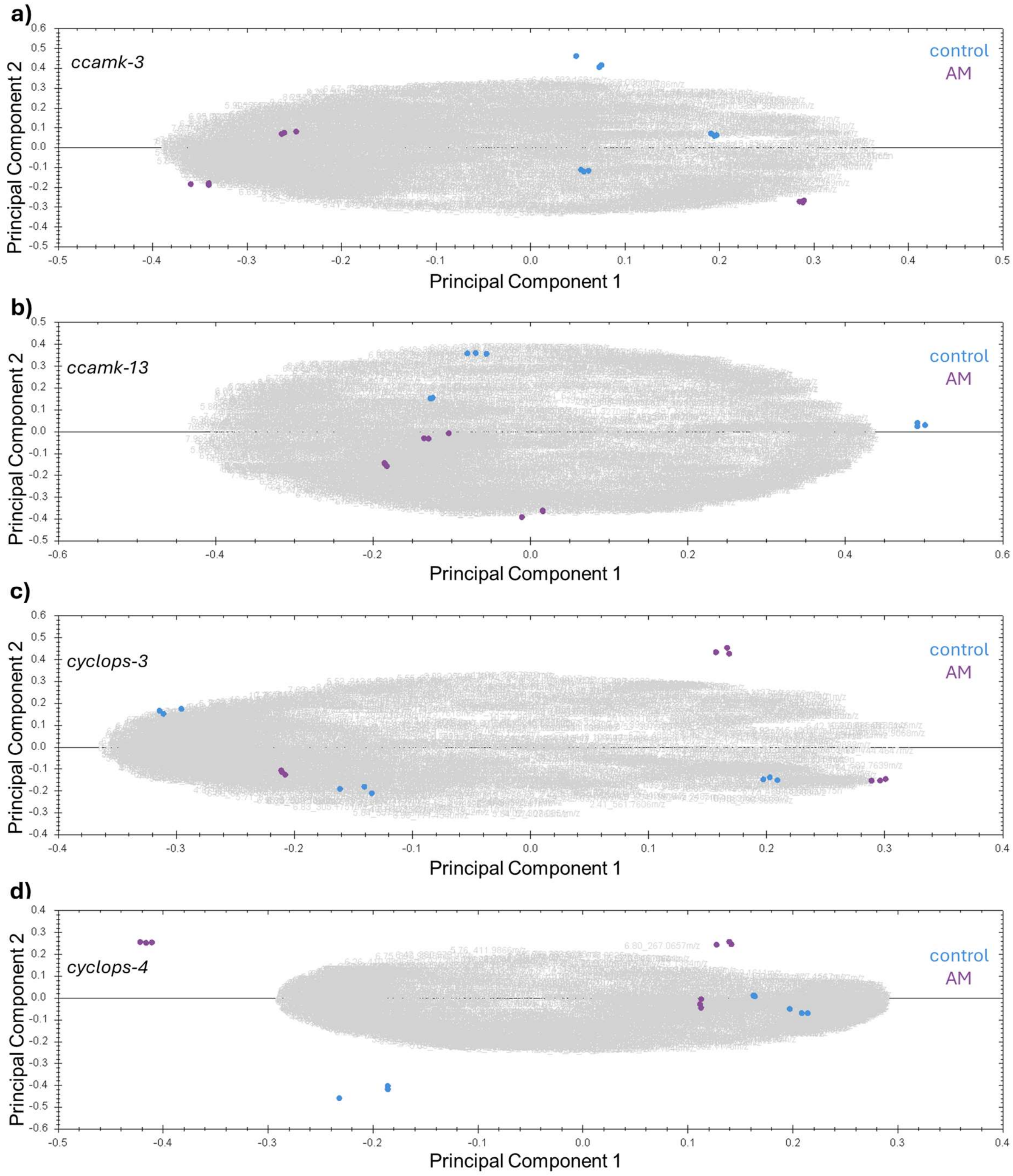

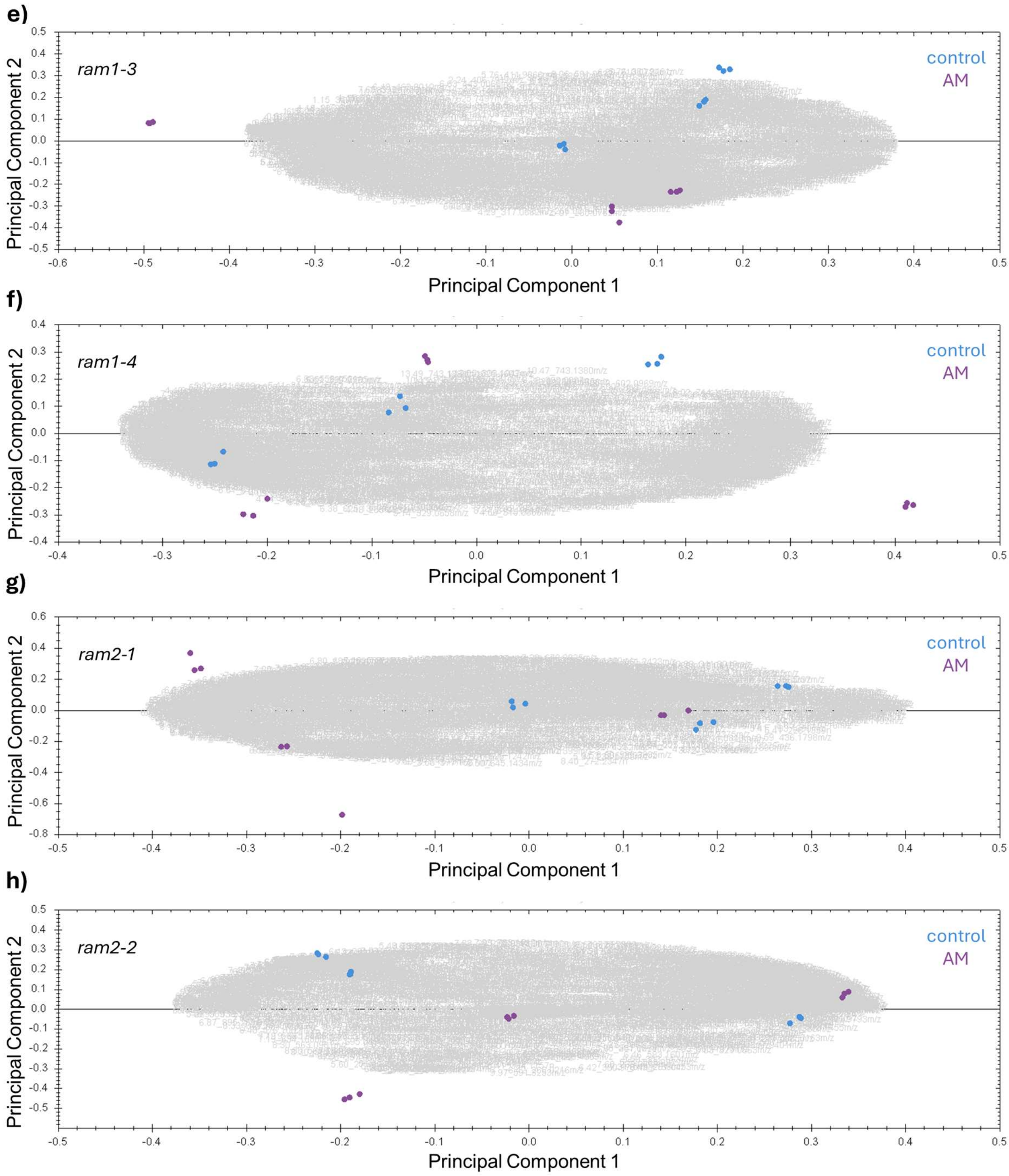
e)
ram1-3
control
AM
Principal Component 1
Principal Component 2
f)
ram1-4
control
AM
Principal Component 1
Principal Component 2
g)
ram2-1
control
AM
Principal Component 1
Principal Component 2
h)
ram2-2
control
AM
Principal Component 1
Principal Component 2

Fig. S3: Score loadings plots (PC1 vs. PC2; ANOVA *p*-value ≤0.05; fold change ≥2) of ultraperformance liquid chromatography–electrospray ionization–ion mobility–time-of-flight–mass spectrometry (UPLC-ESI-IM-ToF-MS) full scan analysis (50–1200 Da, ESI^{-}, sensitivity mode) of *Lotus japonicus* roots harvested at 10 weeks post inoculation (wpi) of mutants **a)** *ccamk-3*, **b)** *ccamk-13*, **c)** *cyclops-3*, **d)** *ram1-3*, **e)** *ram1-4*, **f)** *ram2-1*, and **g)** *ram2-2*, with control samples highlighted in blue and mycorrhizal (AM) samples highlighted in purple. Three technical replicates were used per biological replicate. *Note:* Only one biological replicate of *cyclops-4* was available per condition, therefore, it has not been evaluated.

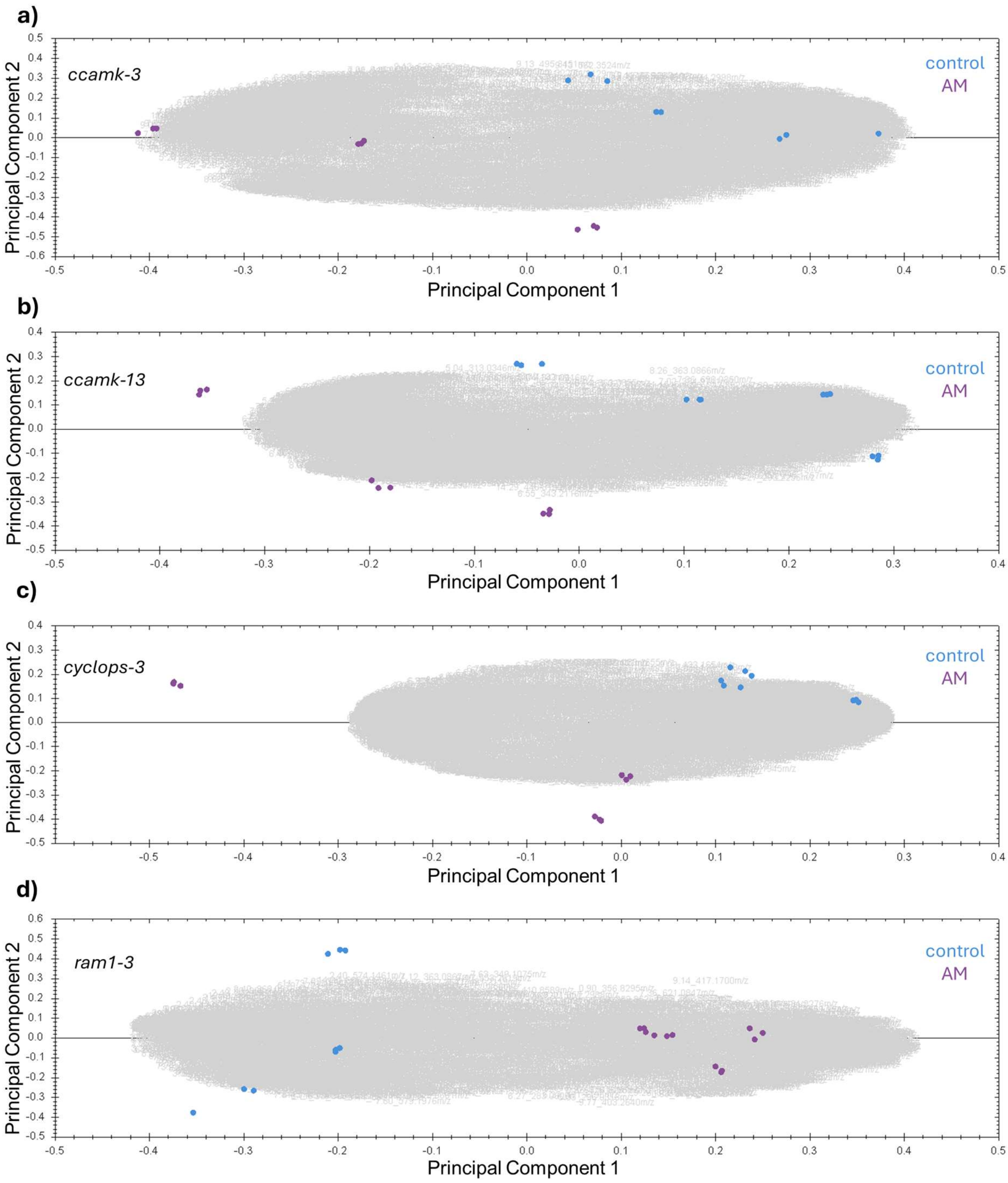

e)

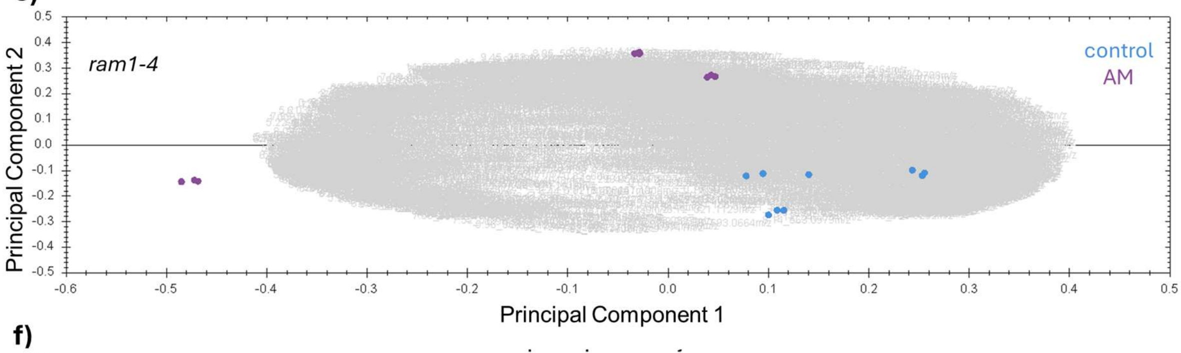
ram1-4
control
AM
Principal Component 1
Principal Component 2
0.5
0.4
0.3
0.2
0.1
0.0
-0.1
-0.2
-0.3
-0.4
-0.5
-0.6
-0.5
-0.4
-0.3
-0.2
-0.1
0.0
0.1
0.2
0.3
0.4
0.5

f)

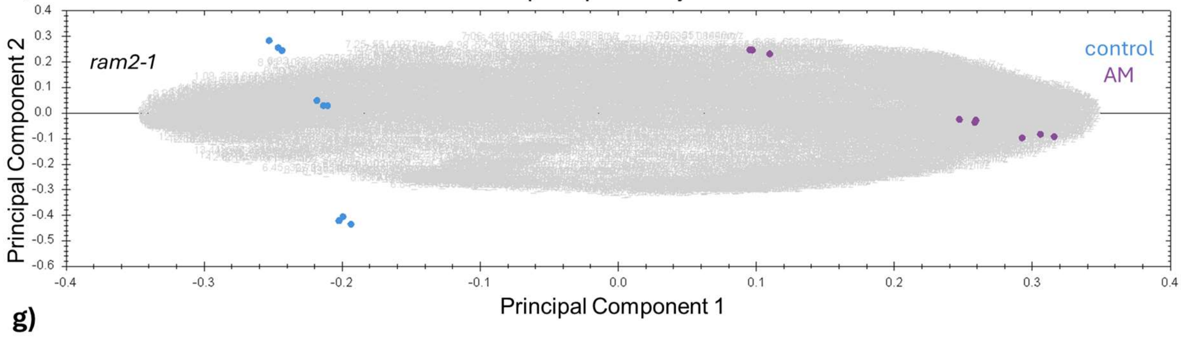
ram2-1
control
AM
Principal Component 1
Principal Component 2
0.4
0.3
0.2
0.1
0.0
-0.1
-0.2
-0.3
-0.4
-0.5
-0.6
-0.4
-0.3
-0.2
-0.1
0.0
0.1
0.2
0.3
0.4

g)

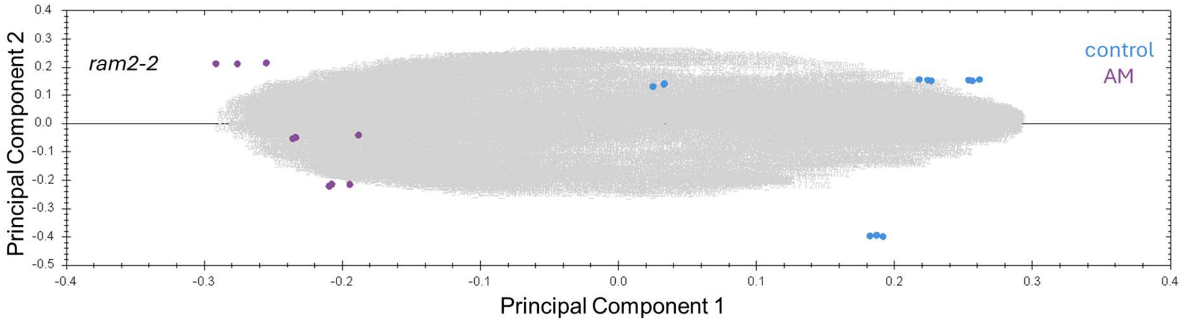
ram2-2
control
AM
Principal Component 1
Principal Component 2
0.4
0.3
0.2
0.1
0.0
-0.1
-0.2
-0.3
-0.4
-0.5
-0.4
-0.3
-0.2
-0.1
0.0
0.1
0.2
0.3
0.4

Fig. S4: S-plots of *Lotus japonicus* root extracts harvested at 7 weeks post inoculation (wpi) comparing mycorrhizal (AM, +1) and control (−1) samples of mutants **a)** *ccamk-3*, **b)** *ccamk-13*, **c)** *cyclops-3*, **d)** *cyclops-4*, **e)** *ram1-3*, **f)** *ram1-4*, **g)** *ram2-1*, and **h)** *ram2-2* with highlighted marker compounds (blue: identified compounds, orange: postulated compounds).

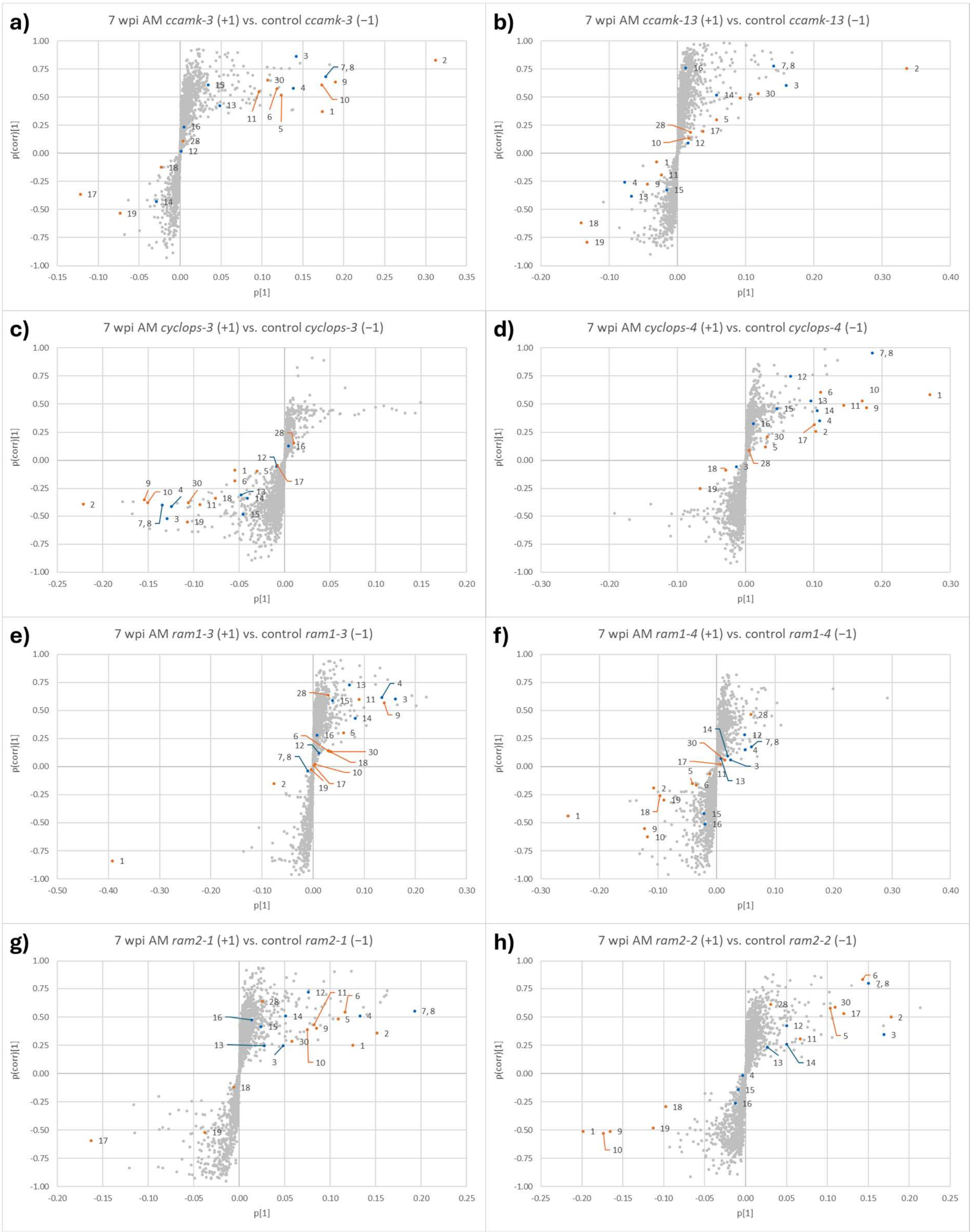

Fig. S5: S-plots of *Lotus japonicus* root extracts harvested at 10 weeks post inoculation (wpi) comparing mycorrhizal (AM, +1) and control (−1) samples of mutants **a)** *ccamk-3*, **b)** *ccamk-13*, **c)** *cyclops-3*, **d)** *ram1-3*, **e)** *ram1-4*, **f)** *ram2-1*, and **g)** *ram2-2* with highlighted marker compounds (blue: identified compounds, orange: postulated compounds).

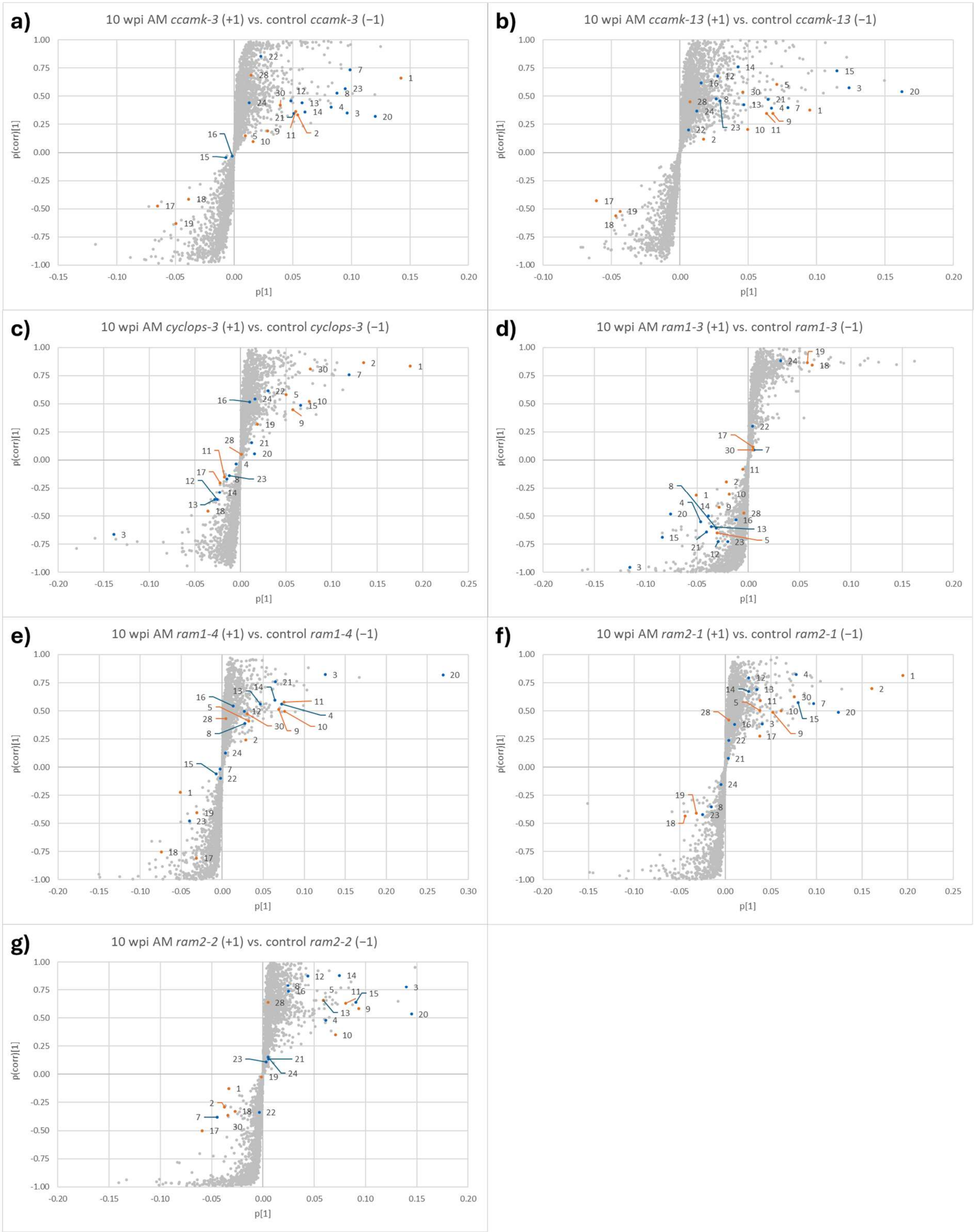

Fig. S6: ^{1}H (**a**), ^{13}C (**b**), COSY (**c**), HSQC (**d**), and HMBC (**e**) NMR spectra (CD_3CN, 600/151 MHz, 300 K) and low- (**f**) and high-energy (**g**) ToF-MS^{e} spectra of lupinalbin A (**4**). COSY: Correlation Spectroscopy; HSQC: Heteronuclear Single Quantum Coherence Spectroscopy; HMBC: Heteronuclear Multiple Bond Correlation Spectroscopy; NMR: Nuclear Magnetic Resonance Spectroscopy; CD_3CN: acetonitrile-d$_3$; ToF-MS^{e}: Time-of-flight-Mass Spectrometry with elevated energy.

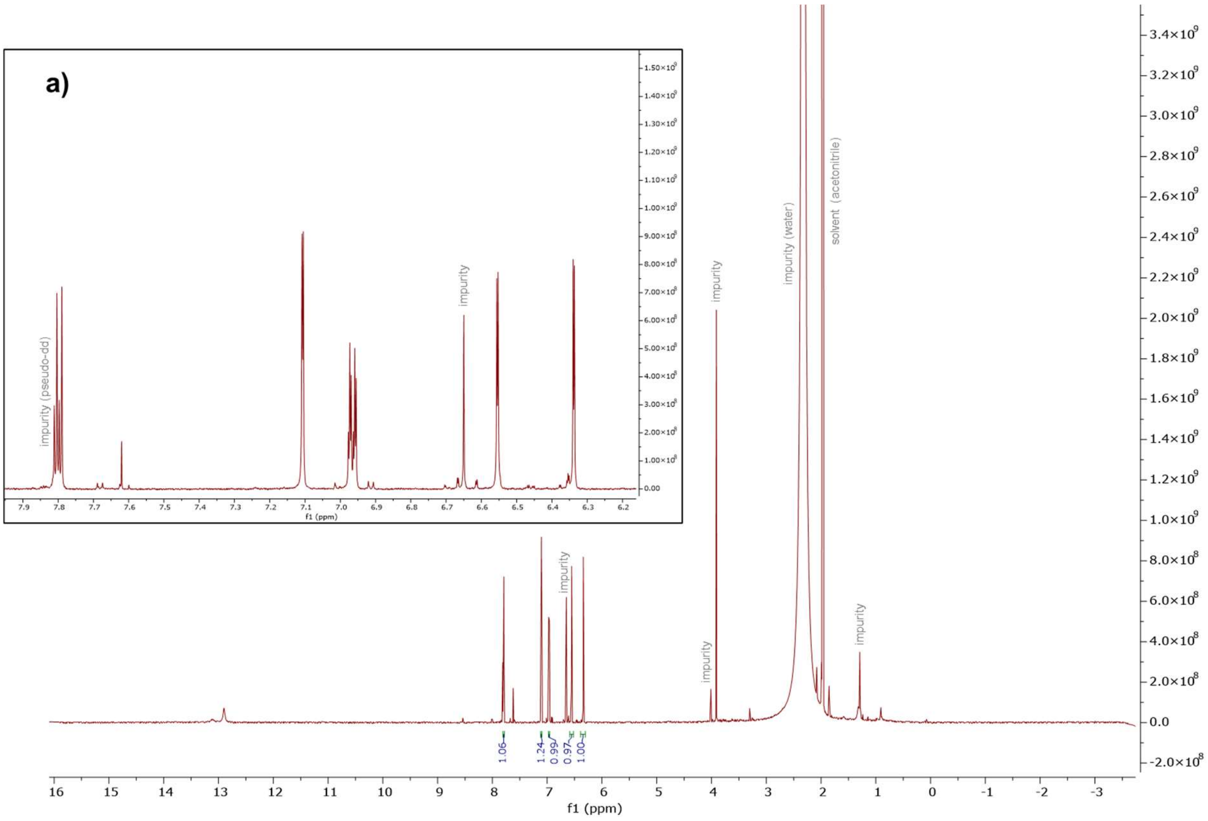

b)

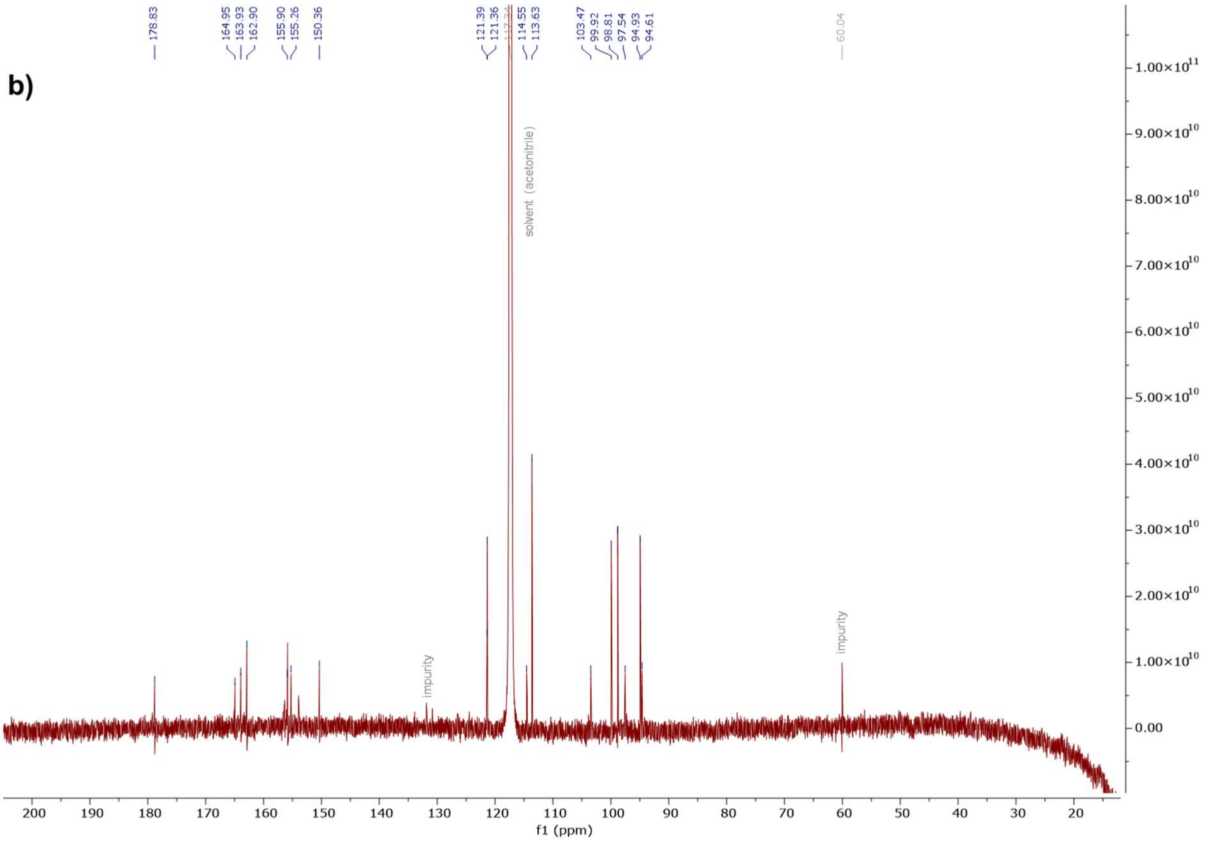
178.83
164.95
163.93
162.90
155.90
155.26
150.36
121.39
121.36
114.55
113.63
103.47
99.92
98.81
97.54
94.93
94.61
60.04
solvent (acetonitrile)
impurity
impurity
f1 (ppm)

c)

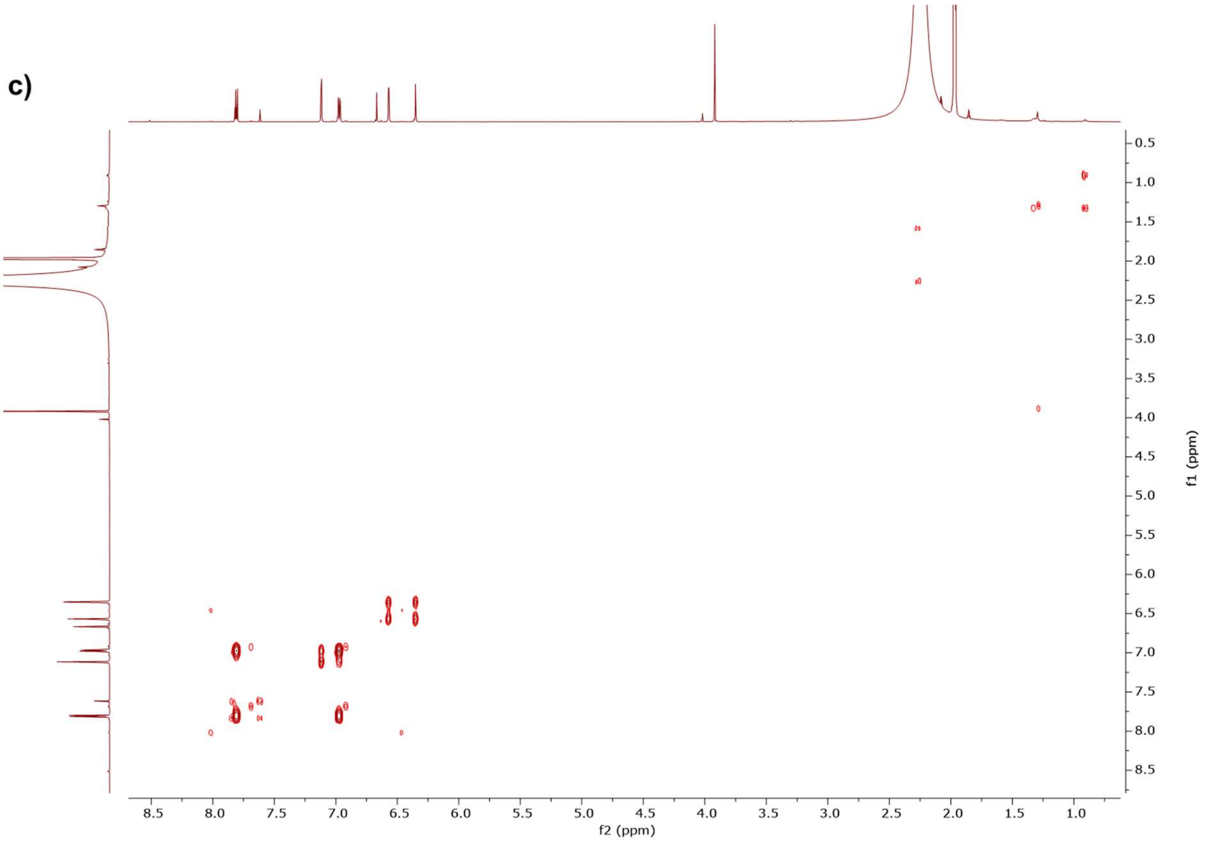
f1 (ppm)
f2 (ppm)

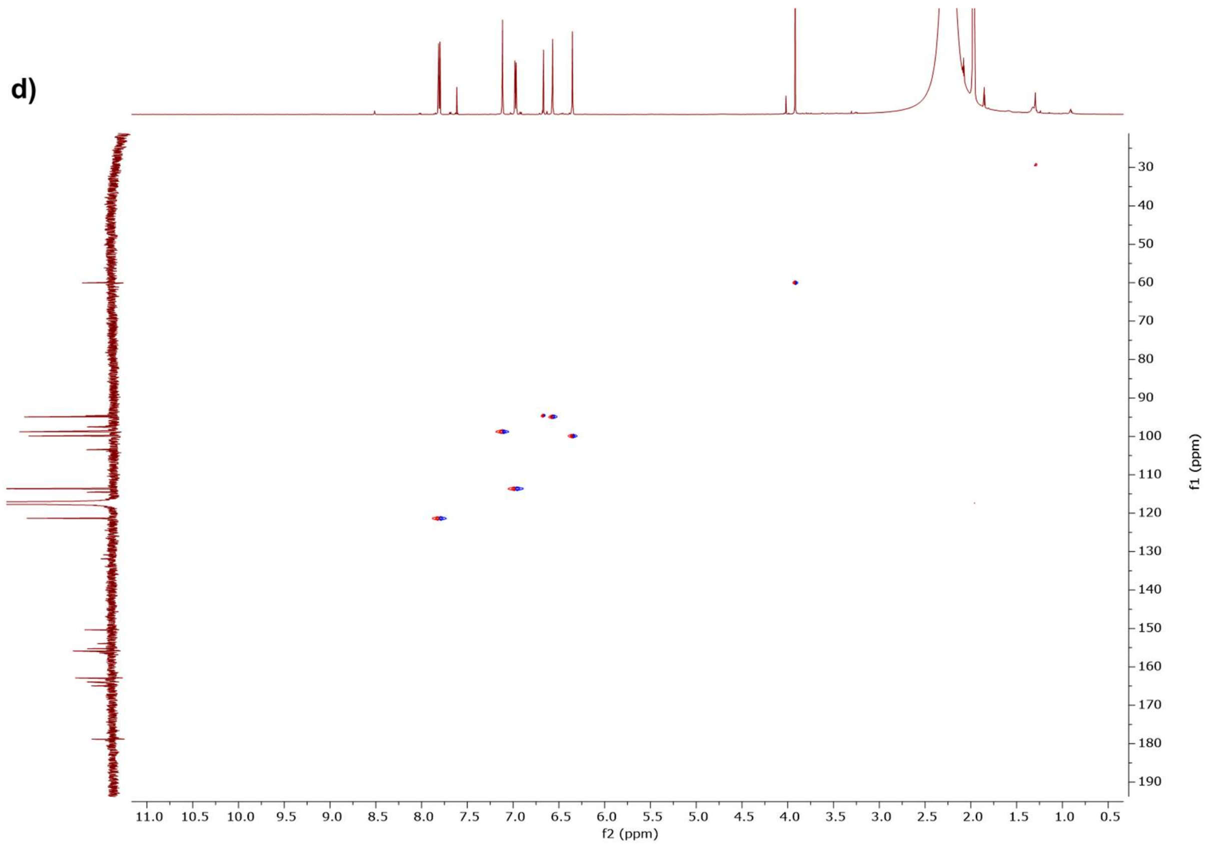
d)
f2 (ppm)
f1 (ppm)

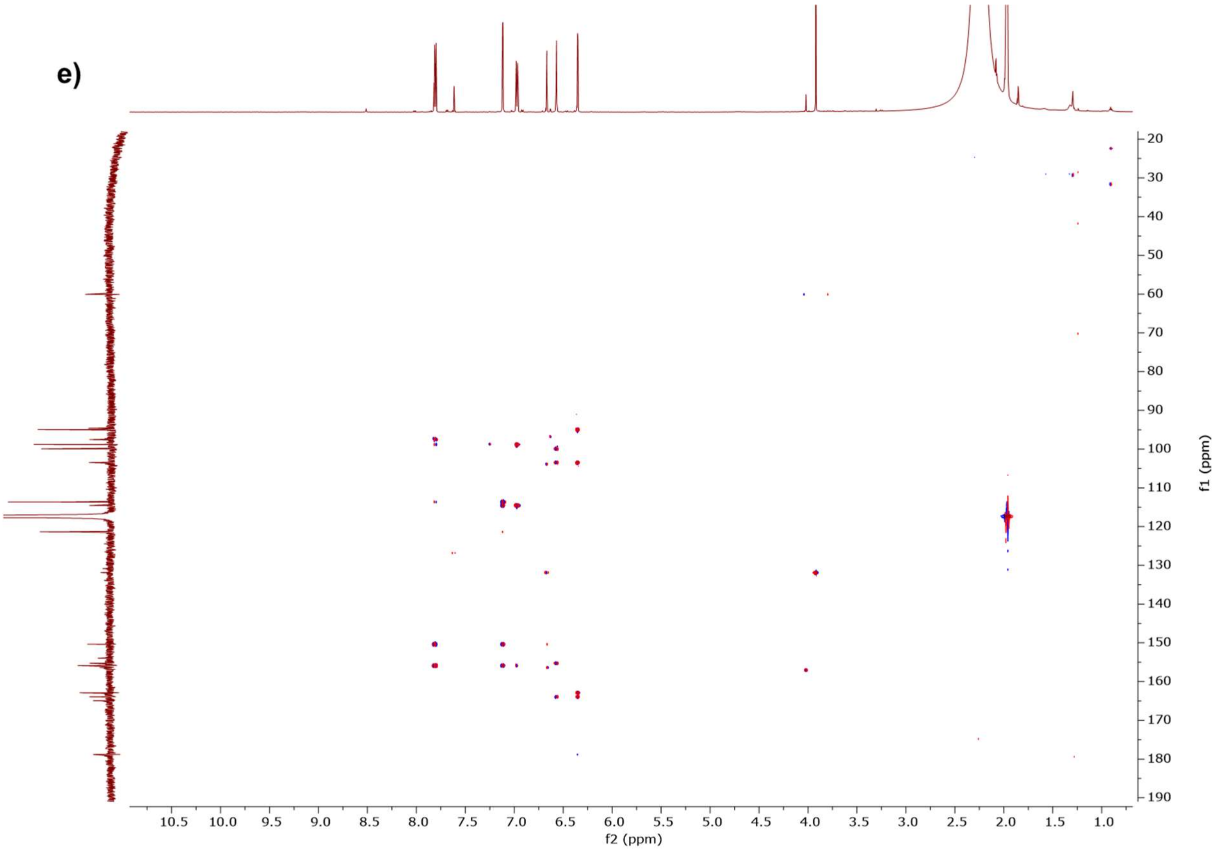
e)
f2 (ppm)
f1 (ppm)

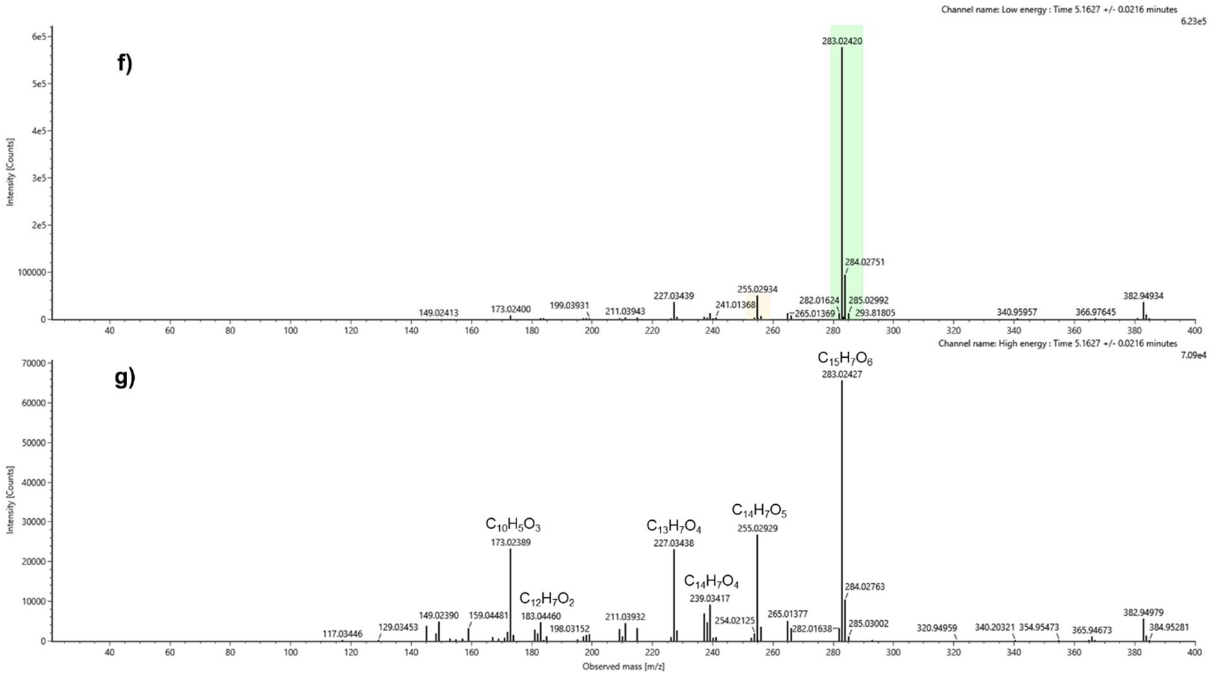

f)
Channel name: Low energy : Time 5.1627 +/- 0.0216 minutes
6.23e5
283.02420
284.02751
255.02934
227.03439
241.01368
282.01624
285.02992
265.01369
293.81805
149.02413
173.02400
199.03931
211.03943
340.95957
366.97645
382.94934
Intensity [Counts]
g)
Channel name: High energy : Time 5.1627 +/- 0.0216 minutes
7.09e4
$C_{15}H_7O_6$
283.02427
$C_{10}H_5O_3$
173.02389
$C_{13}H_7O_4$
227.03438
$C_{14}H_7O_5$
255.02929
$C_{14}H_7O_4$
239.03417
$C_{12}H_7O_2$
183.04460
284.02763
265.01377
254.02125
282.01638
285.03002
211.03932
198.03152
159.04481
149.02390
129.03453
117.03446
320.94959
340.20321
354.95473
365.94673
382.94979
384.95281
Intensity [Counts]
Observed mass [m/z]

Fig. S7: ^{1}H (**a**), ^{13}C (**b**), COSY (**c**), HSQC (**d**), and HMBC (**e**) NMR spectra (CD_3CN, 600/151 MHz, 300 K) and low- (**f**) and high-energy (**g**) ToF-MS^{e} spectra of ayamenin D (**14**). COSY: Correlation Spectroscopy; HSQC: Heteronuclear Single Quantum Coherence Spectroscopy; HMBC: Heteronuclear Multiple Bond Correlation Spectroscopy; NMR: Nuclear Magnetic Resonance Spectroscopy; CD_3CN: acetonitrile-d$_3$; ToF-MS^{e}: Time-of-flight-Mass Spectrometry with elevated energy.

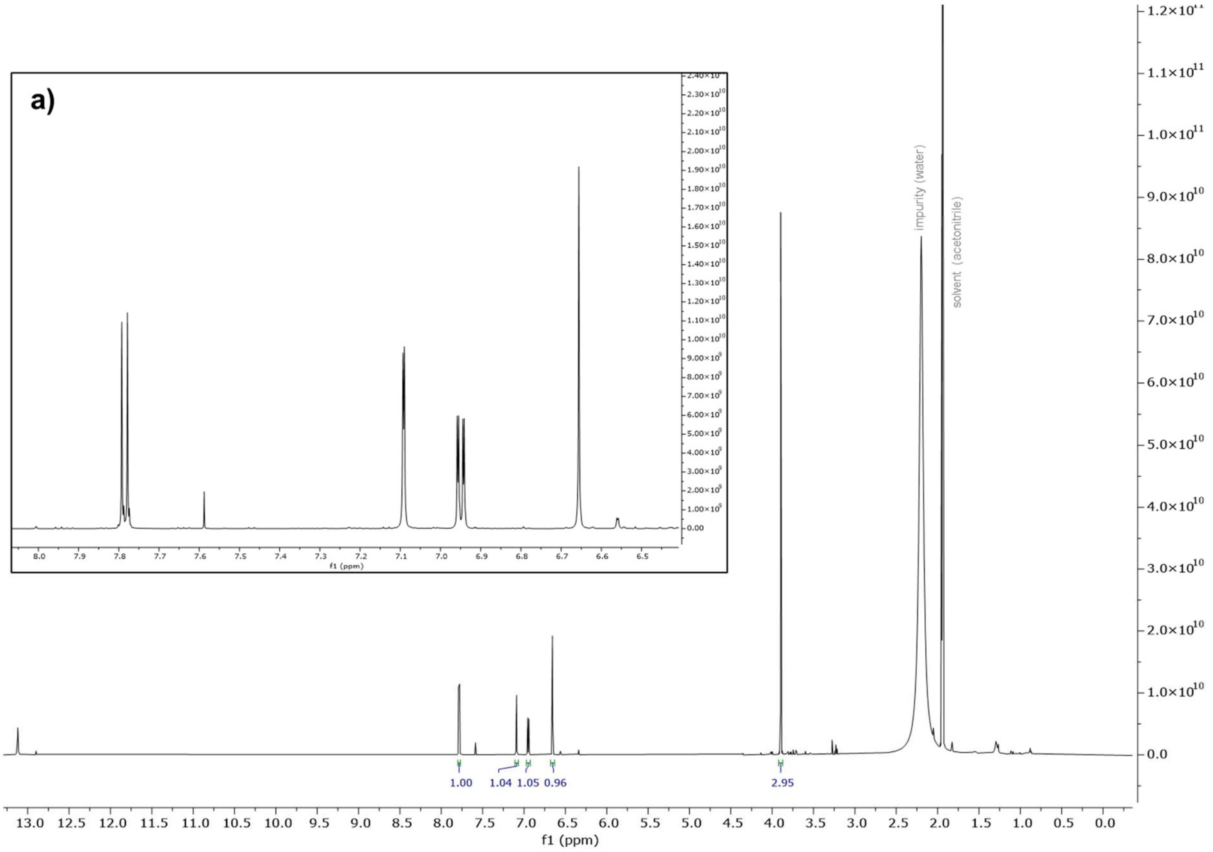

b)

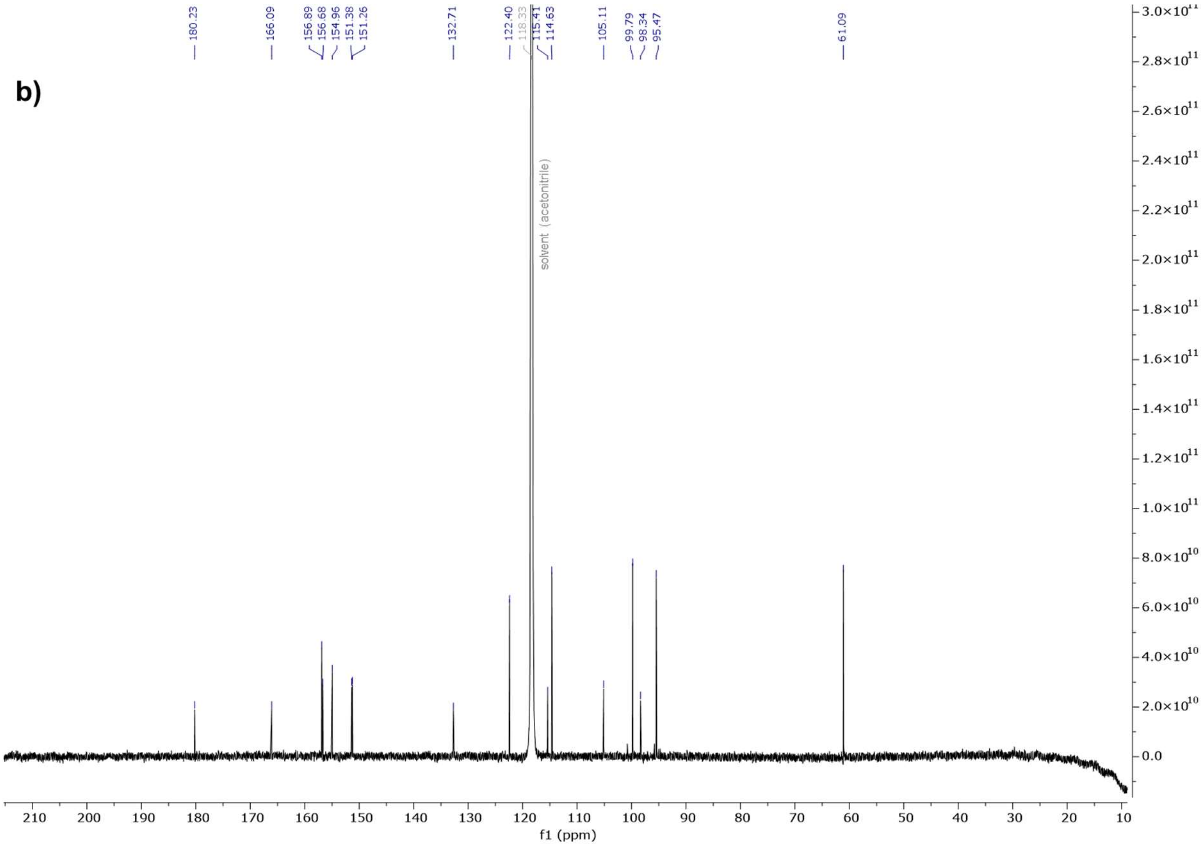
180.23
166.09
156.89
156.68
154.96
151.38
151.26
132.71
122.40
118.33
115.41
114.63
105.11
99.79
98.34
95.47
61.09
solvent (acetonitrile)
f1 (ppm)

c)

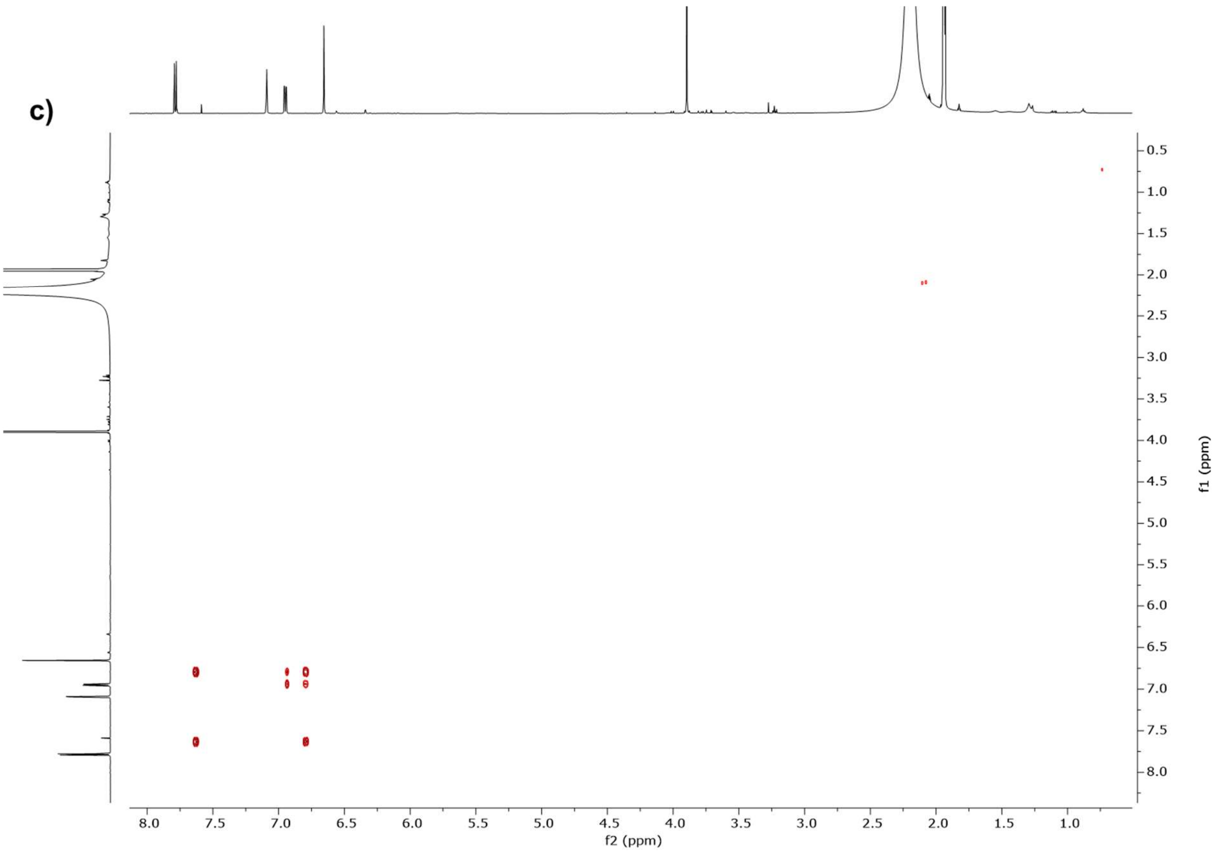
f2 (ppm)
f1 (ppm)

d)

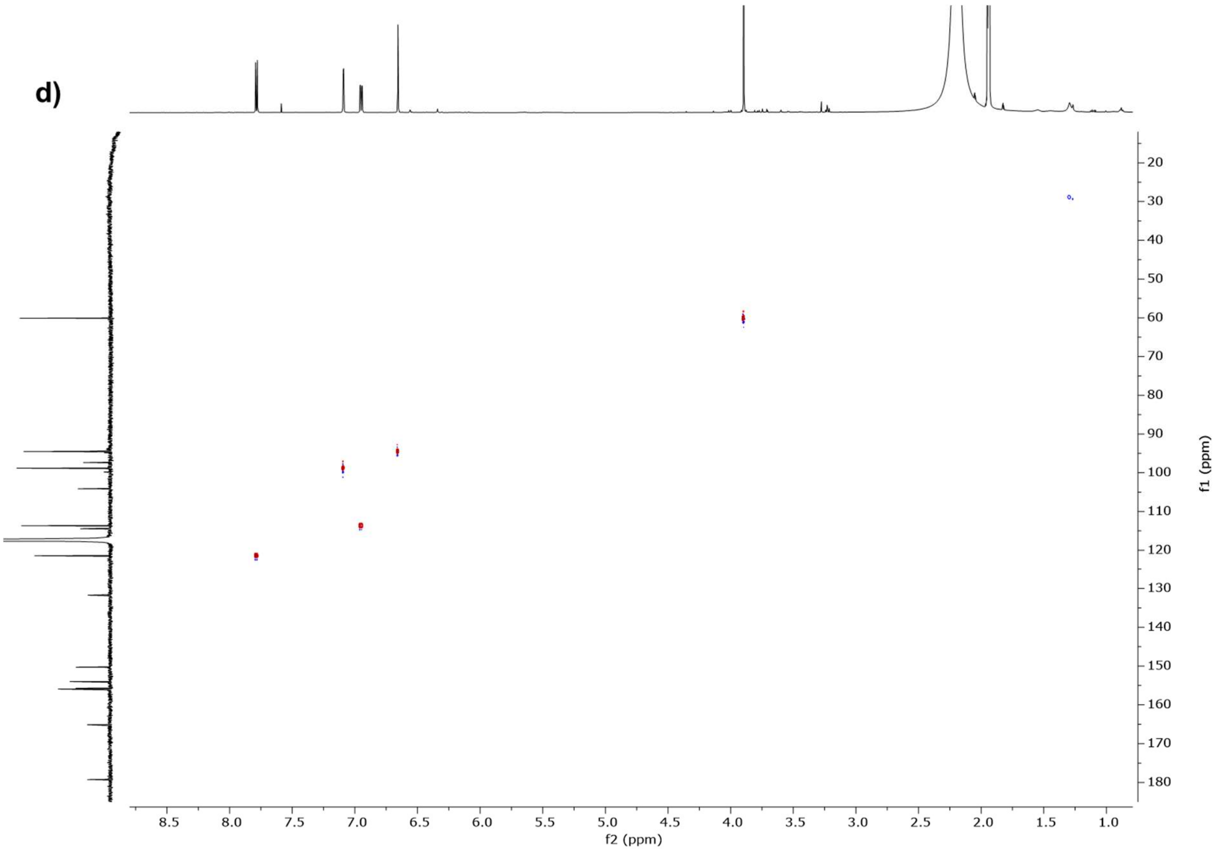

e)

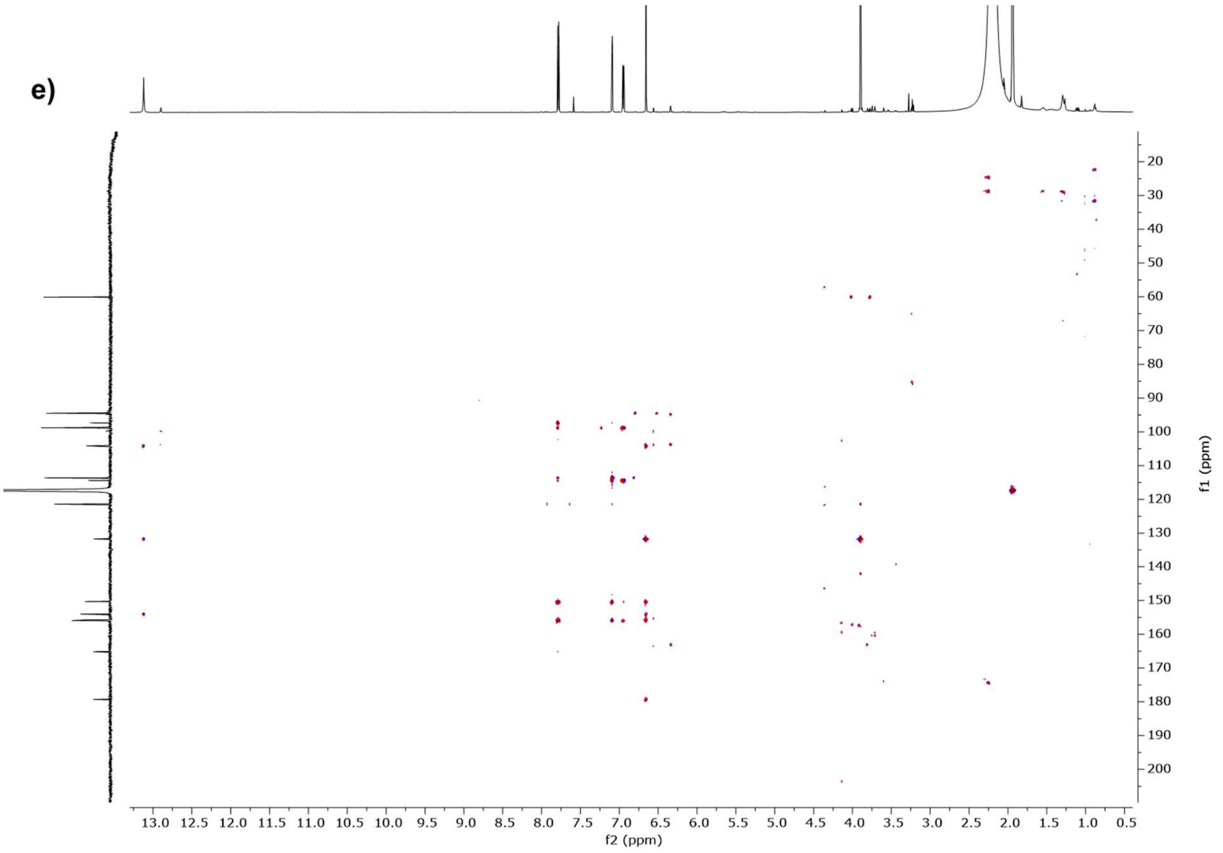

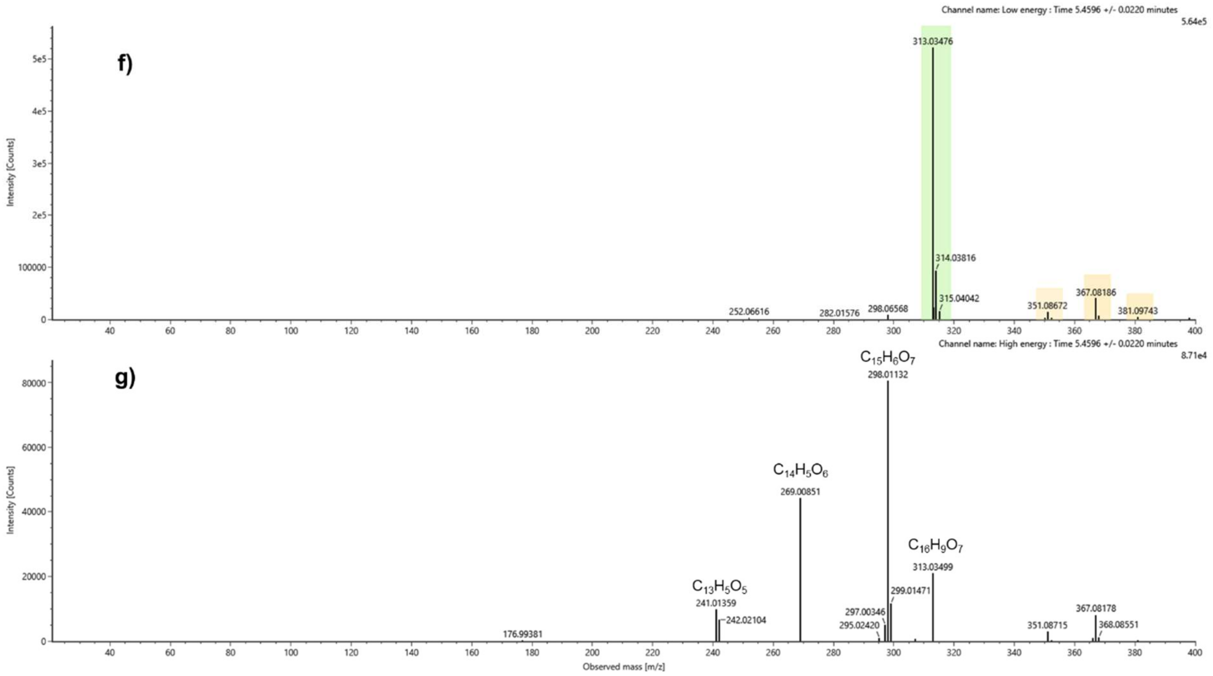
Channel name: Low energy : Time 5.4596 +/- 0.0220 minutes
5.64e5
f)
313.03476
314.03816
315.04042
252.06616
282.01576
298.06568
351.08672
367.08186
381.09743
Intensity [Counts]
Channel name: High energy : Time 5.4596 +/- 0.0220 minutes
8.71e4
g)
$C_{15}H_6O_7$
298.01132
$C_{14}H_5O_6$
269.00851
$C_{16}H_9O_7$
313.03499
$C_{13}H_5O_5$
241.01359
242.02104
297.00346
295.02420
299.01471
176.99381
351.08715
367.08178
368.08551
Intensity [Counts]
Observed mass [m/z]

Fig. S8: ^{1}H (**a**), ^{13}C (**b**), COSY (**c**), HSQC (**d**), and HMBC (**e**) NMR spectra (CD_3CN, 950/239 MHz, 300 K) with key HMBC correlations (**f**) and low- (**g**) and high-energy (**h**) ToF-MS^e spectra of 5,7-dihydroxy-4′-methoxycoumaronochromone (lotuschromone, **13**). COSY: Correlation Spectroscopy; HSQC: Heteronuclear Single Quantum Coherence Spectroscopy; HMBC: Heteronuclear Multiple Bond Correlation Spectroscopy; NMR: Nuclear Magnetic Resonance Spectroscopy; CD_3CN: acetonitrile-d_3; ToF-MS^e: Time-of-flight-Mass Spectrometry with elevated energy.

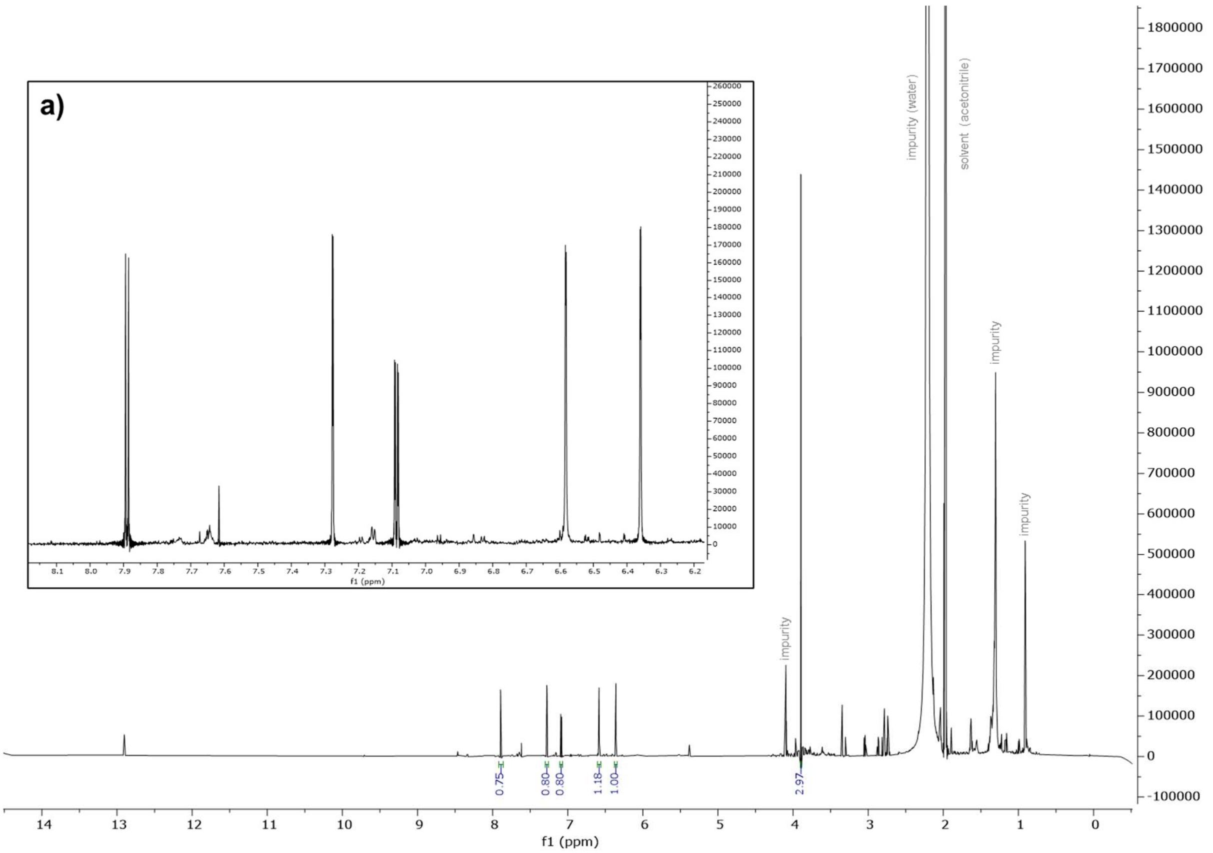

b)

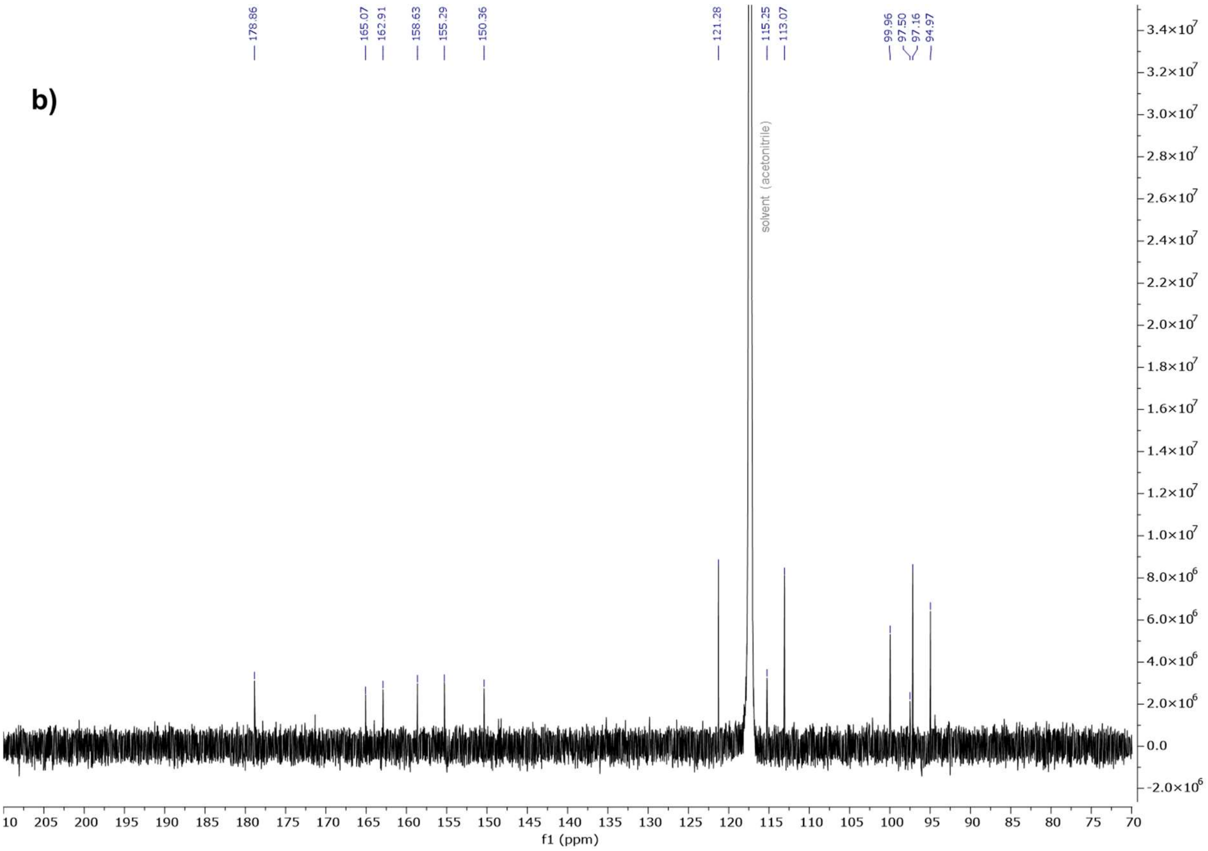
178.86
165.07
162.91
158.63
155.29
150.36
121.28
115.25
113.07
99.96
97.50
97.16
94.97
solvent (acetonitrile)
f1 (ppm)

c)

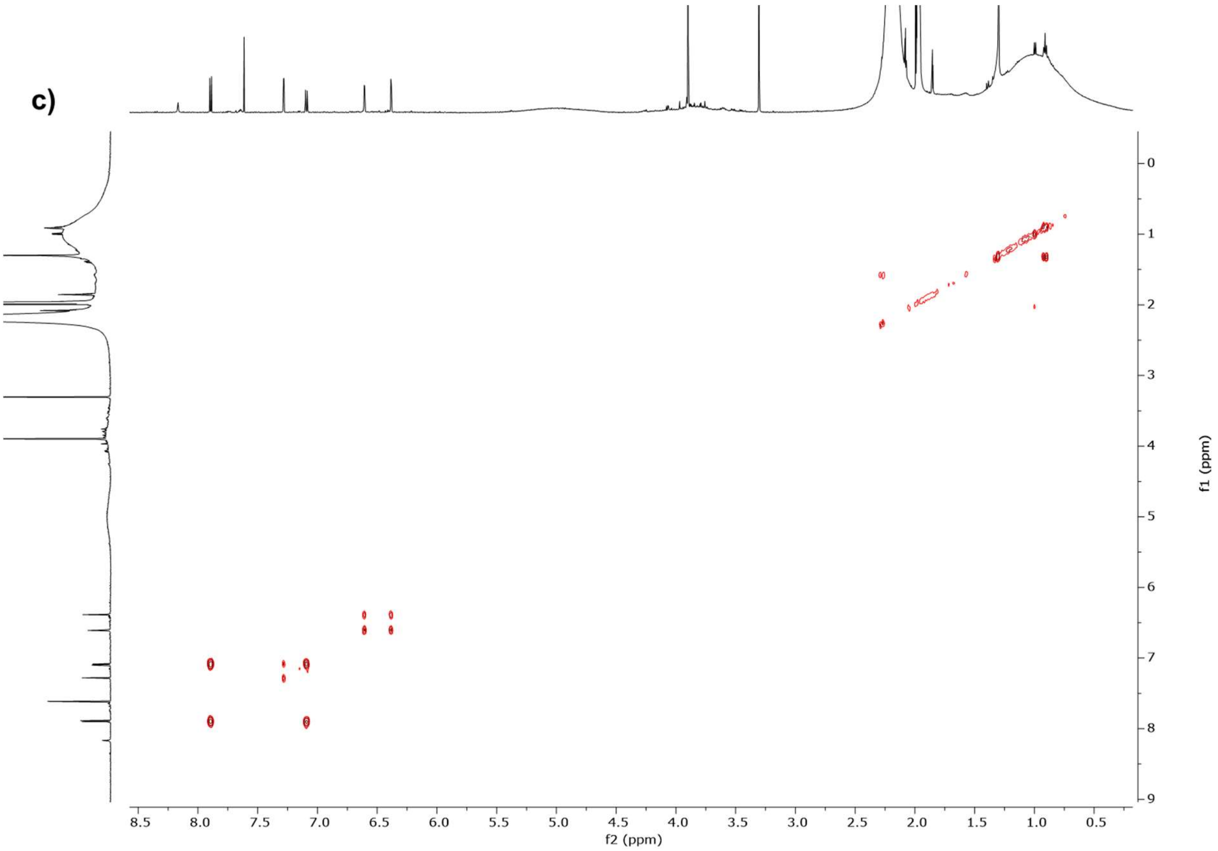
f2 (ppm)
f1 (ppm)

d)

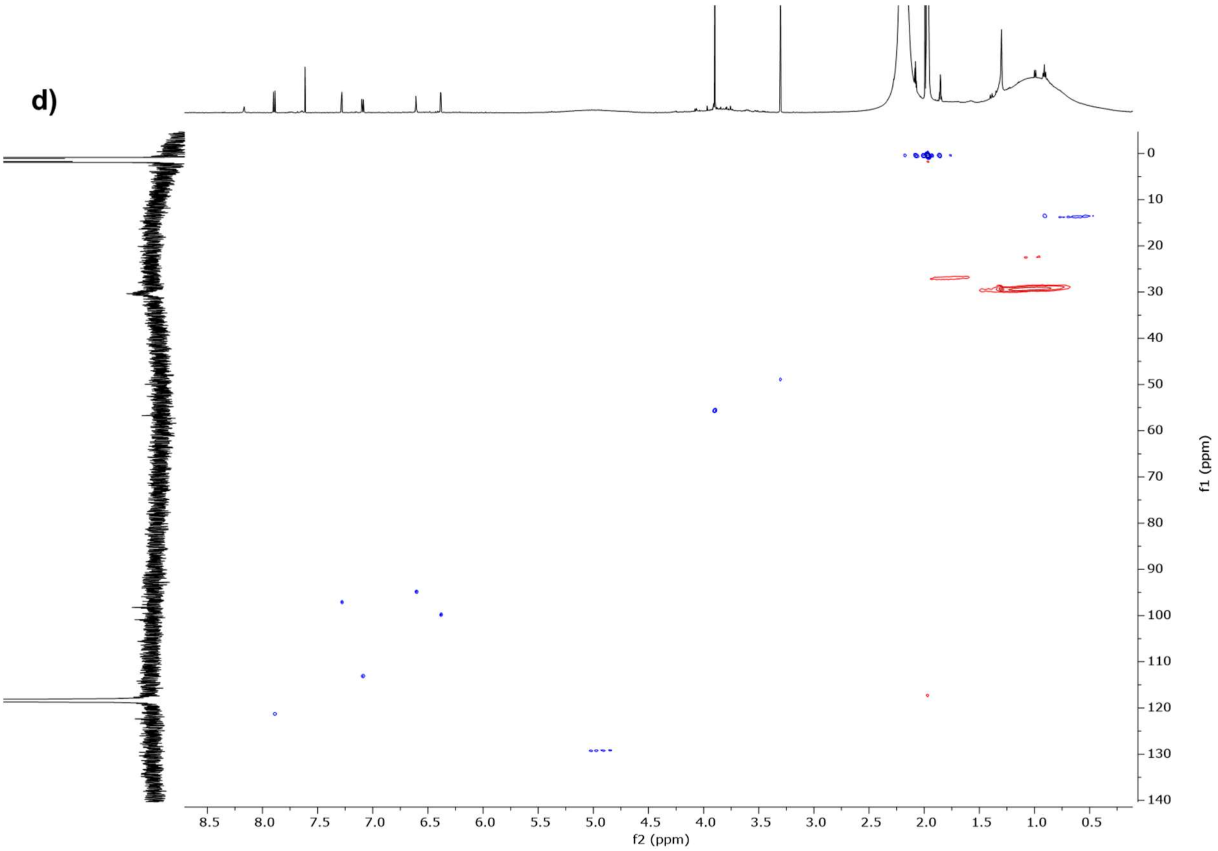

e)

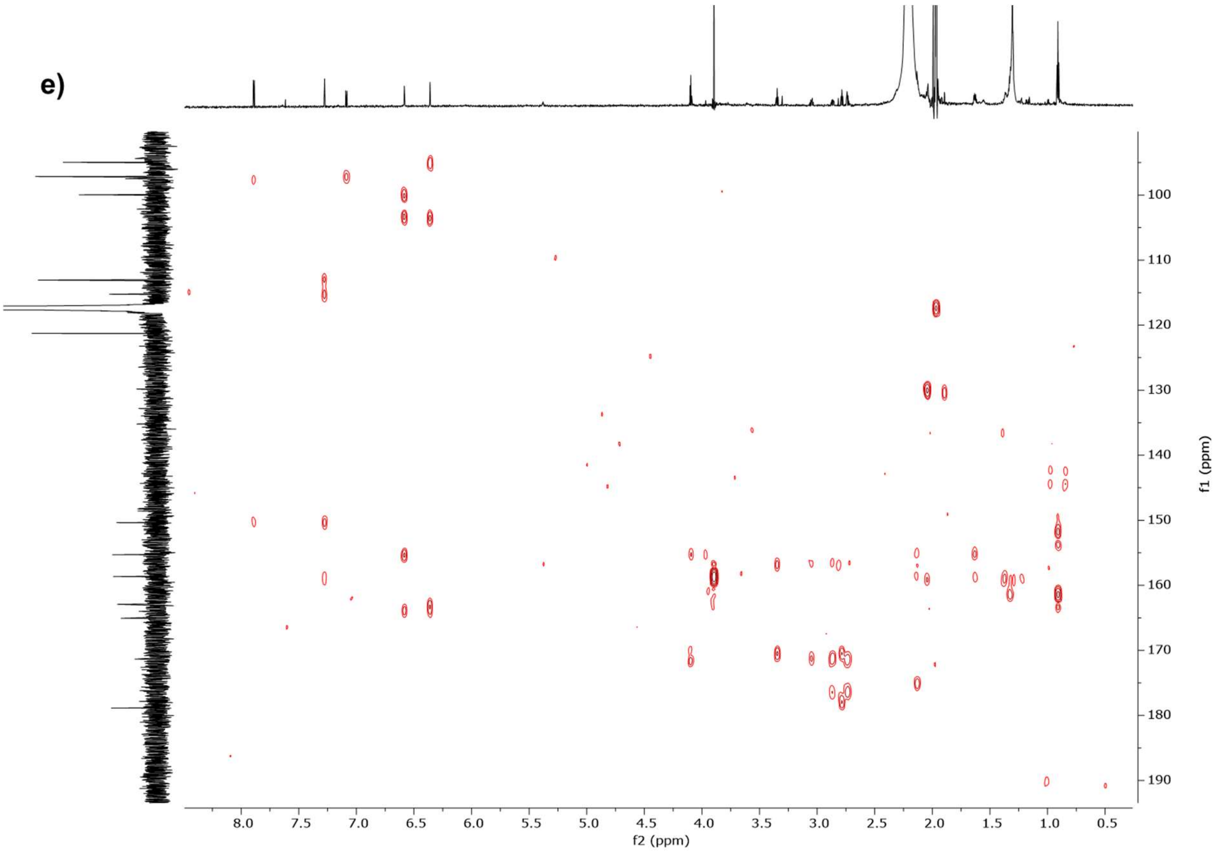

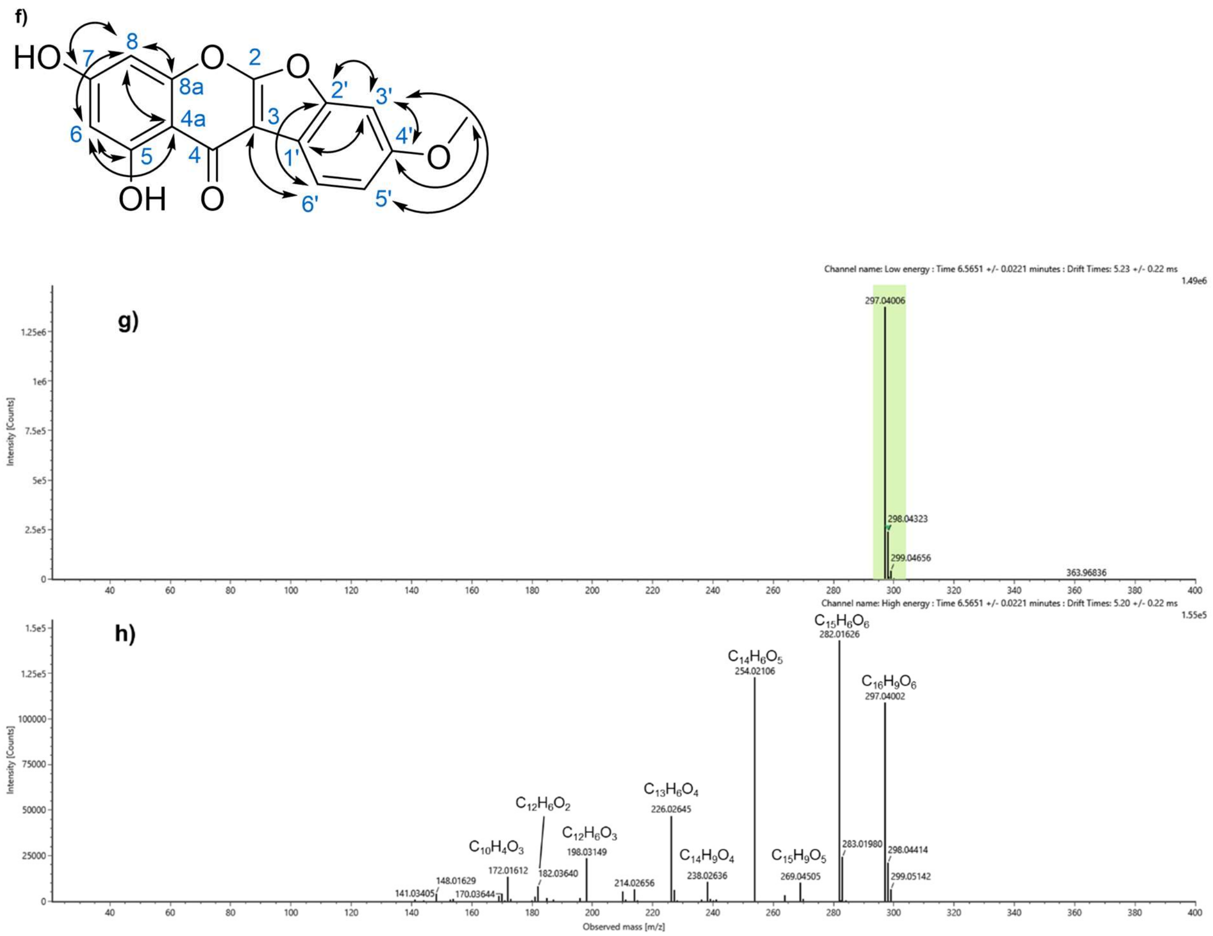
f)
HO
OH
O
8
2
8a
2'
3'
7
4a
3
4'
6
5
4
1'
6'
5'
g)
Channel name: Low energy : Time 6.5651 +/- 0.0221 minutes : Drift Times: 5.23 +/- 0.22 ms
1.49e6
297.04006
298.04323
299.04656
363.96836
Intensity [Counts]
h)
Channel name: High energy : Time 6.5651 +/- 0.0221 minutes : Drift Times: 5.20 +/- 0.22 ms
1.55e5
C15H6O6
282.01626
C14H6O5
254.02106
C16H9O6
297.04002
C13H6O4
226.02645
C12H6O2
182.03640
C12H6O3
198.03149
C10H4O3
172.01612
C14H9O4
238.02636
C15H9O5
269.04505
283.01980
298.04414
299.05142
214.02656
148.01629
141.03405
170.03644
Intensity [Counts]
Observed mass [m/z]

Fig. S9: ^{1}H (**a**), ^{13}C (**b**), COSY (**c**), HSQC (**d**), and HMBC (**e**) NMR spectra (CD_3OD, 600/151 MHz, 300 K) with key HMBC correlations (**f** and **g**) and low- (**h**) and high-energy (**i**) ToF-MS^{e} spectra of 4-hydroxy-2-(2′-hydroxy-4′-methoxyphenyl)-6-methoxybenzofuran-3-carbaldehyde (Lotusaldehyde, **15**). COSY: Correlation Spectroscopy; HSQC: Heteronuclear Single Quantum Coherence Spectroscopy; HMBC: Heteronuclear Multiple Bond Correlation Spectroscopy; NMR: Nuclear Magnetic Resonance Spectroscopy; CD_3OD: methanol-d_4; ToF-MS^{e}: Time-of-flight-Mass Spectrometry with elevated energy.

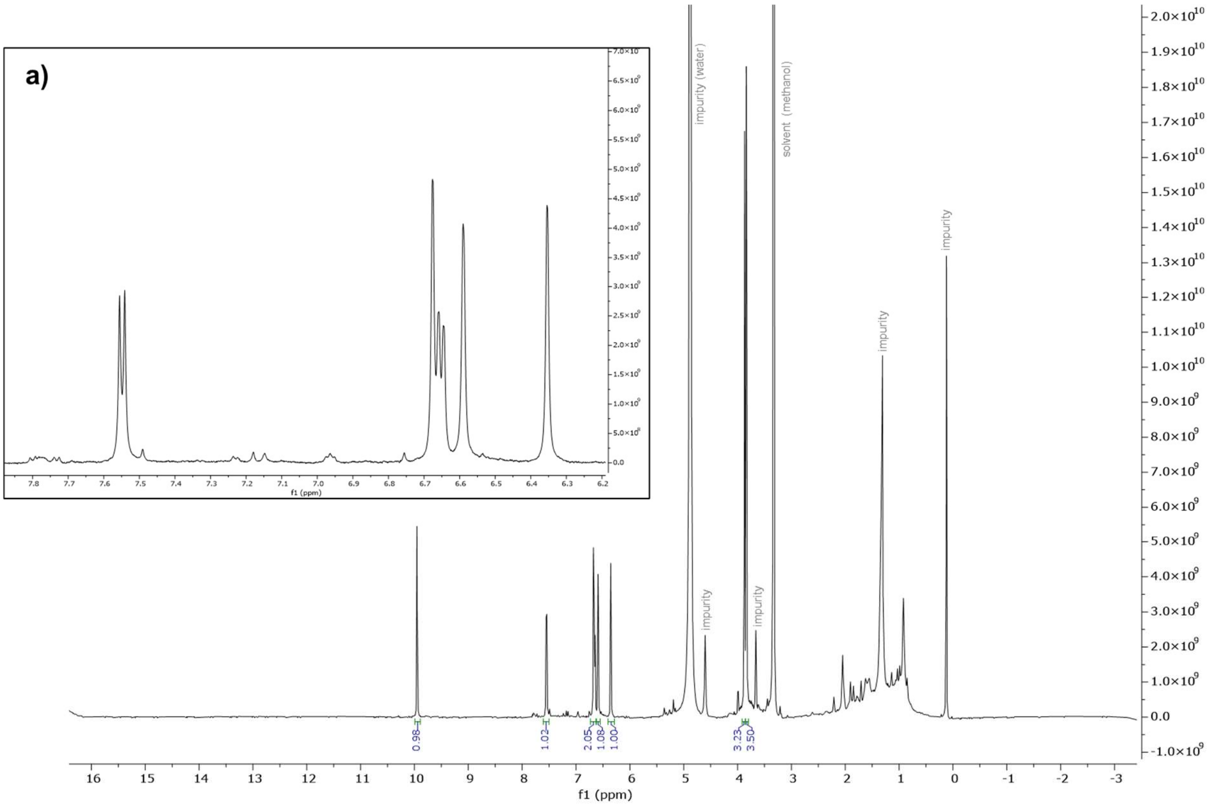

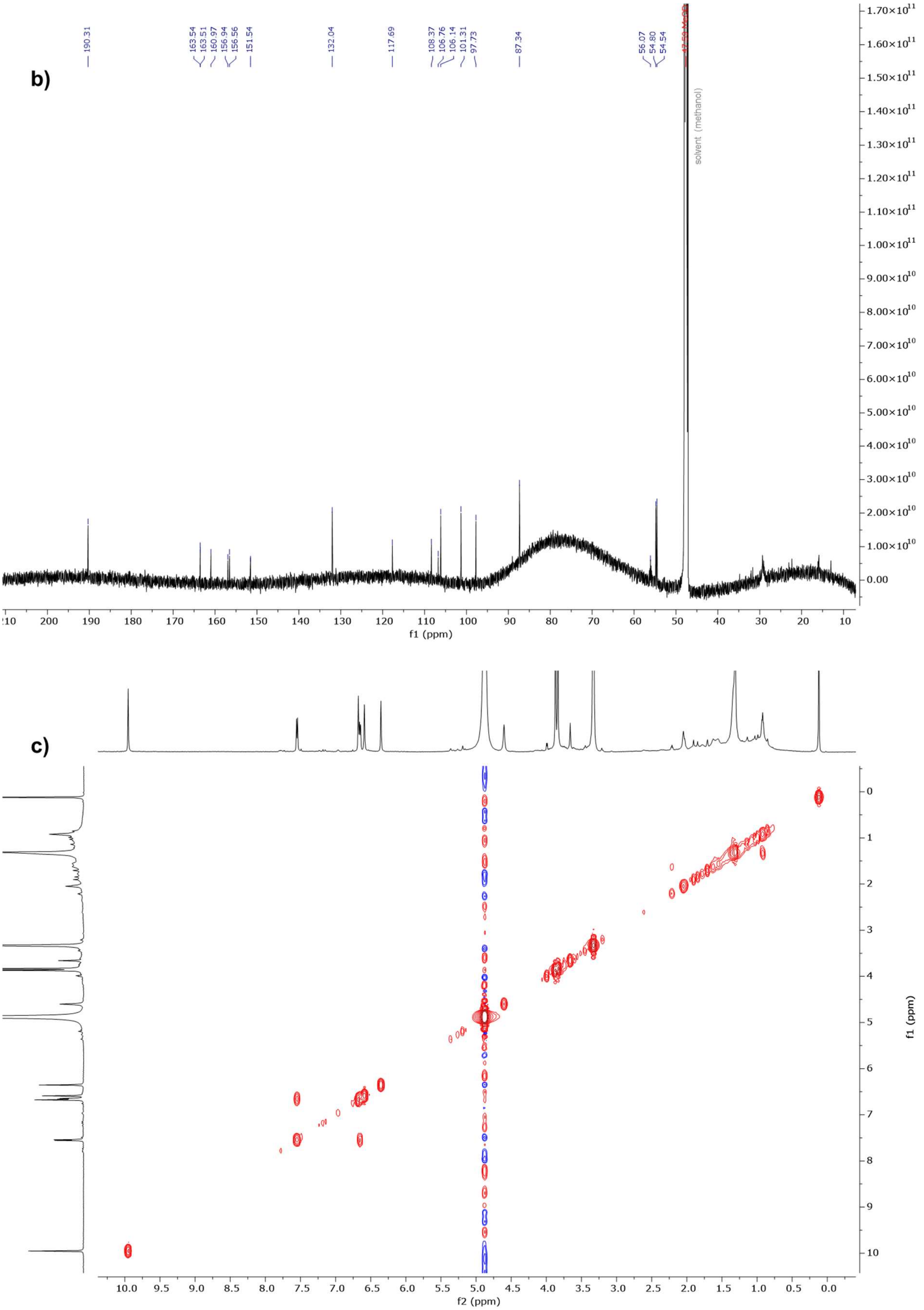
b)
190.31
163.54
163.51
160.97
156.94
156.56
151.54
132.04
117.69
108.37
106.76
106.14
101.31
97.73
87.34
56.07
54.80
54.54
solvent (methanol)
f1 (ppm)
c)
f2 (ppm)
f1 (ppm)

d)

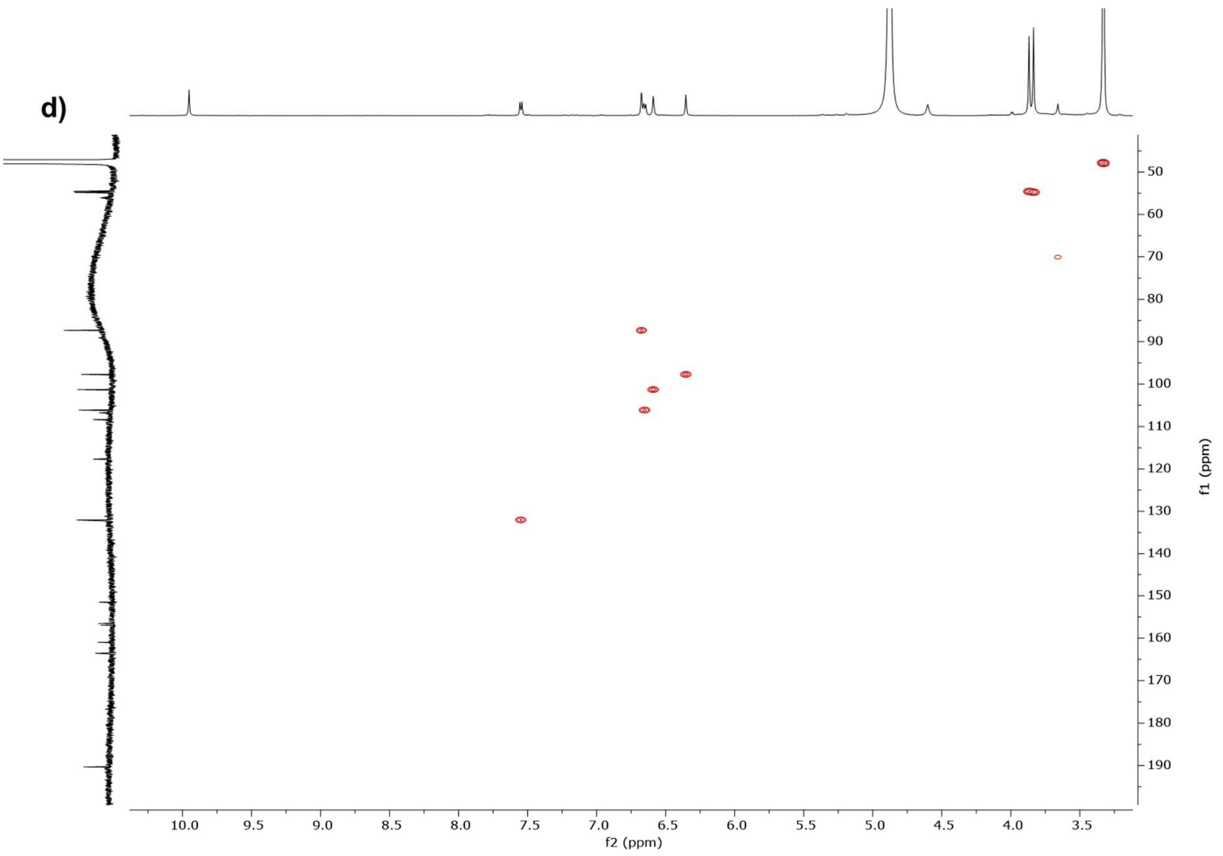

10.0 9.5 9.0 8.5 8.0 7.5 7.0 6.5 6.0 5.5 5.0 4.5 4.0 3.5
f2 (ppm)
50 60 70 80 90 100 110 120 130 140 150 160 170 180 190
f1 (ppm)

e)

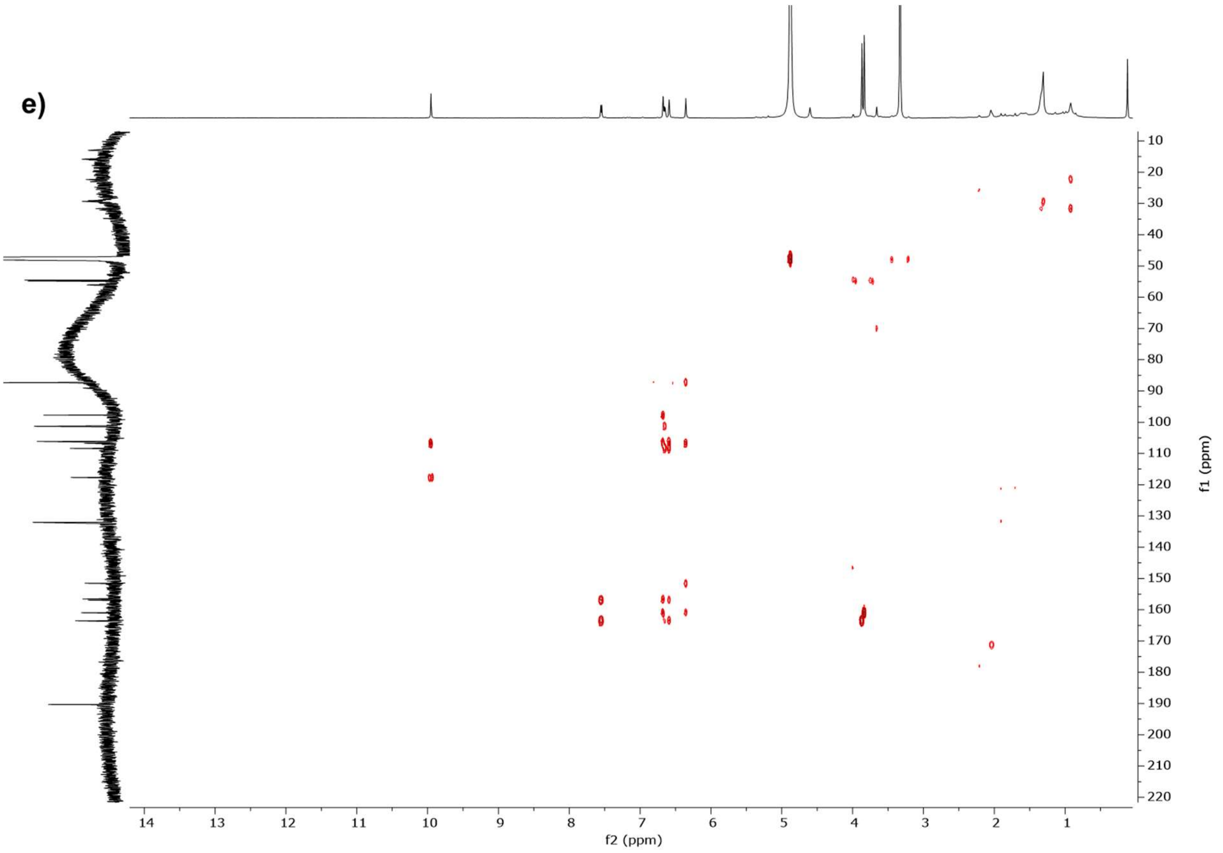

14 13 12 11 10 9 8 7 6 5 4 3 2 1
f2 (ppm)
10 20 30 40 50 60 70 80 90 100 110 120 130 140 150 160 170 180 190 200 210 220
f1 (ppm)

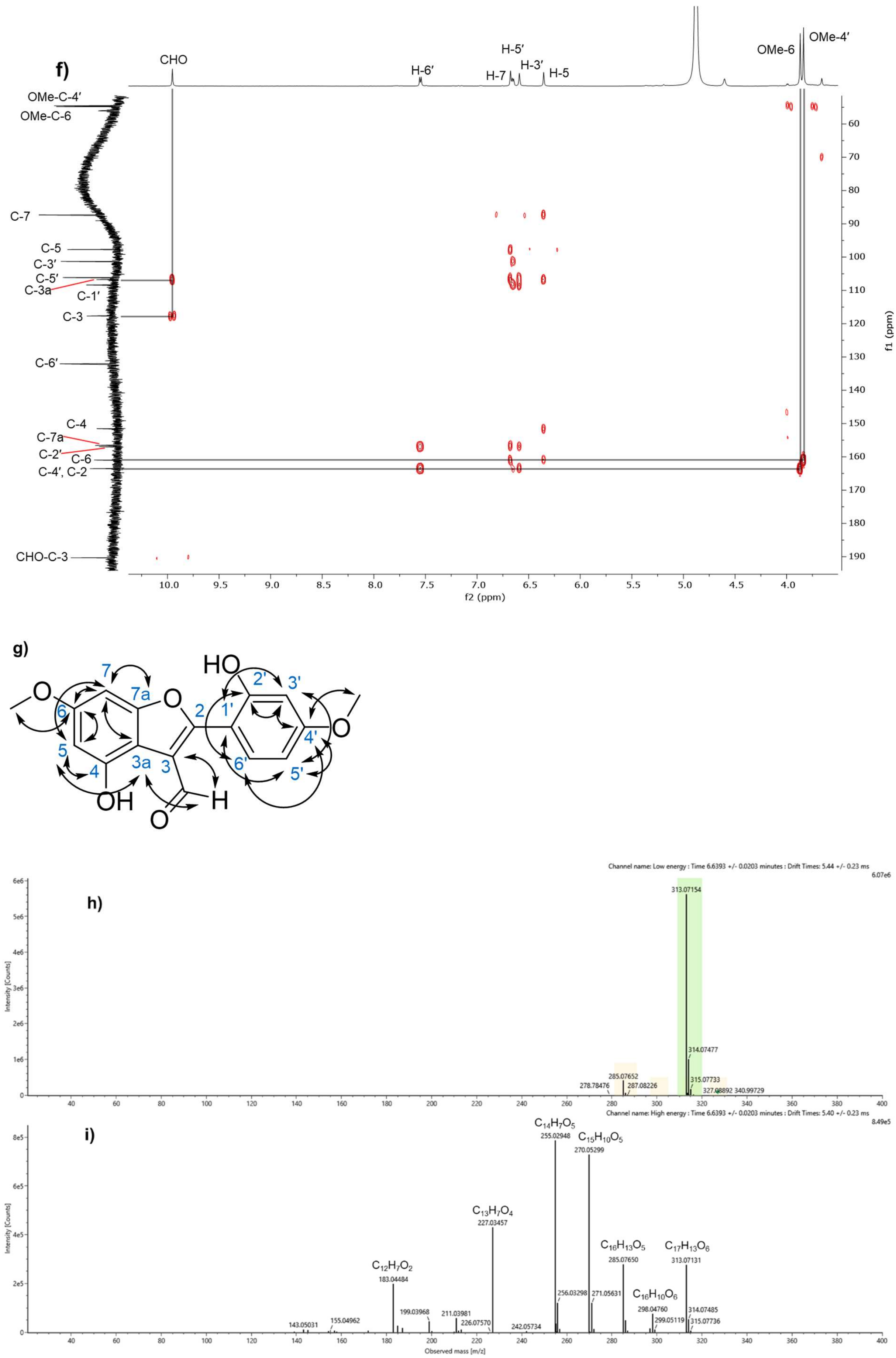
f)
CHO
H-6′
H-5′
H-7
H-3′
H-5
OMe-6
OMe-4′
OMe-C-4′
OMe-C-6
C-7
C-5
C-3′
C-5′
C-3a
C-1′
C-3
C-6′
C-4
C-7a
C-2′
C-6
C-4′, C-2
CHO-C-3
f2 (ppm)
f1 (ppm)
g)
h)
313.07154
314.07477
285.07652
315.07733
i)
$C_{14}H_7O_5$ 255.02948
$C_{15}H_{10}O_5$ 270.05299
$C_{13}H_7O_4$ 227.03457
$C_{12}H_7O_2$ 183.04484
$C_{16}H_{13}O_5$ 285.07650
$C_{17}H_{13}O_6$ 313.07131
$C_{16}H_{10}O_6$ 298.04760
Observed mass [m/z]
Intensity [Counts]

Fig. S10: ^{1}H (**a**), ^{13}C (**b**), COSY (**c**), HSQC (**d**), and HMBC (**e**) NMR spectra (CD_3OD, 600/151 MHz, 300 K) with key HMBC correlations (**f** and **g**) and low- (**h**) and high-energy (**i**) ToF-MS^{e} spectra of 7-hydroxy-3,9-dimethoxypterocarp-6a-ene (Lotuscarpene, **16**). COSY: Correlation Spectroscopy; HSQC: Heteronuclear Single Quantum Coherence Spectroscopy; HMBC: Heteronuclear Multiple Bond Correlation Spectroscopy; NMR: Nuclear Magnetic Resonance Spectroscopy; CD_3OD: methanol-d$_4$; ToF-MS^{e}: Time-of-flight-Mass Spectrometry with elevated energy.

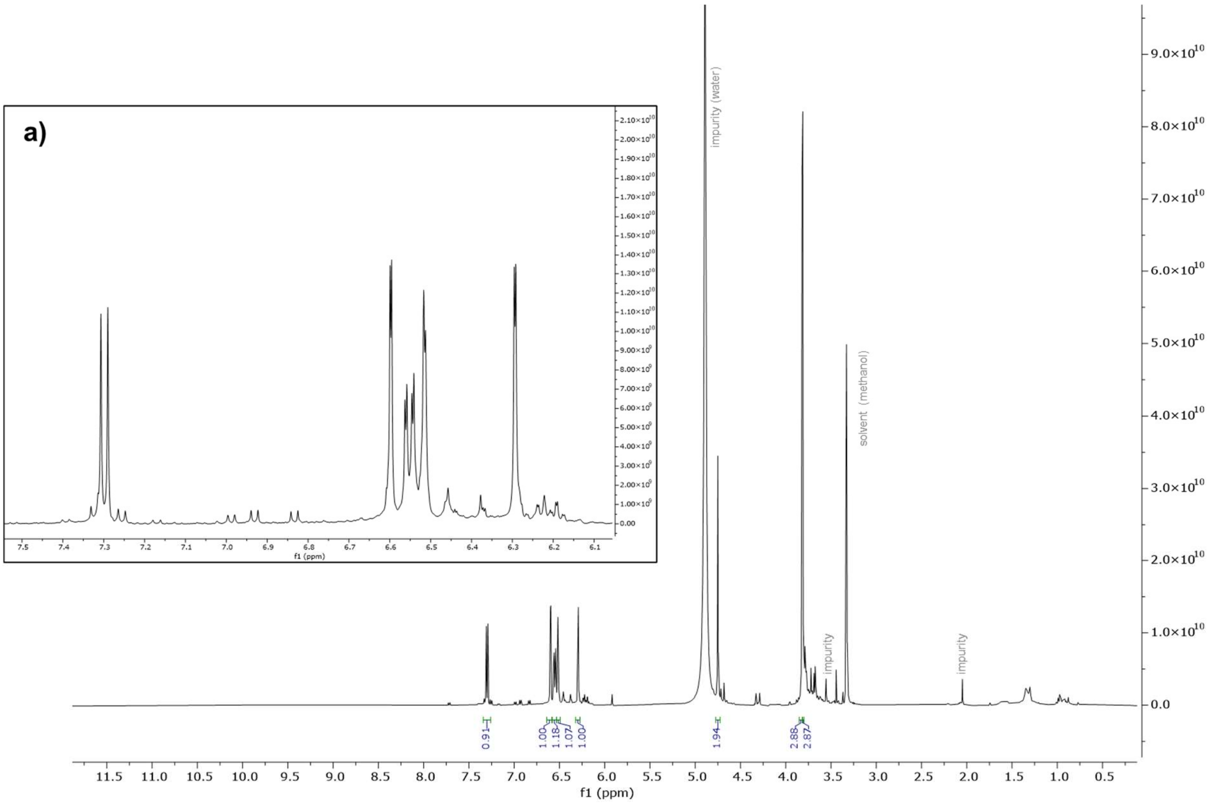

b)

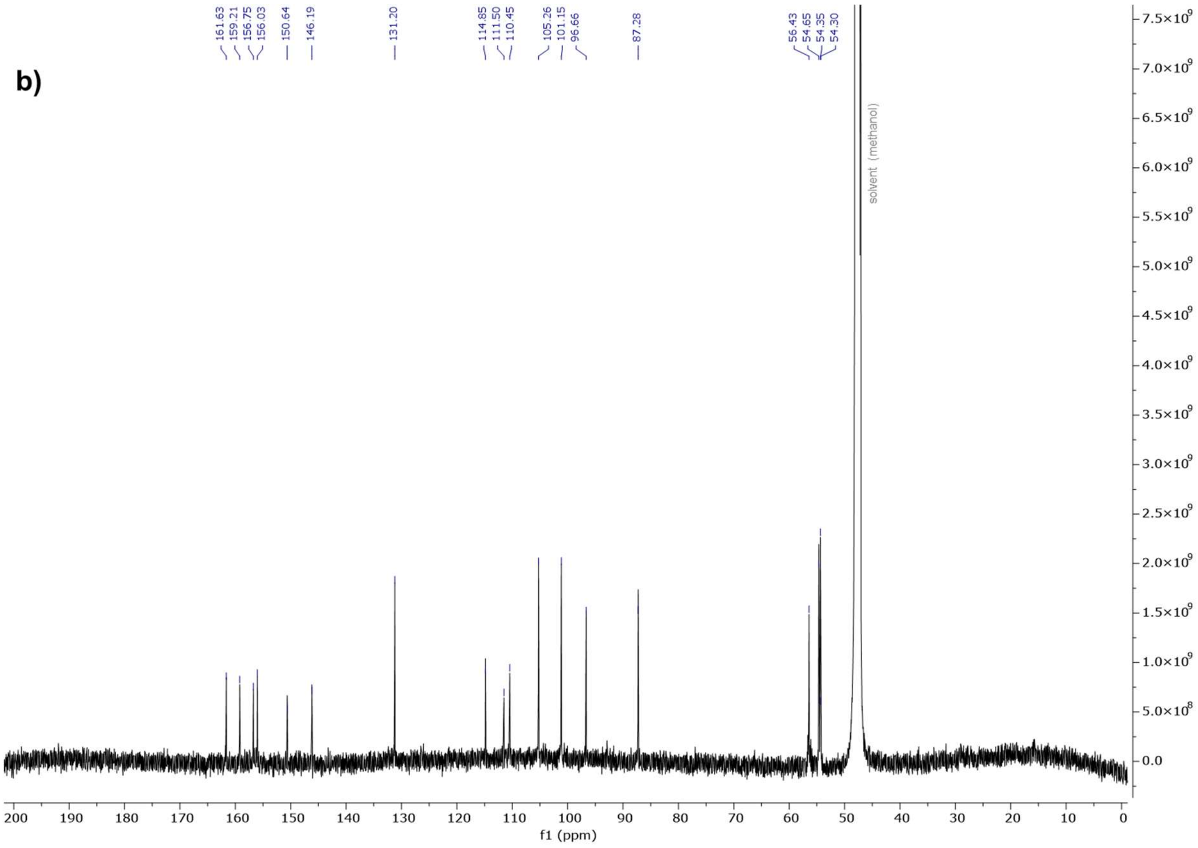
161.63
159.21
156.75
156.03
150.64
146.19
131.20
114.85
111.50
110.45
105.26
101.15
96.66
87.28
56.43
54.65
54.35
54.30
solvent (methanol)
f1 (ppm)

c)

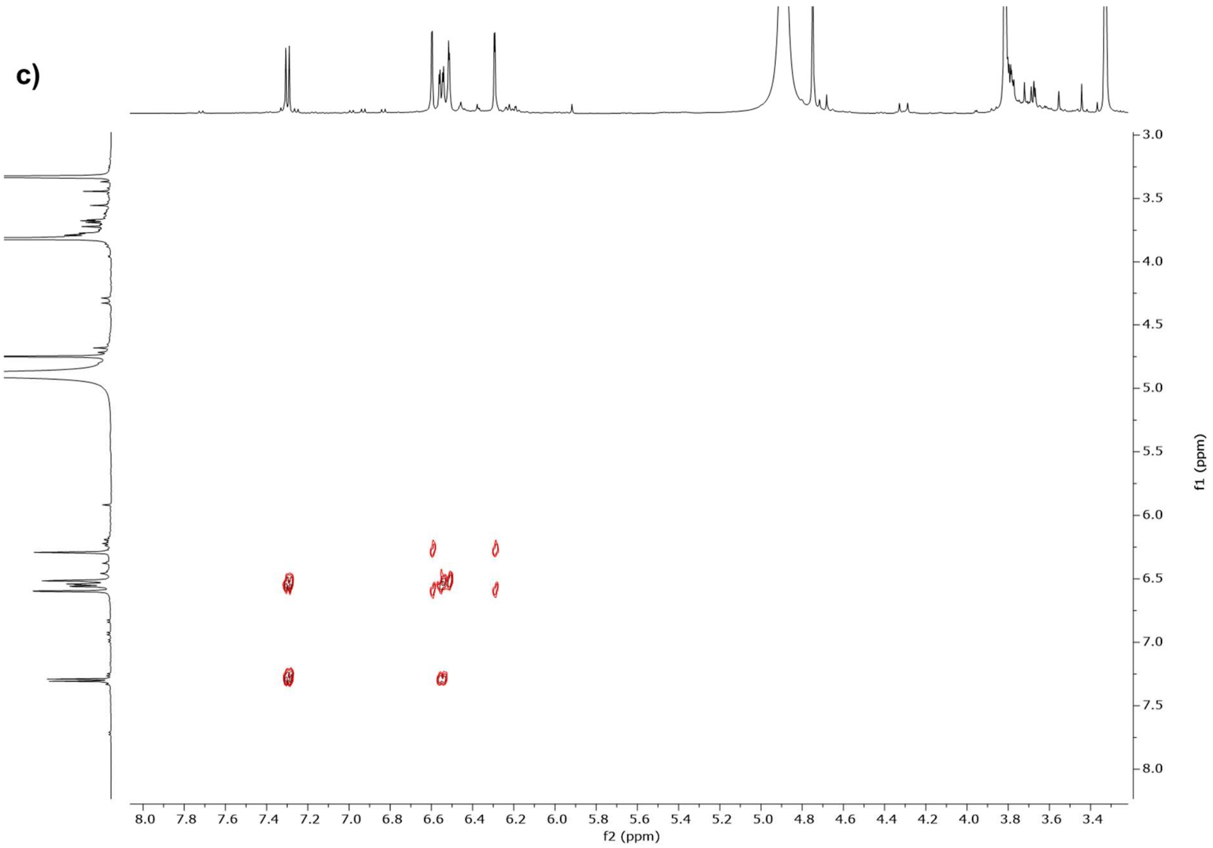
f2 (ppm)
f1 (ppm)

d)

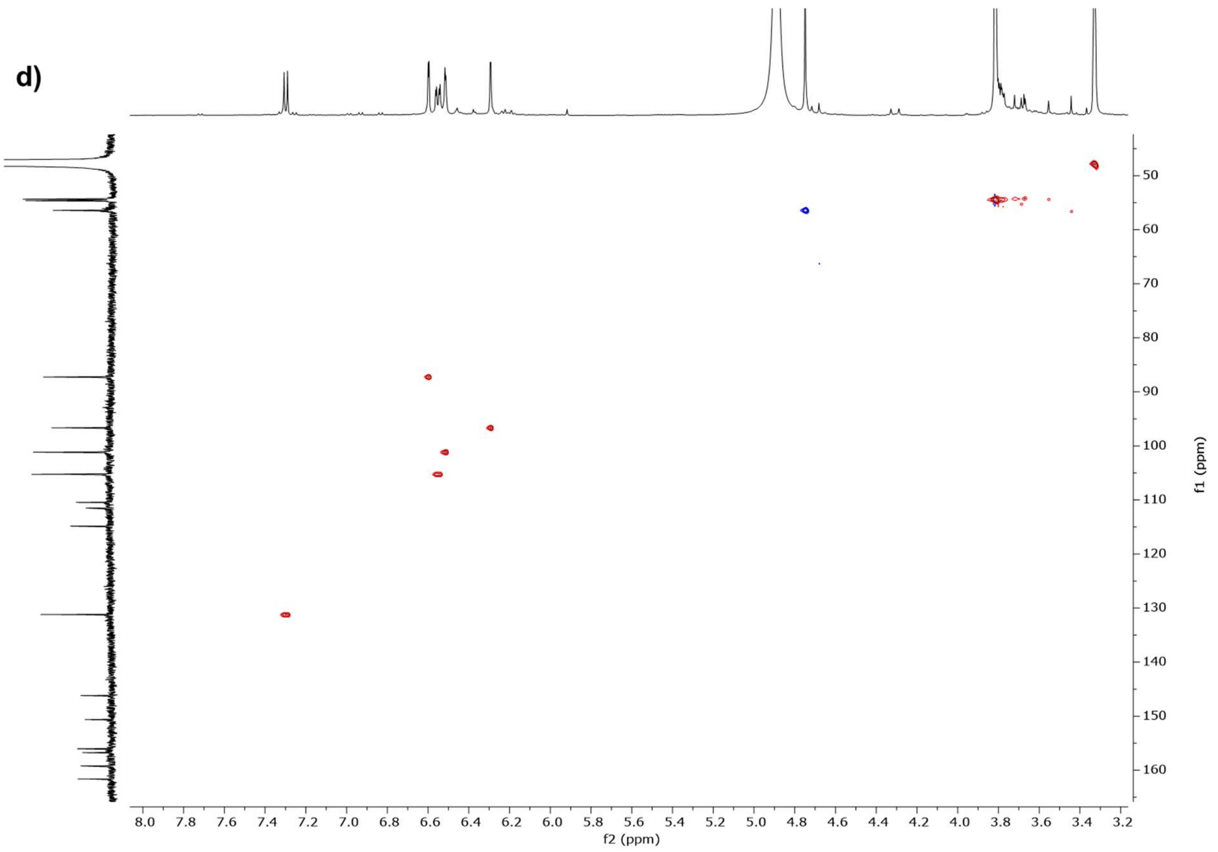

8.0 7.8 7.6 7.4 7.2 7.0 6.8 6.6 6.4 6.2 6.0 5.8 5.6 5.4 5.2 5.0 4.8 4.6 4.4 4.2 4.0 3.8 3.6 3.4 3.2
f2 (ppm)
50 60 70 80 90 100 110 120 130 140 150 160
f1 (ppm)

e)

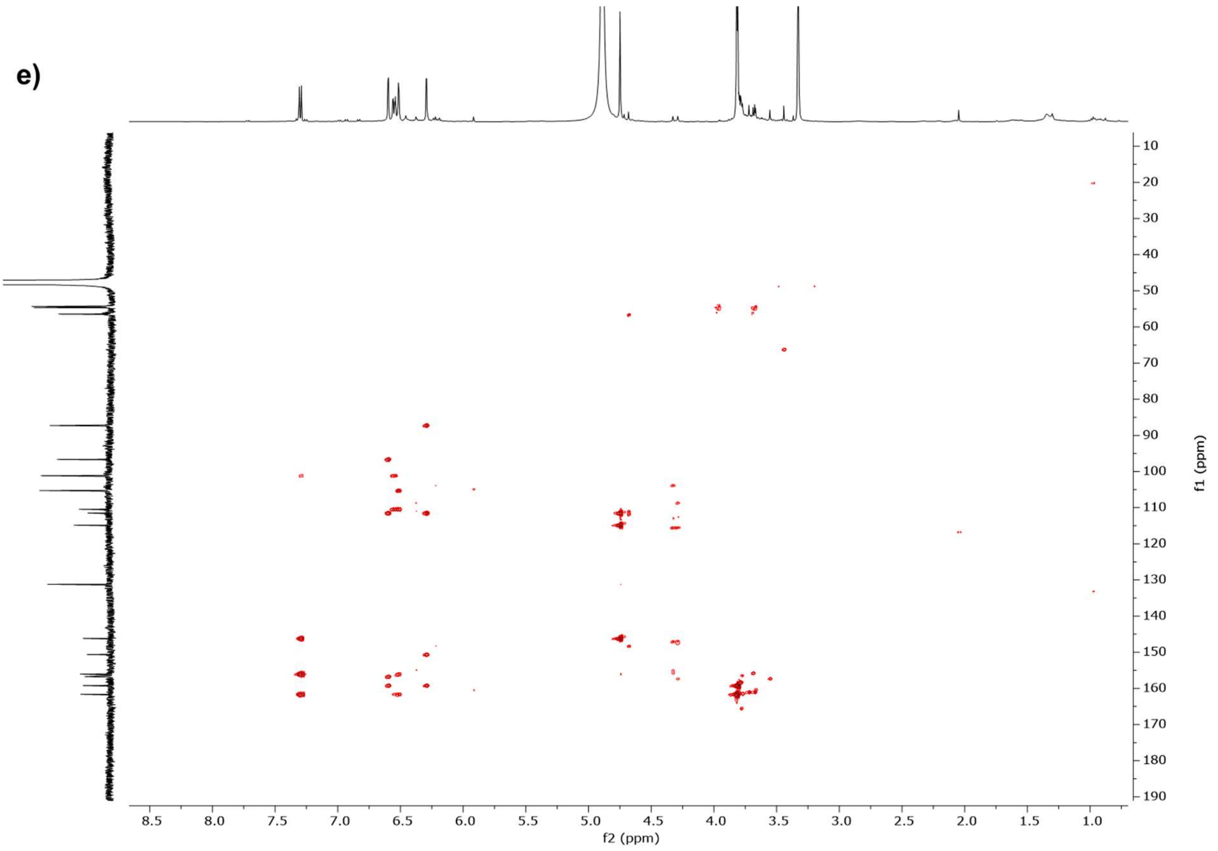

8.5 8.0 7.5 7.0 6.5 6.0 5.5 5.0 4.5 4.0 3.5 3.0 2.5 2.0 1.5 1.0
f2 (ppm)
10 20 30 40 50 60 70 80 90 100 110 120 130 140 150 160 170 180 190
f1 (ppm)

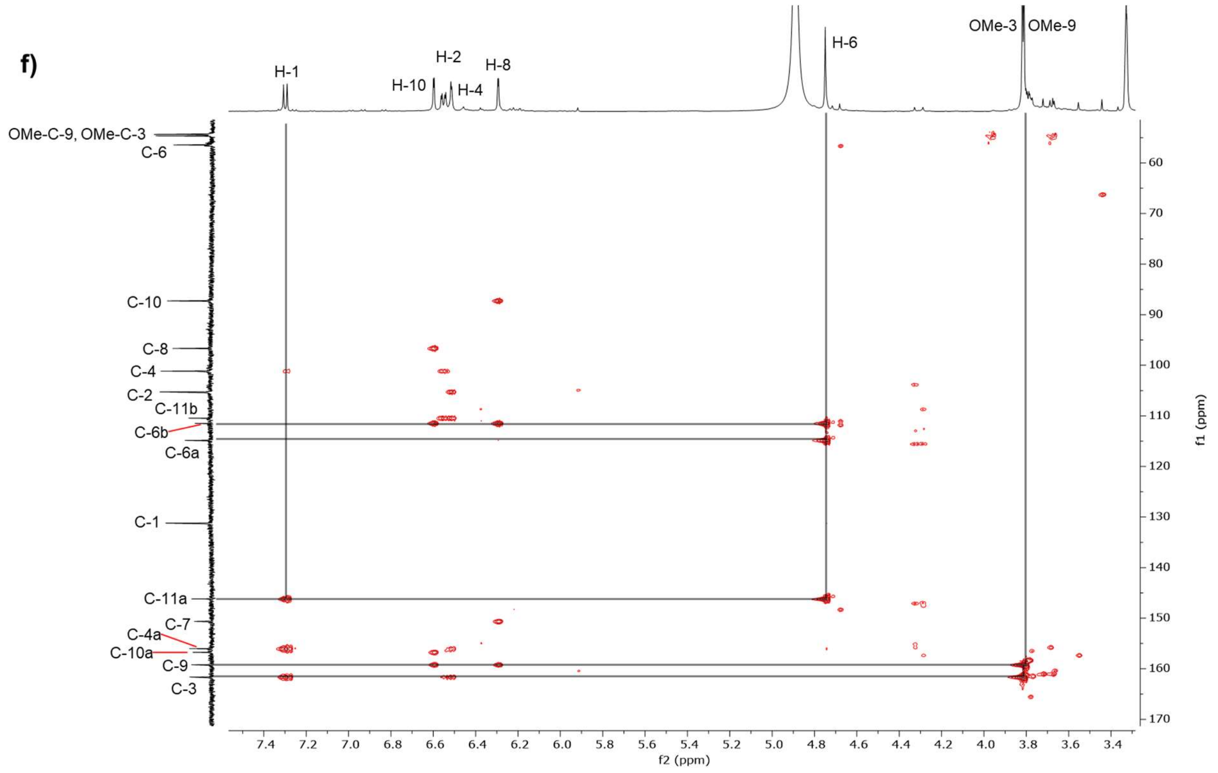
f)
H-1
H-10
H-2
H-4
H-8
H-6
OMe-3
OMe-9
OMe-C-9, OMe-C-3
C-6
C-10
C-8
C-4
C-2
C-11b
C-6b
C-6a
C-1
C-11a
C-7
C-4a
C-10a
C-9
C-3
f2 (ppm)
f1 (ppm)

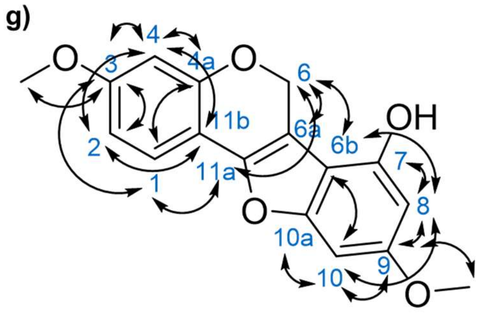
g)
OH

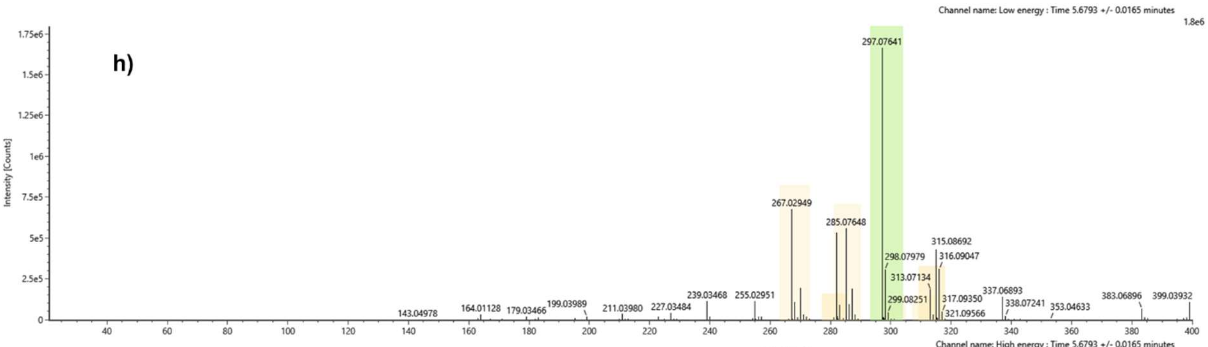
h)
Channel name: Low energy : Time 5.6793 +/- 0.0165 minutes
1.8e6
Intensity [Counts]
297.07641
267.02949
285.07648
315.08692
316.09047
298.07979
313.07134
299.08251
317.09350
321.09566
337.06893
338.07241
353.04633
383.06896
399.03932
143.04978
164.01128
179.03466
199.03989
211.03980
227.03484
239.03468
255.02951

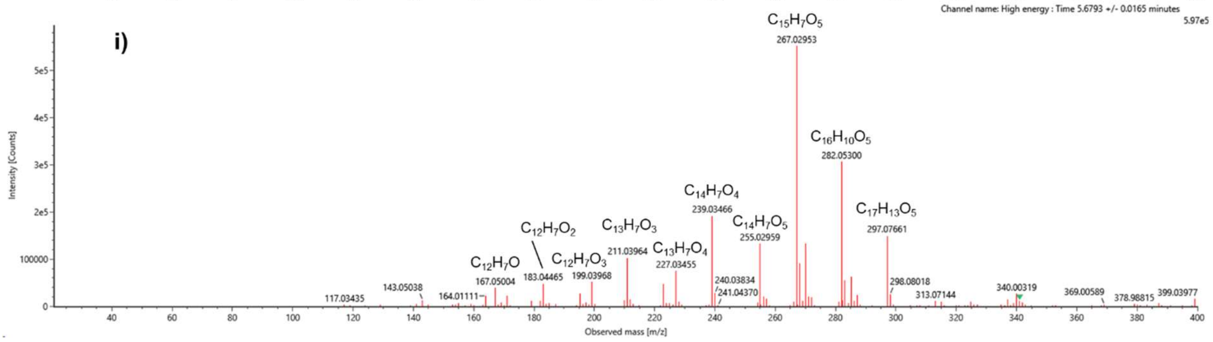
i)
Channel name: High energy : Time 5.6793 +/- 0.0165 minutes
5.97e5
Intensity [Counts]
$C_{15}H_7O_5$
267.02953
$C_{16}H_{10}O_5$
282.05300
$C_{14}H_7O_4$
239.03466
$C_{17}H_{13}O_5$
297.07661
$C_{14}H_7O_5$
255.02959
$C_{12}H_7O_2$
$C_{13}H_7O_3$
211.03964
$C_{13}H_7O_4$
227.03455
$C_{12}H_7O_3$
199.03968
$C_{12}H_7O$
167.05004
183.04465
240.03834
241.04370
298.08018
313.07144
340.00319
369.00589
378.98815
399.03977
117.03435
143.05038
164.01111
Observed mass [m/z]

Fig. S11: ^{1}H (**a**), ^{13}C (**b**), COSY (**c**), HSQC (**d**), and HMBC (**e**) NMR spectra (CD_3CN, 600/151 MHz, 300 K) with key HMBC correlations (**f** and **g**) and low- (**h**) and high-energy (**i**) ToF-MS^{e} spectra of 4,6-dihydroxy-2-(2′-hydroxy-4′-methoxyphenyl)-3-(methoxymethyl)-benzofuran (**25**). COSY: Correlation Spectroscopy; HSQC: Heteronuclear Single Quantum Coherence Spectroscopy; HMBC: Heteronuclear Multiple Bond Correlation Spectroscopy; NMR: Nuclear Magnetic Resonance Spectroscopy; CD_3CN: acetonitrile-d$_3$; ToF-MS^{e}: Time-of-flight-Mass Spectrometry with elevated energy.

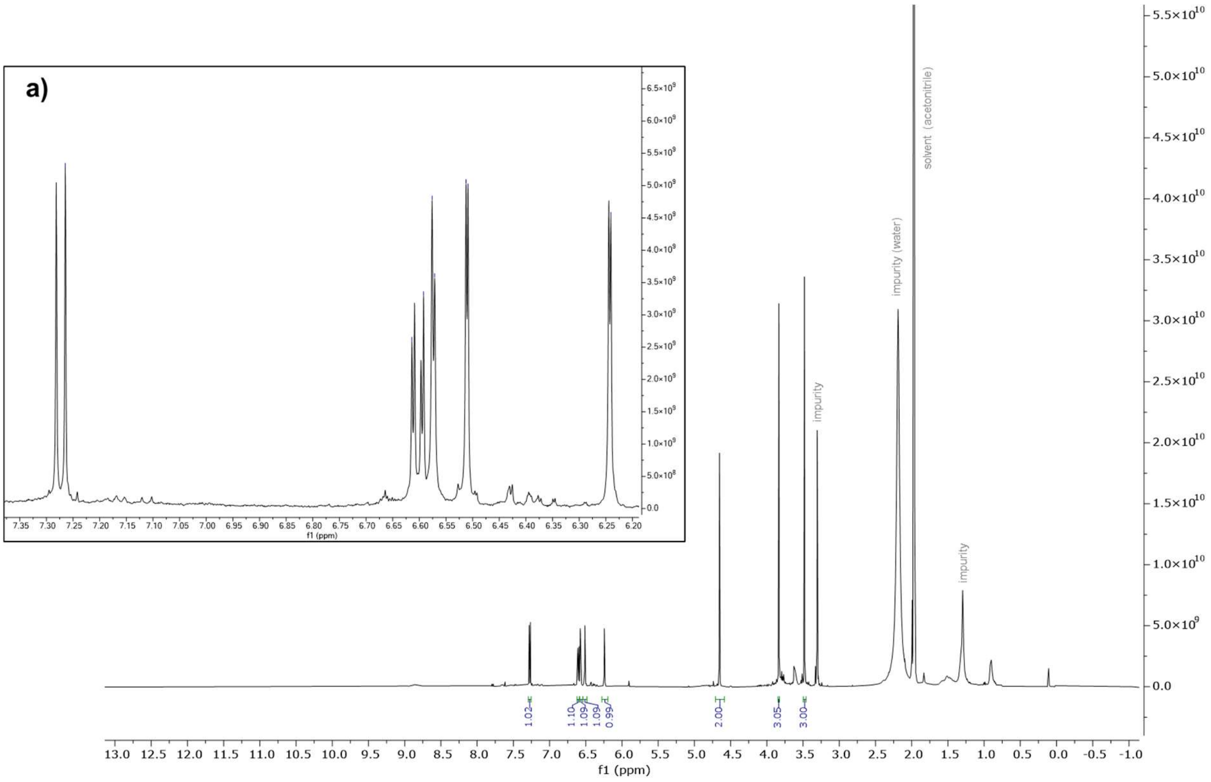

b)

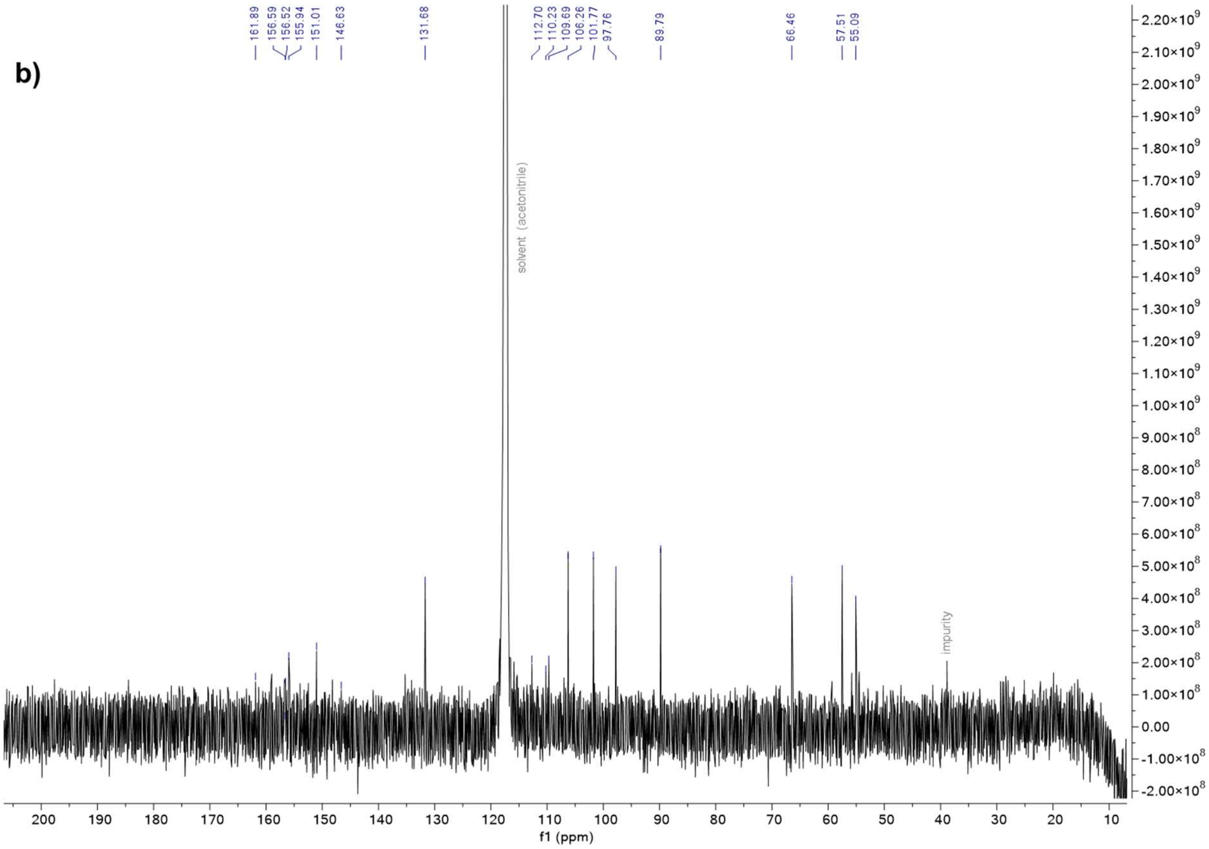
161.89
156.59
156.52
155.94
151.01
146.63
131.68
112.70
110.23
109.69
106.26
101.77
97.76
89.79
66.46
57.51
55.09
solvent (acetonitrile)
impurity
f1 (ppm)

c)

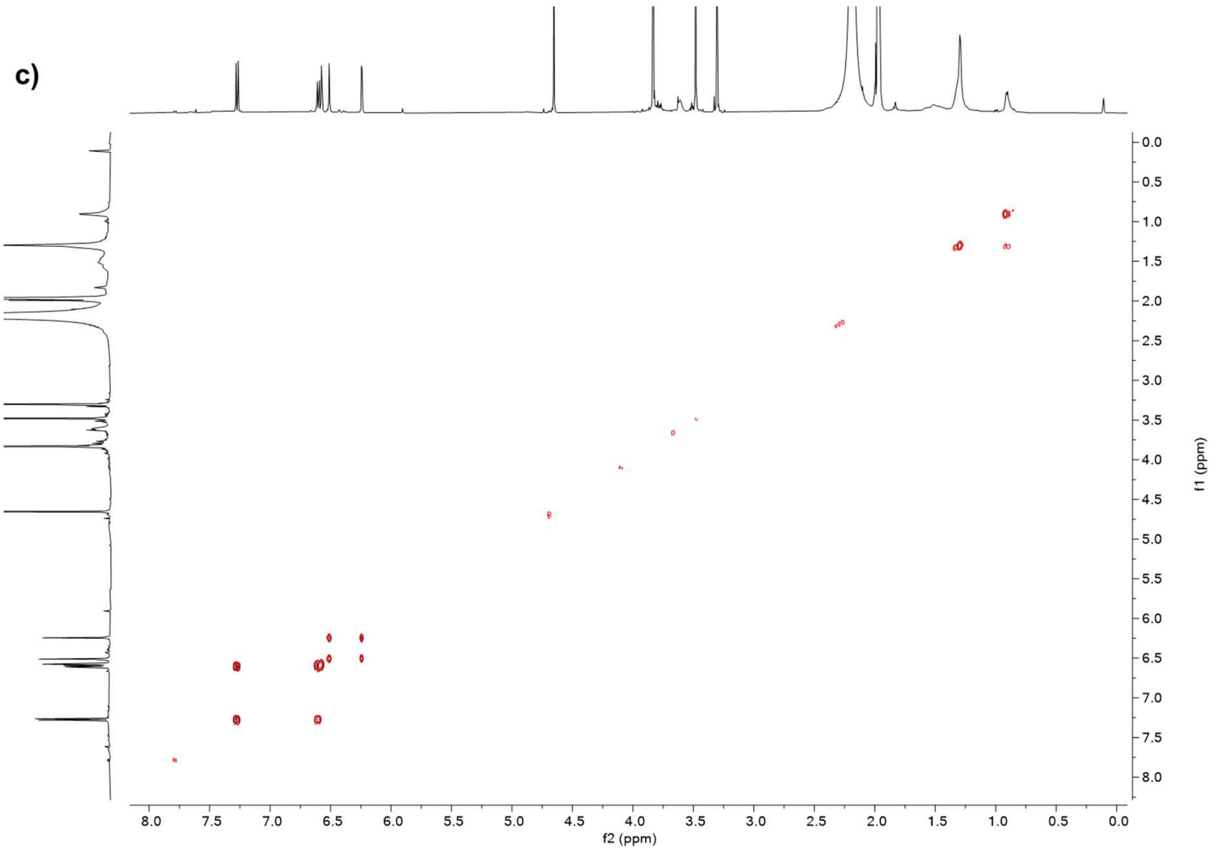
f2 (ppm)
f1 (ppm)

d)

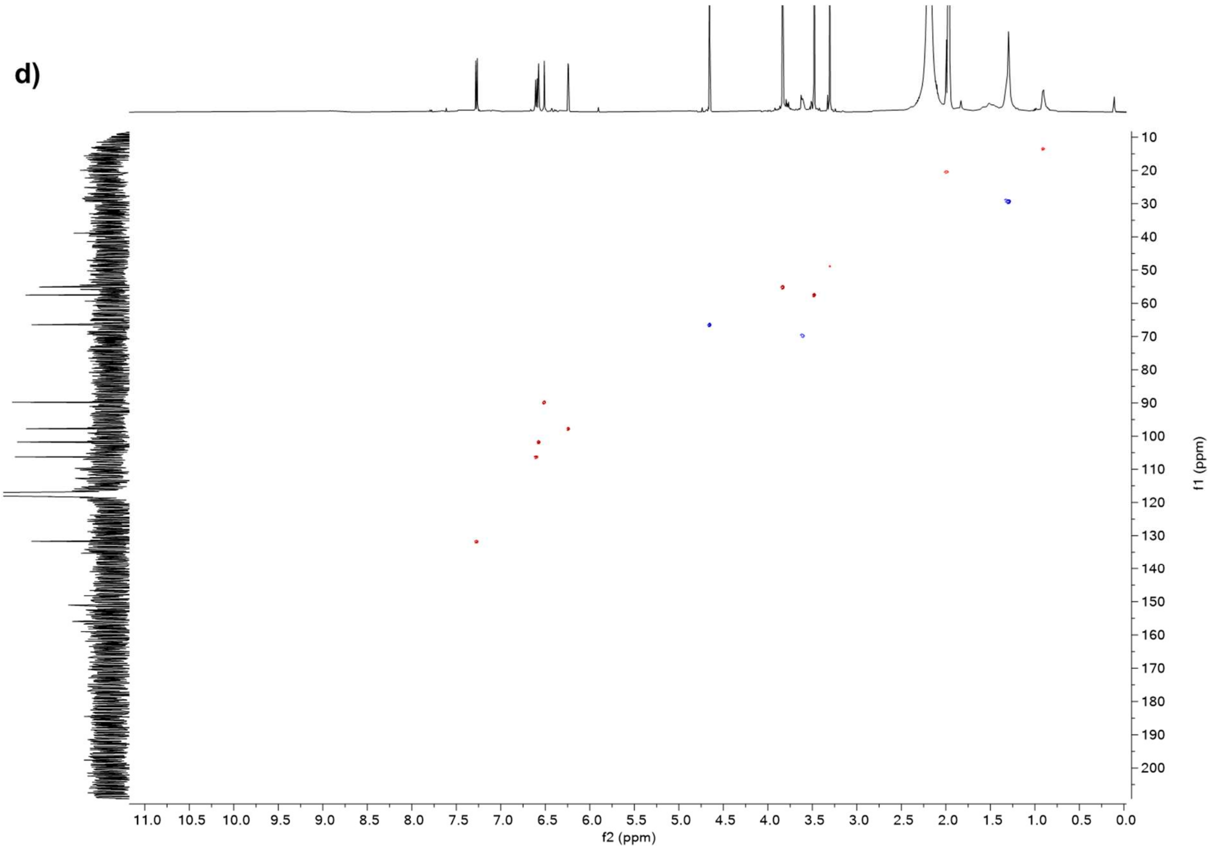

e)

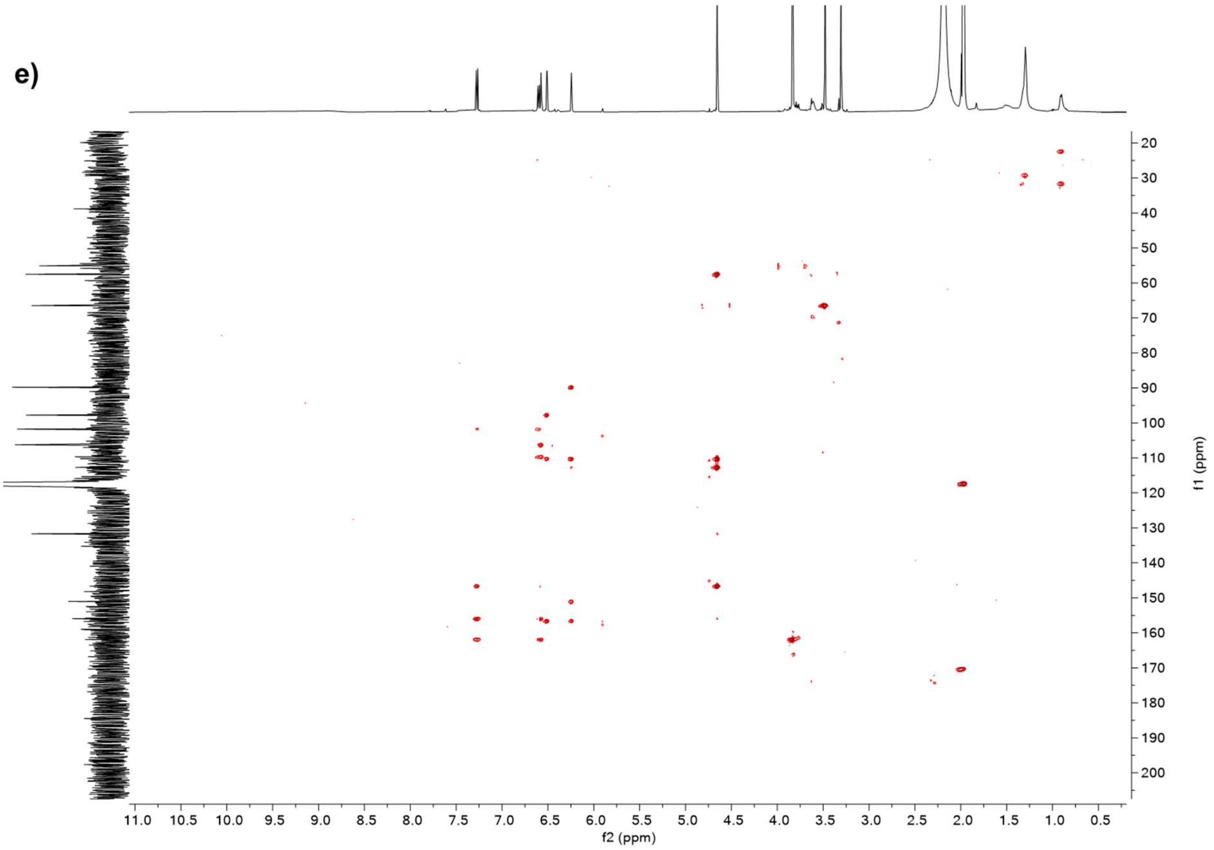

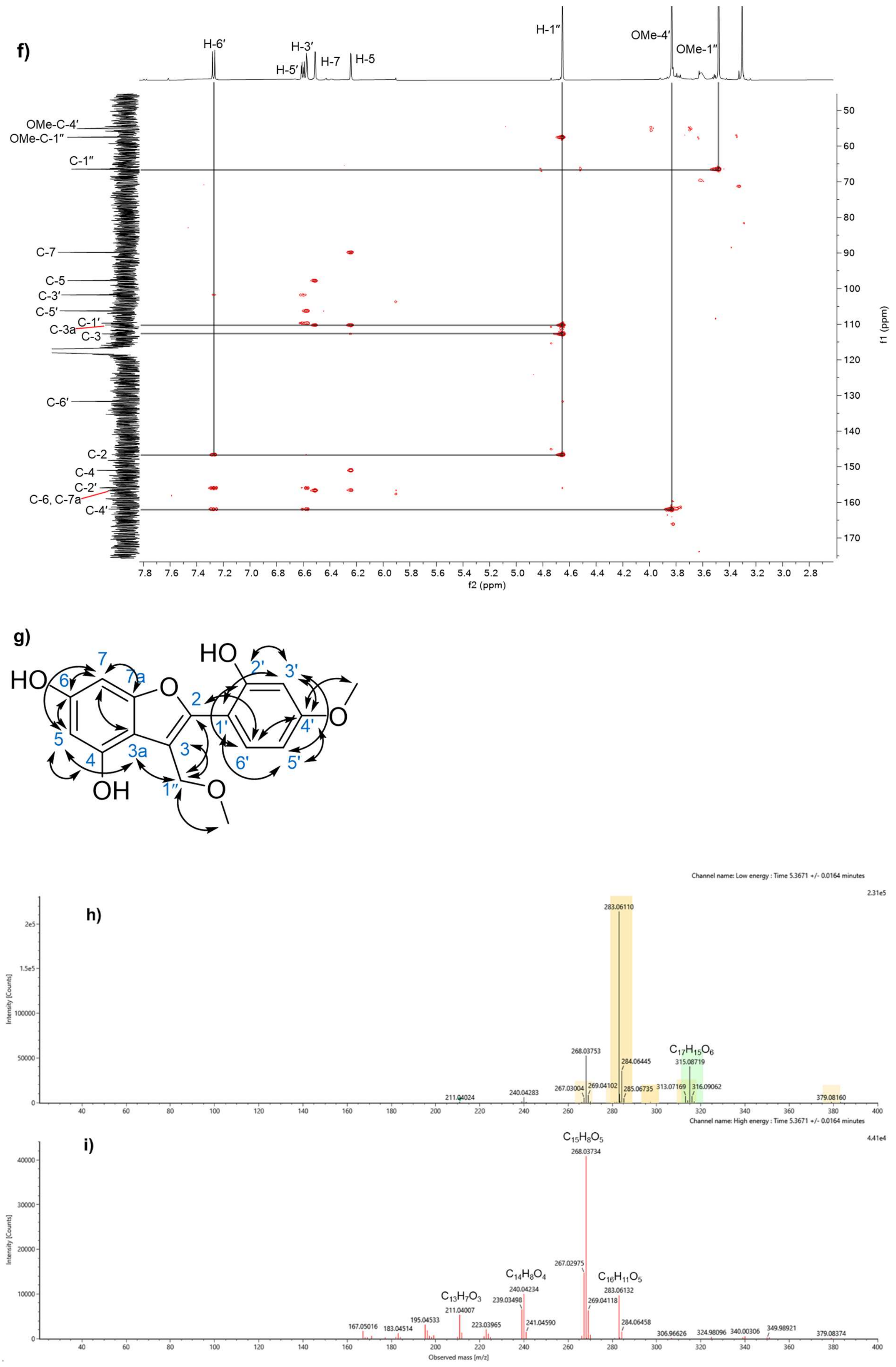
f)
H-6′
H-3′
H-5′
H-7
H-5
H-1″
OMe-4′
OMe-1″
OMe-C-4′
OMe-C-1″
C-1″
C-7
C-5
C-3′
C-5′
C-1′
C-3a
C-3
C-6′
C-2
C-4
C-2′
C-6, C-7a
C-4′
f2 (ppm)
f1 (ppm)
g)
HO
OH
O
h)
Channel name: Low energy : Time 5.3671 +/- 0.0164 minutes
2.31e5
Intensity [Counts]
283.06110
268.03753
284.06445
$C_{17}H_{15}O_6$
315.08719
267.03004
269.04102
285.06735
313.07169
316.09062
240.04283
211.04024
379.08160
Channel name: High energy : Time 5.3671 +/- 0.0164 minutes
i)
4.41e4
$C_{15}H_8O_5$
268.03734
267.02975
$C_{14}H_8O_4$
240.04234
$C_{16}H_{11}O_5$
283.06132
239.03498
269.04118
$C_{13}H_7O_3$
211.04007
195.04533
223.03965
241.04590
284.06458
167.05016
183.04514
306.96626
324.98096
340.00306
349.98921
379.08374
Observed mass [m/z]

Fig. S12: ^{1}H (**a**), ^{13}C (**b**), COSY (**c**), HSQC (**d**), and HMBC (**e**) NMR spectra (CD_3CN, 600/151 MHz, 300 K) with key HMBC correlations (**f** and **g**) and low- (**h**) and high-energy (**i**) ToF-MS^{e} spectra of 4-Hydroxy-2-(2′-hydroxy-4′-methoxyphenyl)-6-methoxy-3-(methoxymethyl)benzofuran (**26**). COSY: Correlation Spectroscopy; HSQC: Heteronuclear Single Quantum Coherence Spectroscopy; HMBC: Heteronuclear Multiple Bond Correlation Spectroscopy; NMR: Nuclear Magnetic Resonance Spectroscopy; CD_3CN: acetonitrile-d_3; ToF-MS^{e}: Time-of-flight-Mass Spectrometry with elevated energy.

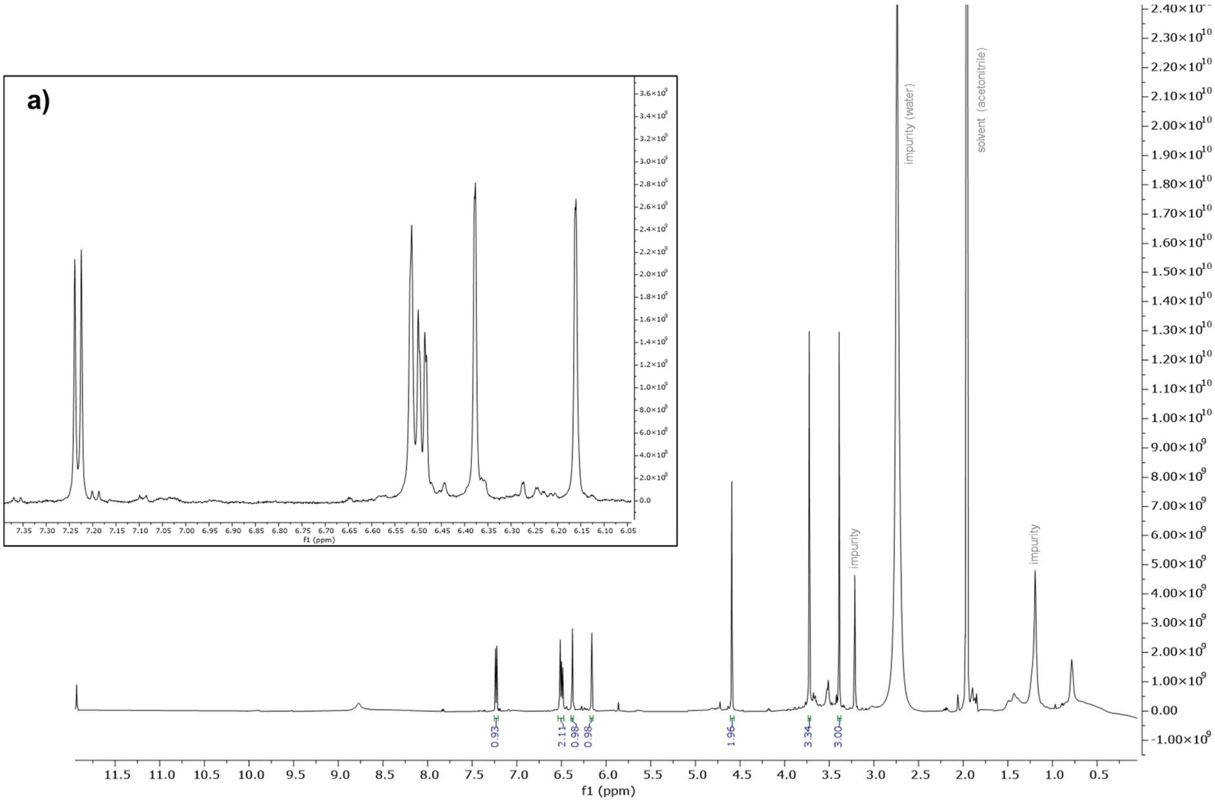

b)

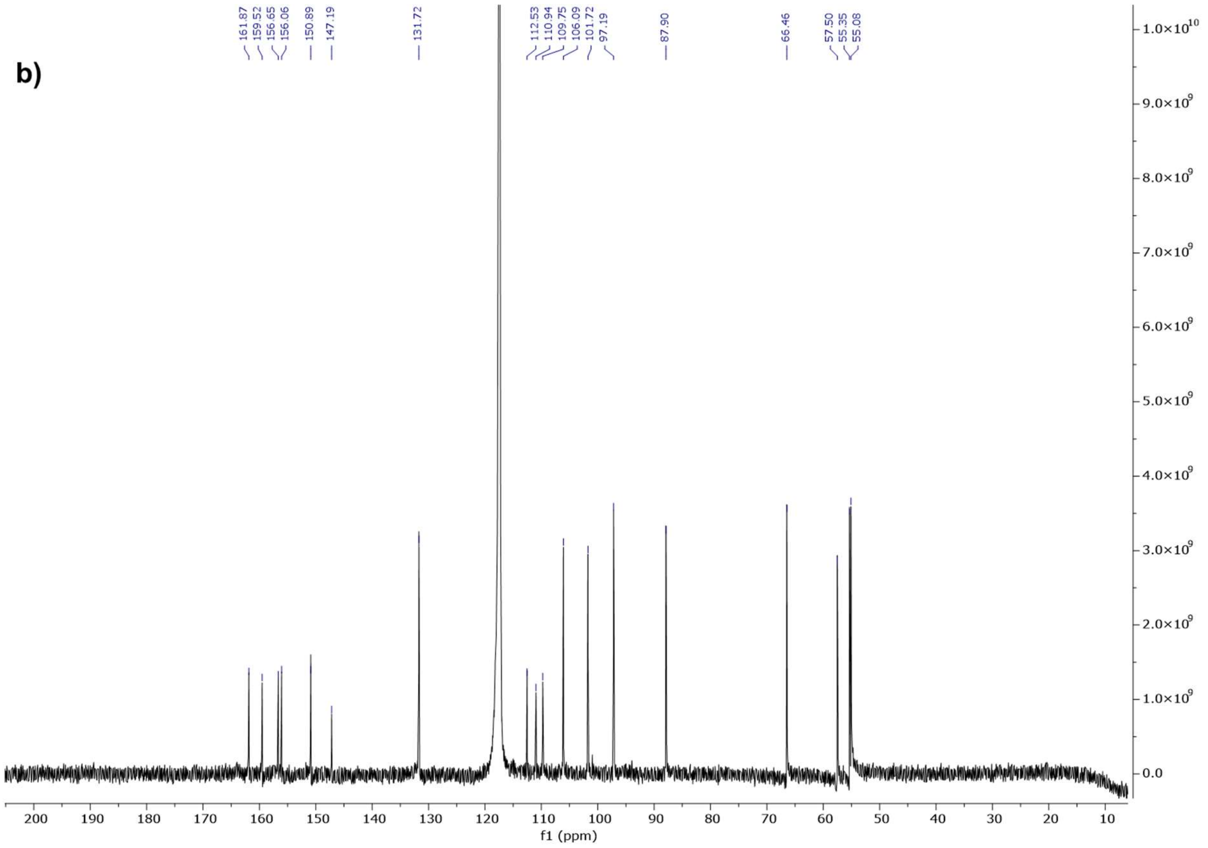
161.87
159.52
156.65
156.06
150.89
147.19
131.72
112.53
110.94
109.75
106.09
101.72
97.19
87.90
66.46
57.50
55.35
55.08
f1 (ppm)

c)

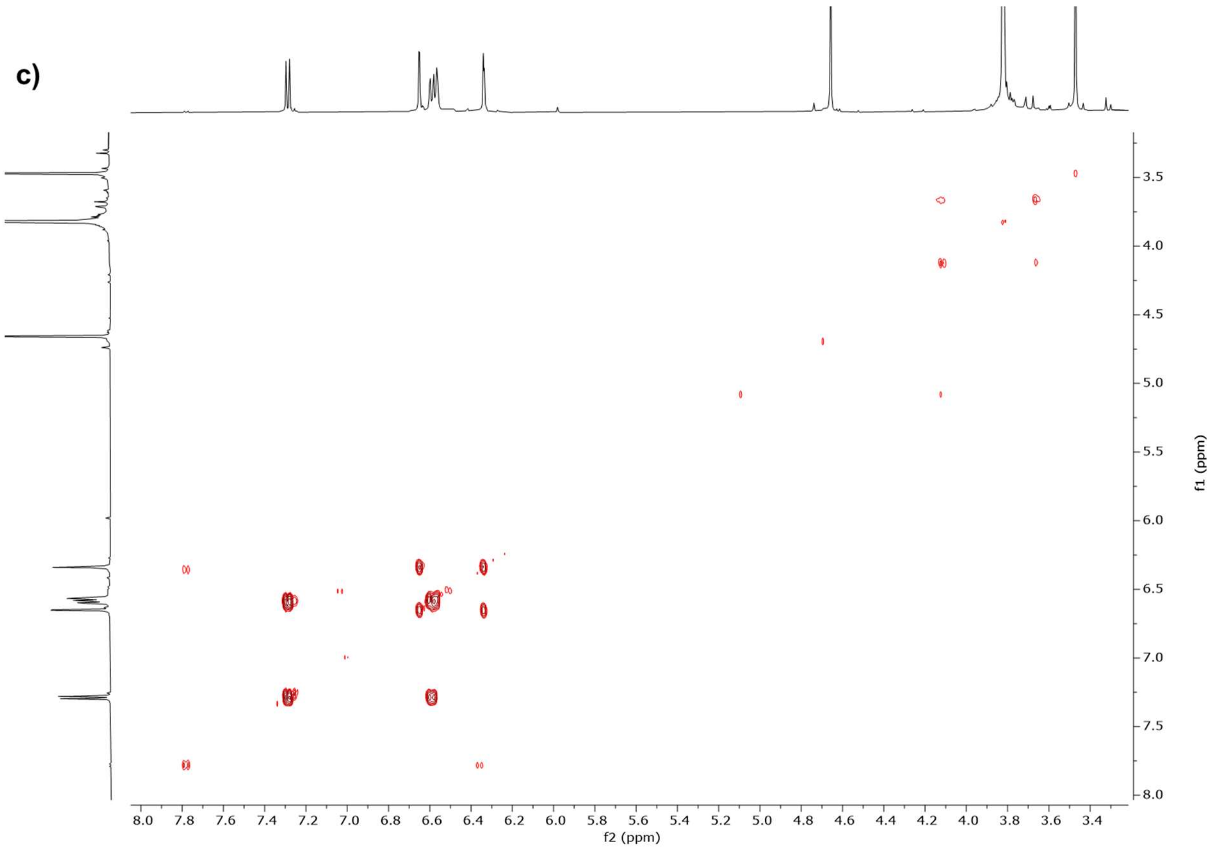
f2 (ppm)
f1 (ppm)

d)

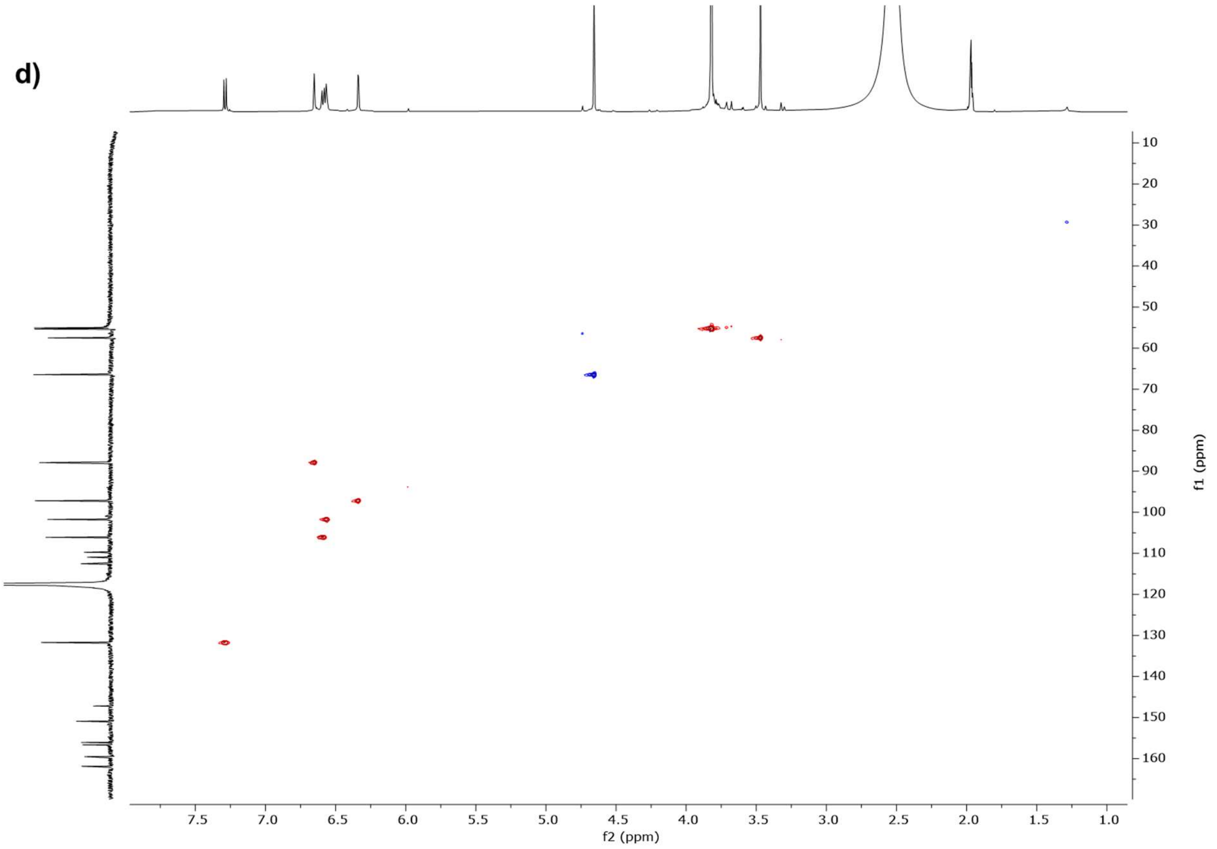

e)

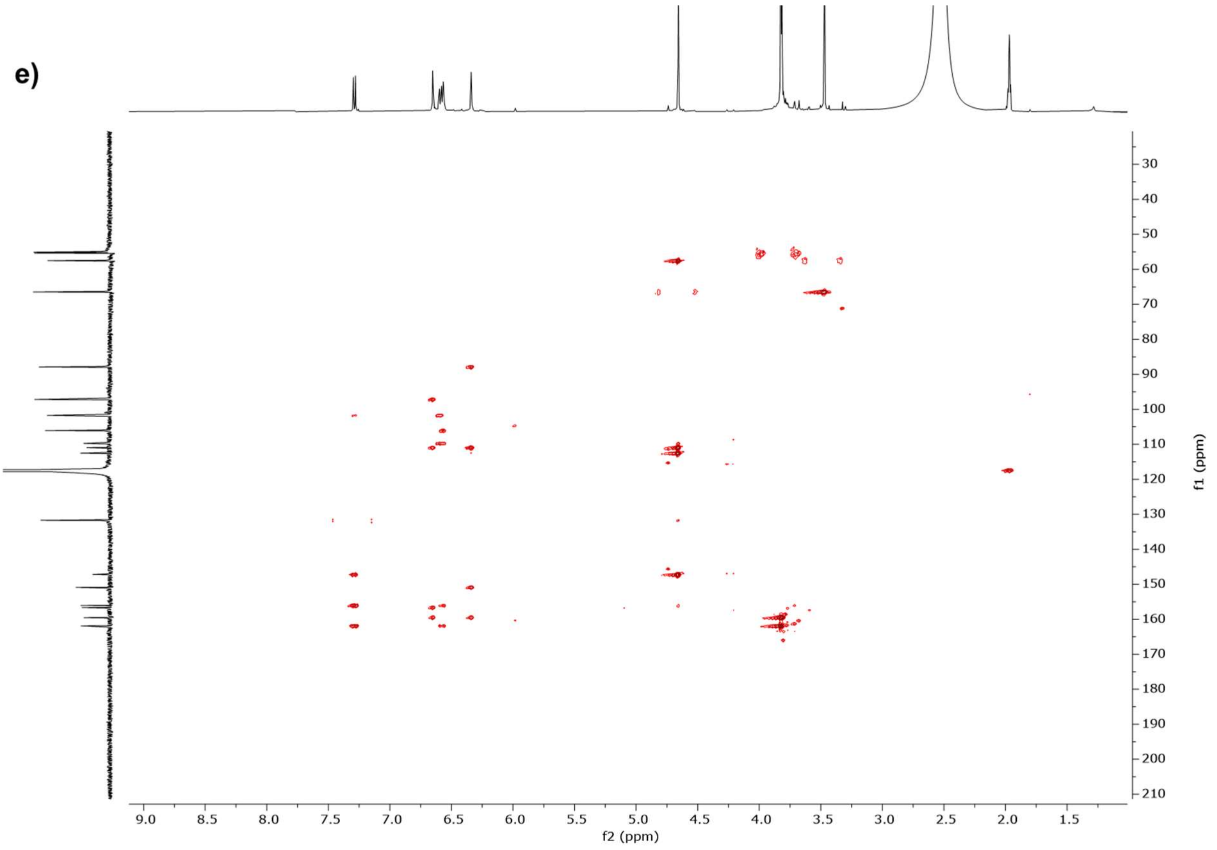

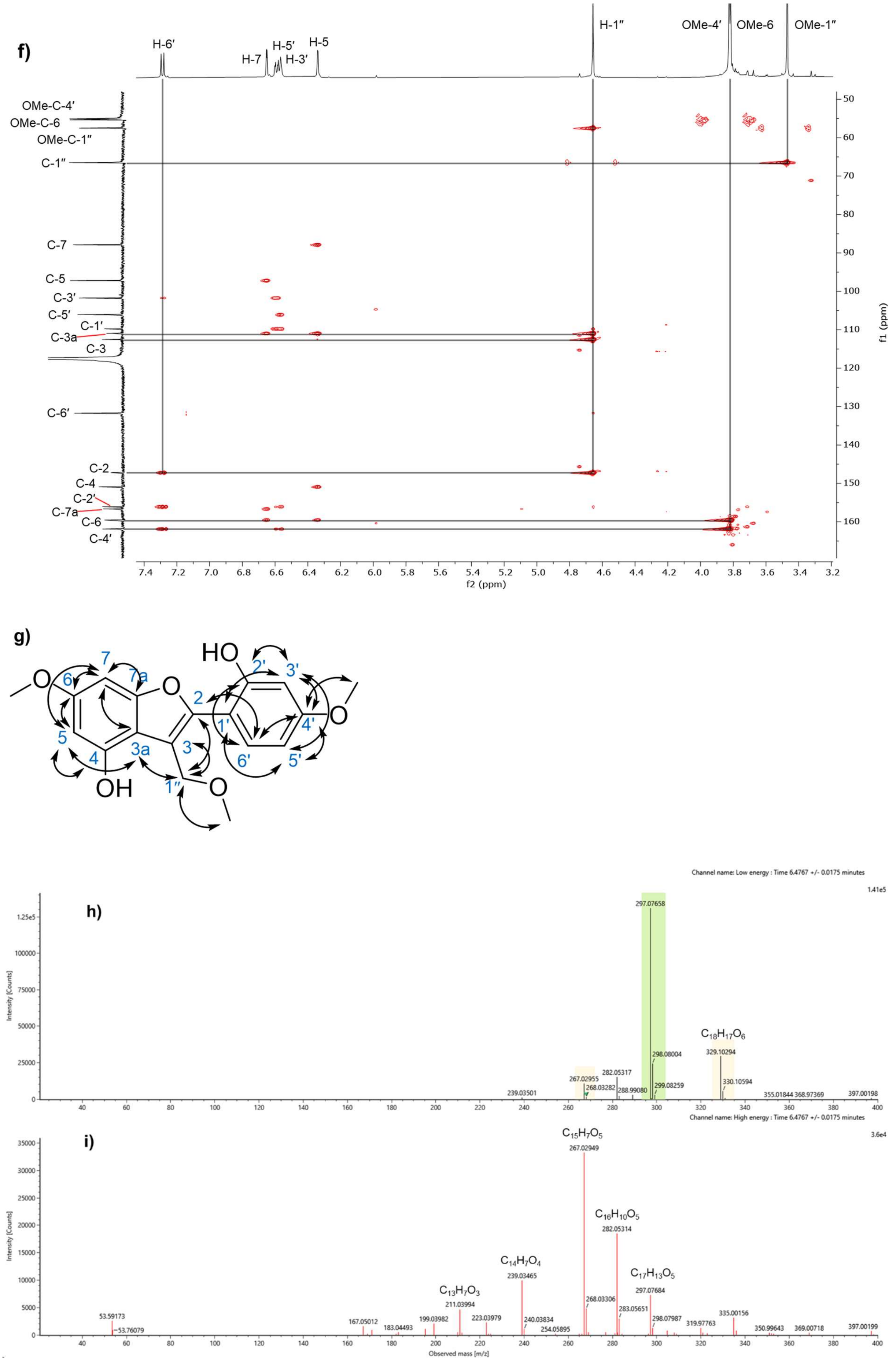
f)
H-6′
H-7
H-5′
H-3′
H-5
H-1″
OMe-4′
OMe-6
OMe-1″
OMe-C-4′
OMe-C-6
OMe-C-1″
C-1″
C-7
C-5
C-3′
C-5′
C-1′
C-3a
C-3
C-6′
C-2
C-4
C-2′
C-7a
C-6
C-4′
f2 (ppm)
f1 (ppm)
g)
HO
OH
O
h)
Channel name: Low energy : Time 6.4767 +/- 0.0175 minutes
1.41e5
297.07658
C18H17O6
329.10294
298.08004
282.05317
267.02955
268.03282
288.99080
299.08259
330.10594
239.03501
355.01844
368.97369
397.00198
Intensity [Counts]
Channel name: High energy : Time 6.4767 +/- 0.0175 minutes
i)
3.6e4
C15H7O5
267.02949
C16H10O5
282.05314
C14H7O4
239.03465
C17H13O5
297.07684
C13H7O3
211.03994
199.03982
223.03979
167.05012
183.04493
240.03834
254.05895
268.03306
283.05651
298.07987
319.97763
335.00156
350.99643
369.00718
397.00199
53.59173
−53.76079
Observed mass [m/z]

Fig. S13: ^{1}H (**a**), ^{13}C (**b**), COSY (**c**), HSQC (**d**), and HMBC (**e** and **f**) NMR spectra ($(CD_3)_2CO$, 600/151 MHz, 300 K) and low- (**g**) and high-energy (**h**) ToF-MS^{e} spectra of a dimeric polyphenol artefact (**29**). COSY: Correlation Spectroscopy; HSQC: Heteronuclear Single Quantum Coherence Spectroscopy; HMBC: Heteronuclear Multiple Bond Correlation Spectroscopy; NMR: Nuclear Magnetic Resonance Spectroscopy; $(CD_3)_2CO$: acetone-d$_6$; ToF-MS^{e}: Time-of-flight-Mass Spectrometry with elevated energy.

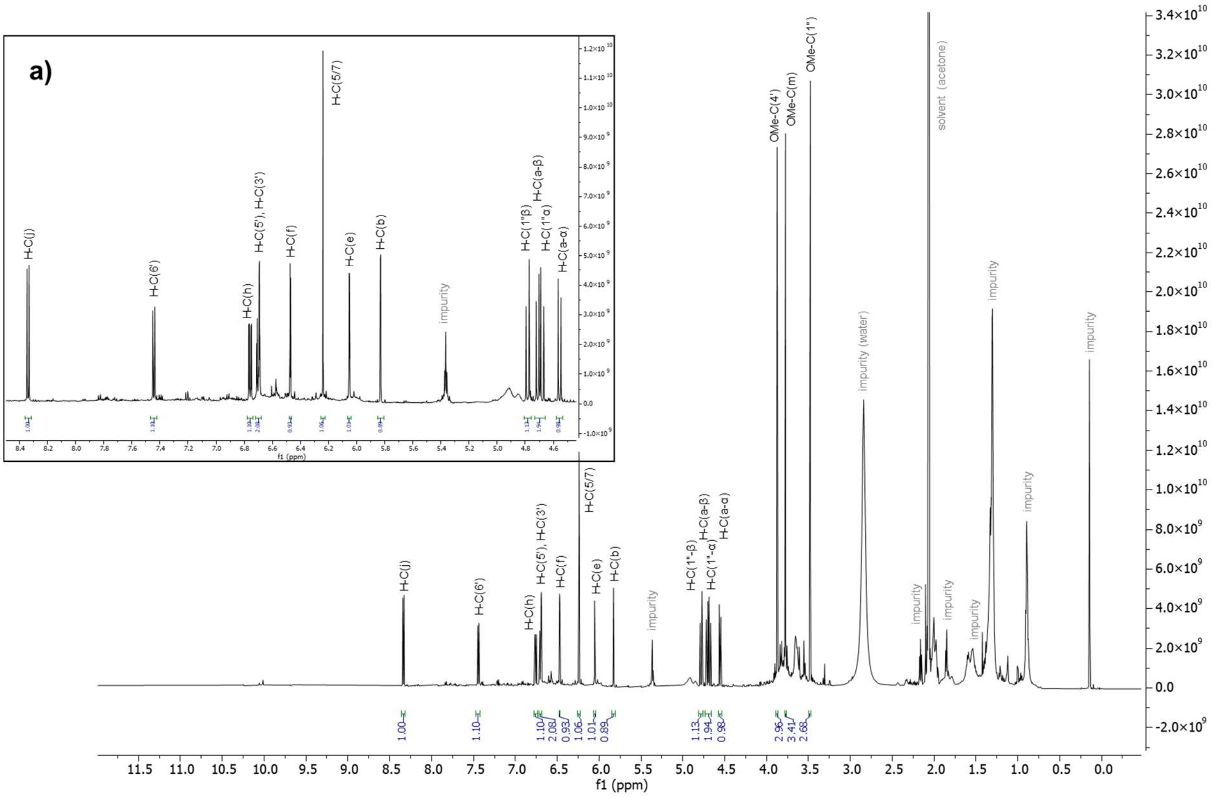

b)

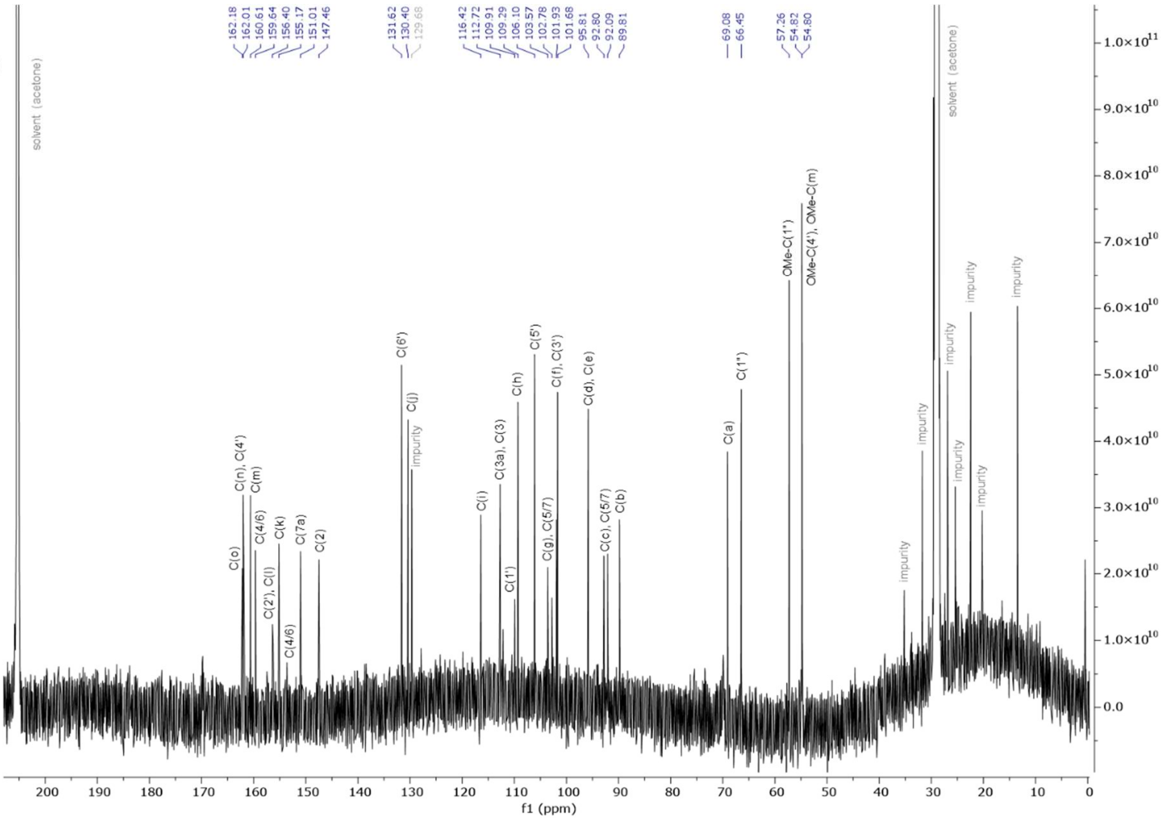

solvent (acetone)
162.18
162.01
160.61
159.64
156.40
155.17
151.01
147.46
131.62
130.40
129.68
116.42
112.72
109.91
109.29
106.10
103.57
102.78
101.93
101.68
95.81
92.80
92.09
89.81
69.08
66.45
57.26
54.82
54.80
C(o)
C(n), C(4')
C(m)
C(2'), C(l)
C(4/6)
C(k)
C(4/6)
C(7a)
C(2)
C(6')
C(j)
impurity
C(i)
C(3a), C(3)
C(1')
C(h)
C(g), C(5/7)
C(5')
C(f), C(3')
C(d), C(e)
C(c), C(5/7)
C(b)
C(a)
C(1")
OMe-C(1")
OMe-C(4'), OMe-C(m)
impurity
solvent (acetone)
1.0×10^11
9.0×10^10
8.0×10^10
7.0×10^10
6.0×10^10
5.0×10^10
4.0×10^10
3.0×10^10
2.0×10^10
1.0×10^10
0.0
f1 (ppm)

c)

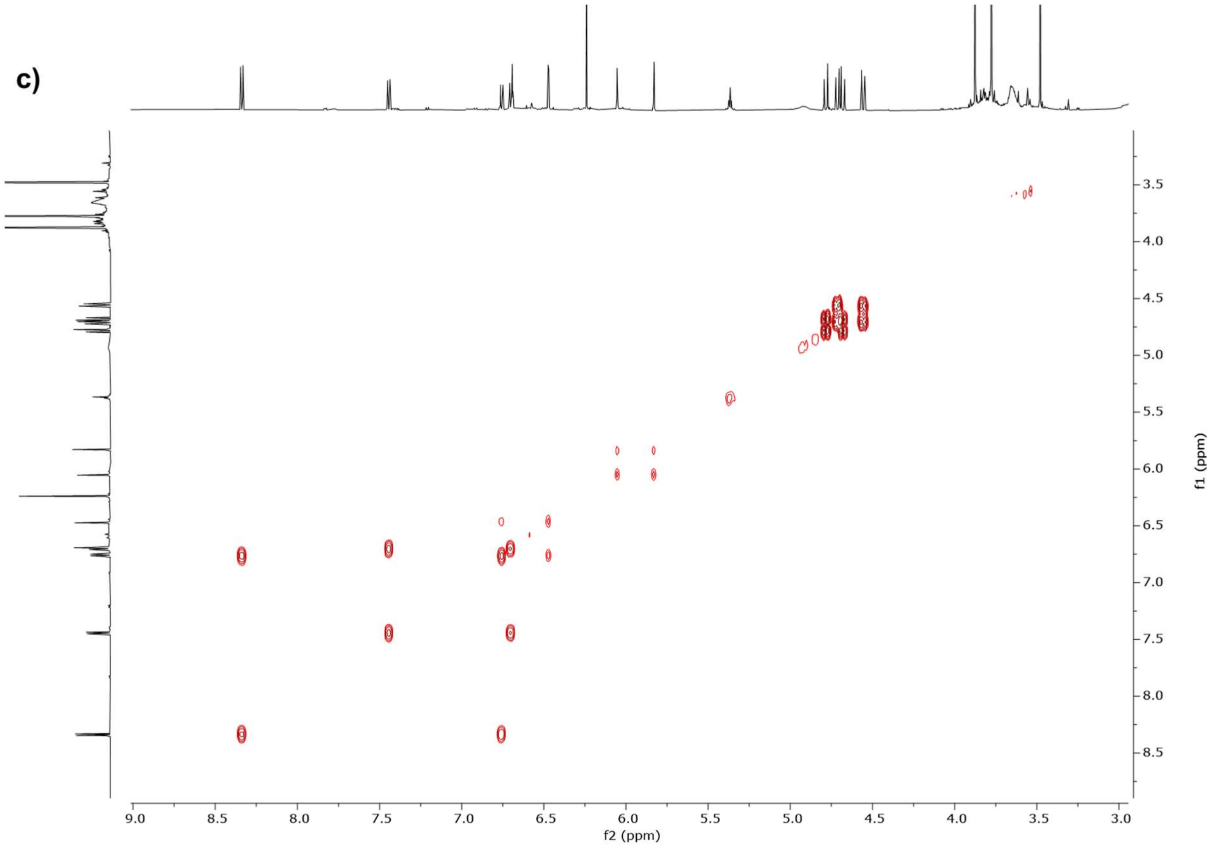

f2 (ppm)
f1 (ppm)

d)

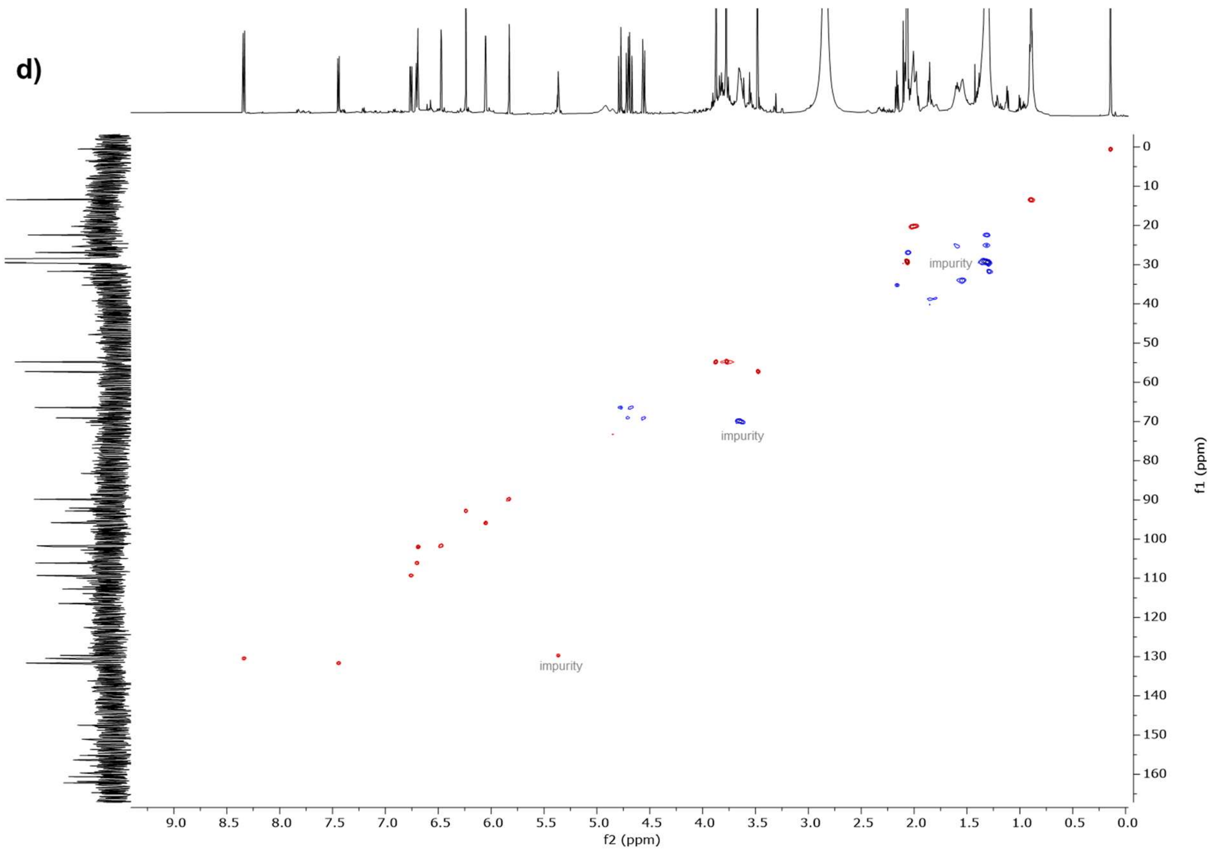
impurity
impurity
impurity
f1 (ppm)
f2 (ppm)

e)

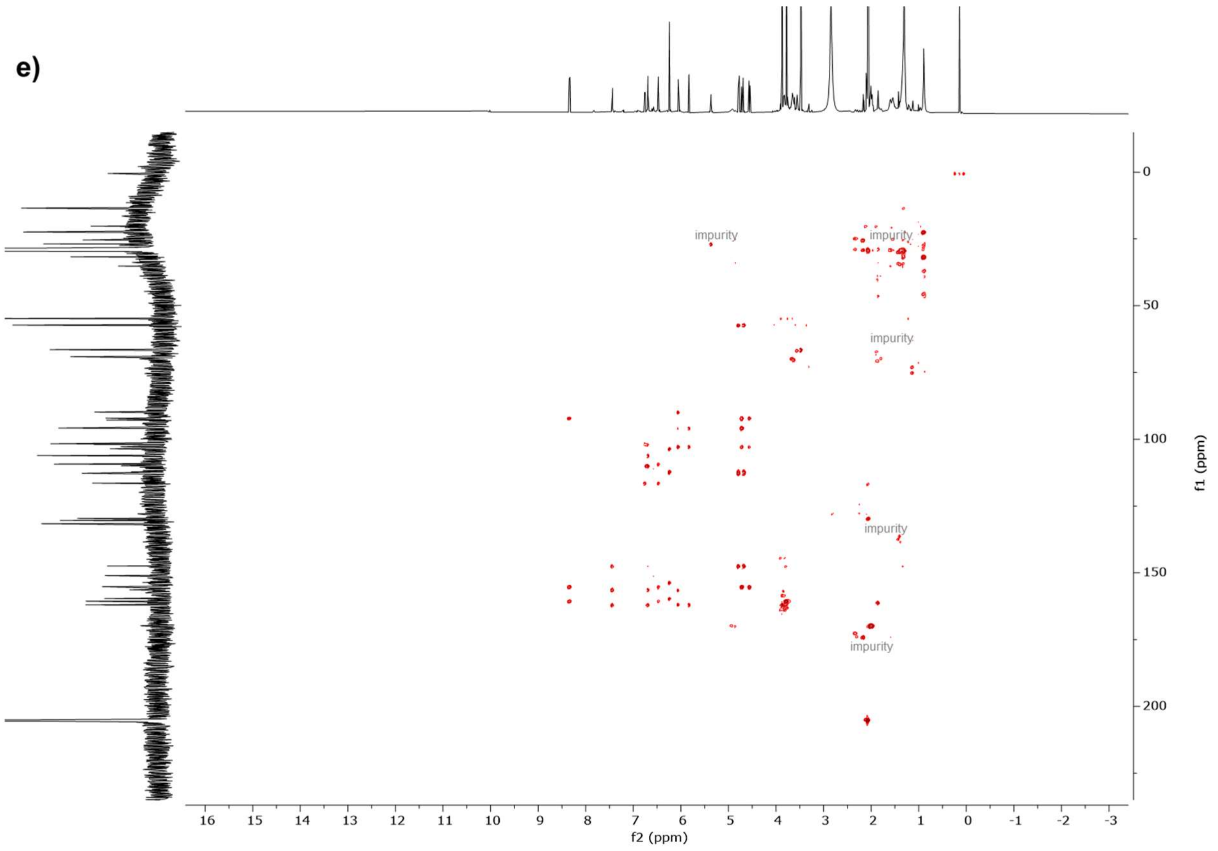
impurity
impurity
impurity
impurity
impurity
f1 (ppm)
f2 (ppm)

f)

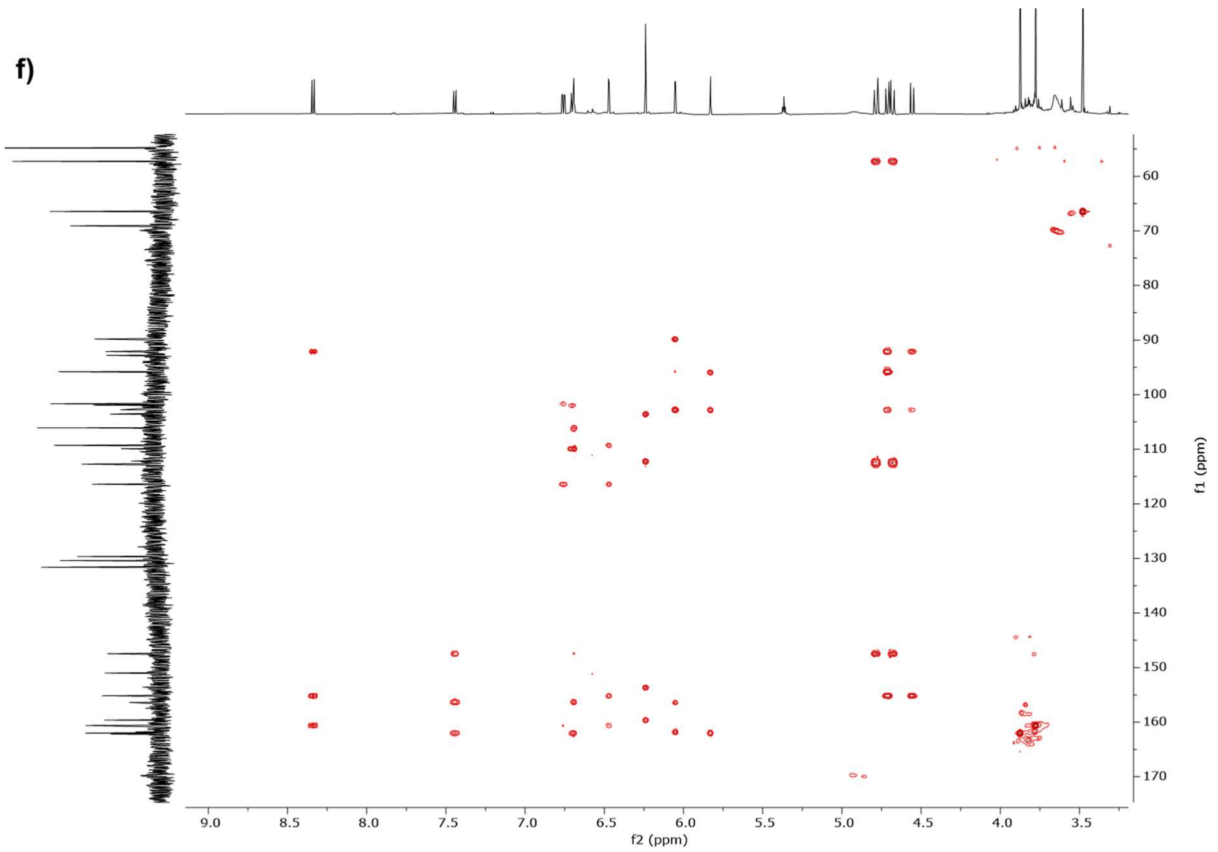
f2 (ppm)
f1 (ppm)

g)

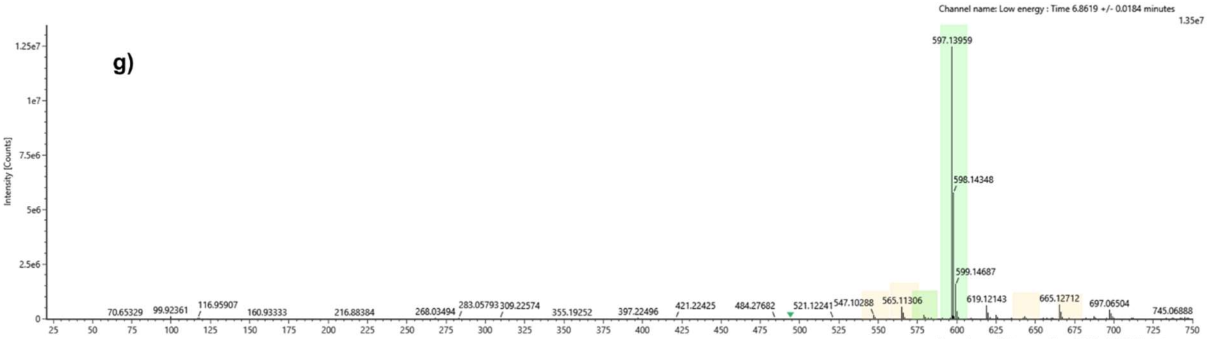
Channel name: Low energy : Time 6.8619 +/- 0.0184 minutes
1.35e7
Intensity [Counts]
597.13959
598.14348
599.14687
619.12143
665.12712
697.06504
745.06888
565.11306
547.10288
521.12241

h)

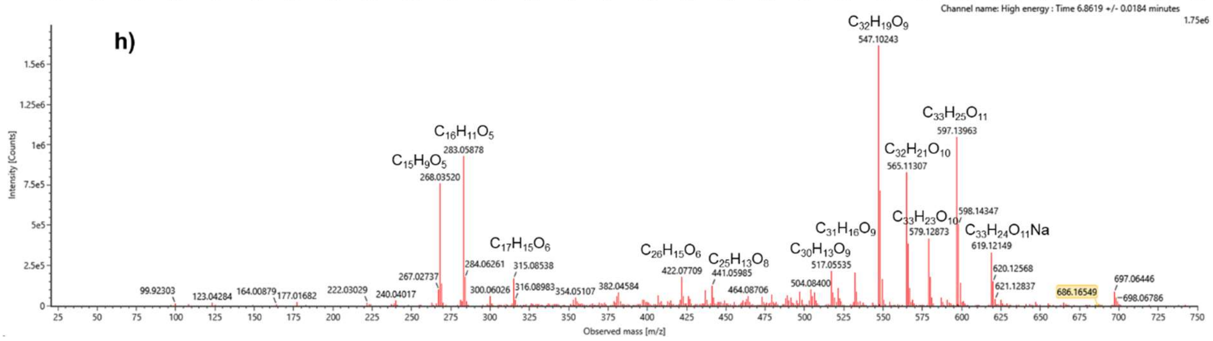
Channel name: High energy : Time 6.8619 +/- 0.0184 minutes
1.75e6
Intensity [Counts]
Observed mass [m/z]
$C_{32}H_{19}O_9$
547.10243
$C_{33}H_{25}O_{11}$
597.13963
$C_{32}H_{21}O_{10}$
565.11307
$C_{33}H_{23}O_{10}$
579.12873
$C_{33}H_{24}O_{11}Na$
619.12149
$C_{16}H_{11}O_5$
283.05878
$C_{15}H_9O_5$
268.03520
$C_{17}H_{15}O_6$
315.08538
$C_{26}H_{15}O_6$
422.07709
$C_{25}H_{13}O_8$
441.05985
$C_{31}H_{16}O_9$
$C_{30}H_{13}O_9$
517.05535

Fig. S14: Structure proposals for the dimeric polyphenol artefact **29**.

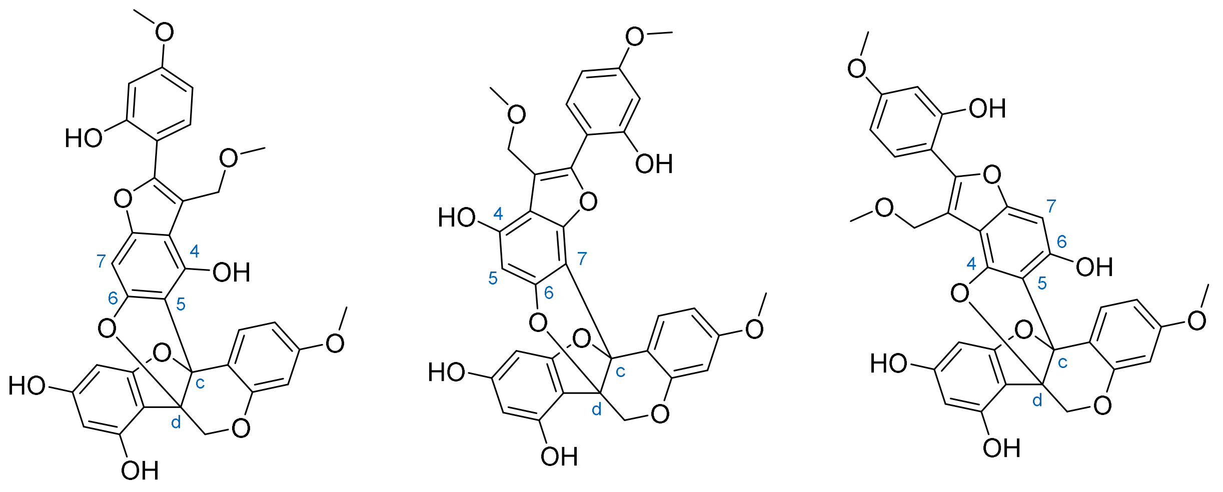

Fig. S15: Low- (**a**) and high-energy (**b**) ToF-MS^e spectra of postulated 4-hydroxy-2-(2′-hydroxy-4′-methoxyphenyl)-6-methoxy-3-(ethoxymethyl)benzofuran (**27**). ToF-MS^e: Time-of-flight-Mass Spectrometry with elevated energy.

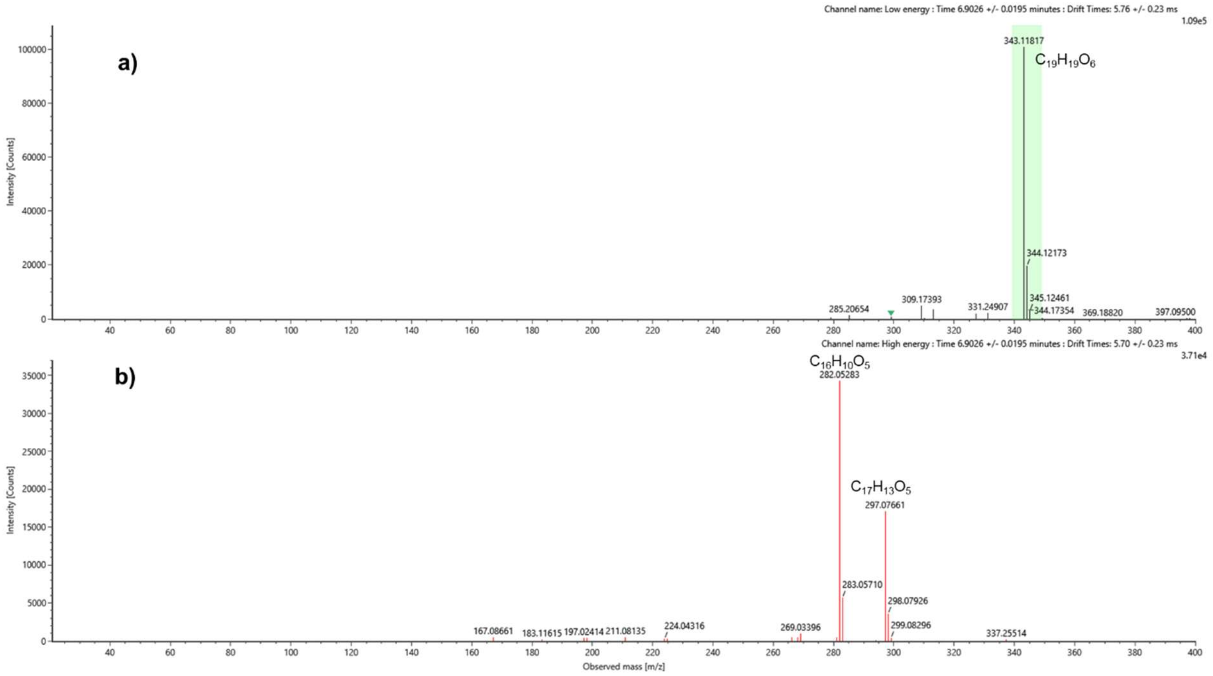

Fig. S16: Normalized response of **a)** lotuscarpene (**16**) and its MeOH adduct (**26**) and postulated EtOH adduct (**27**), **b)** postulated **28** and its MeOH adduct **25**, **c)** postulated dimeric polyphenol **30** and its MeOH adduct **29**, **d)** postulated pterocarp-6a-enes **1** and **10**, and **e)** lotusaldehyde (**15**) and postulated benzofuran carbaldehyde **9** in MeOH (light green), EtOH (dark green) and MeCN (blue) after 0, 24, 48, and 72 h of UV light exposure. MeOH: methanol; EtOH: ethanol, MeCN: acetonitrile.

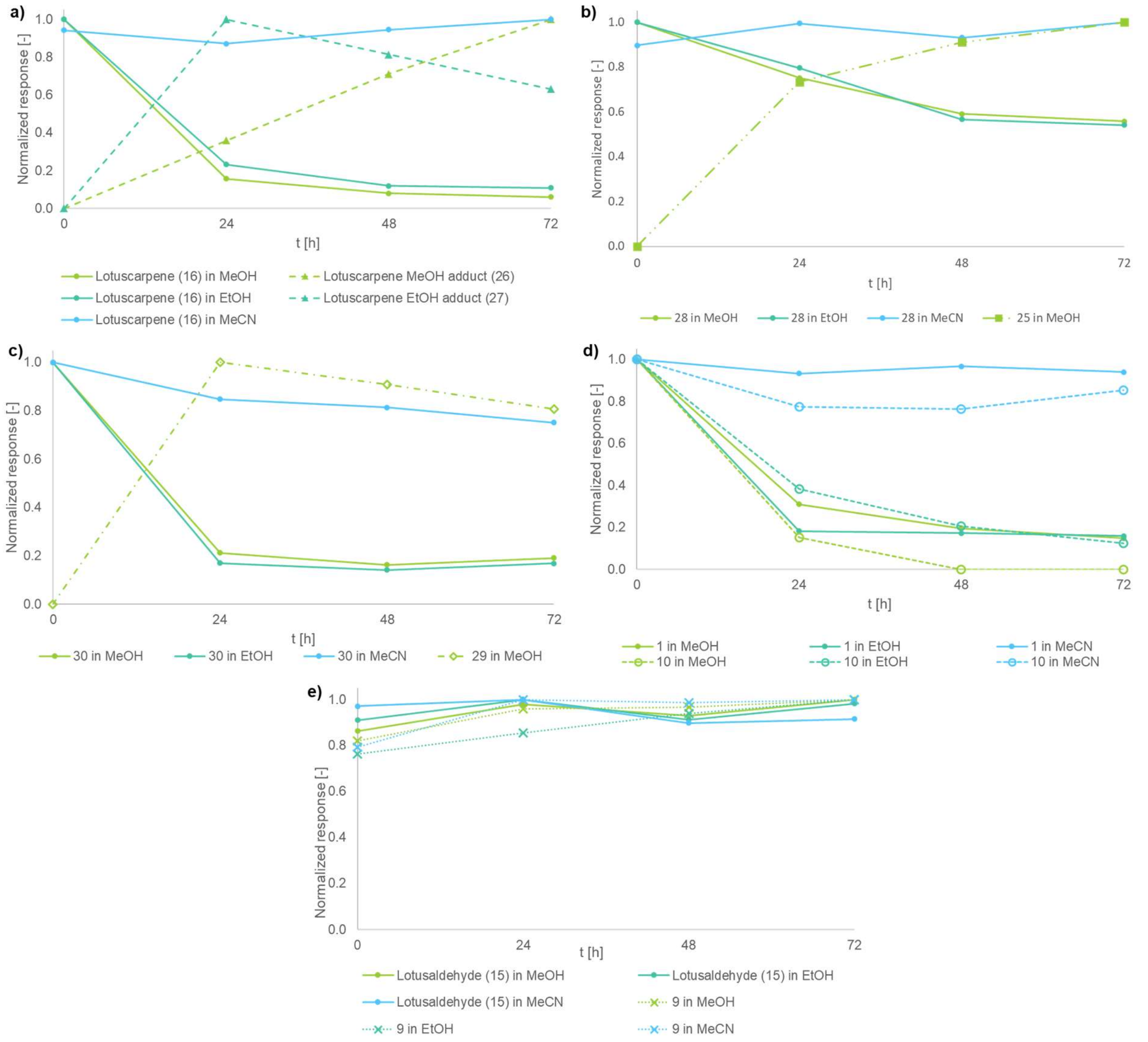

Fig. S17: Low- (**a**) and high-energy (**b**) ToF-MS^e spectra of postulated 7,9-dihydroxy-3-methoxypterocarp-6a-ene (**28**). ToF-MS^e: Time-of-flight-Mass Spectrometry with elevated energy.

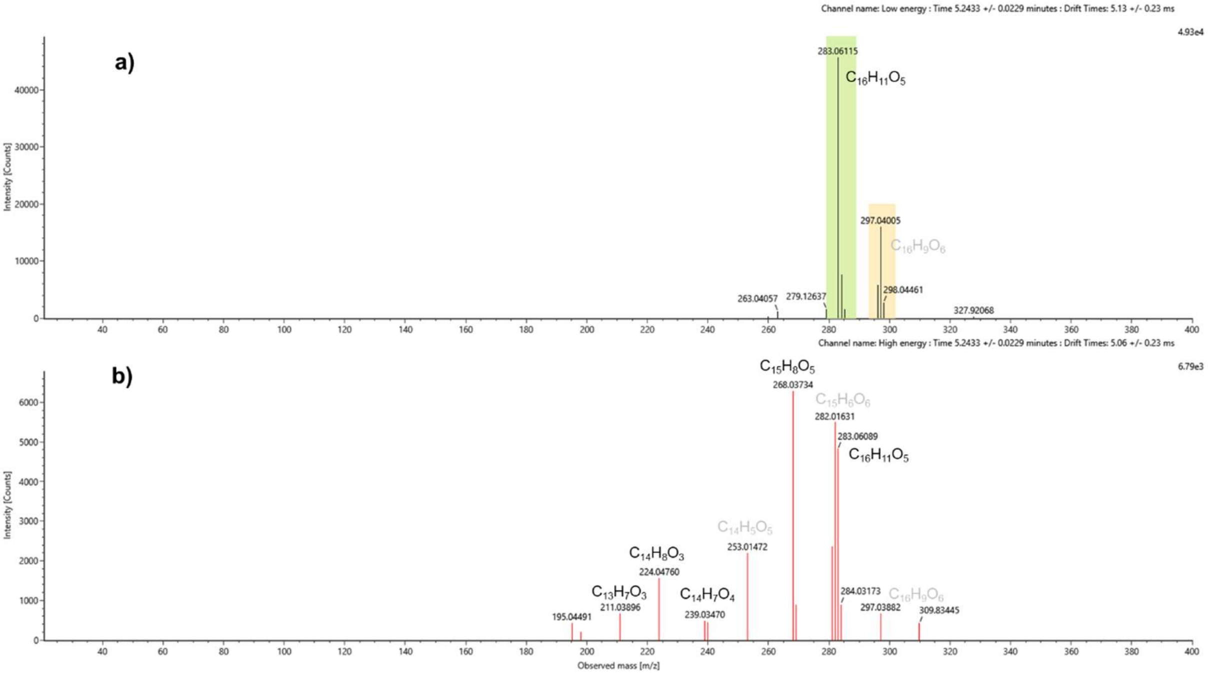

Fig. S18: Low- (**a**) and high-energy (**b**) ToF-MS^e spectra of postulated dimeric polyphenol **30**, a precursor to isolated artefact **29**. ToF-MS^e: Time-of-flight-Mass Spectrometry with elevated energy.

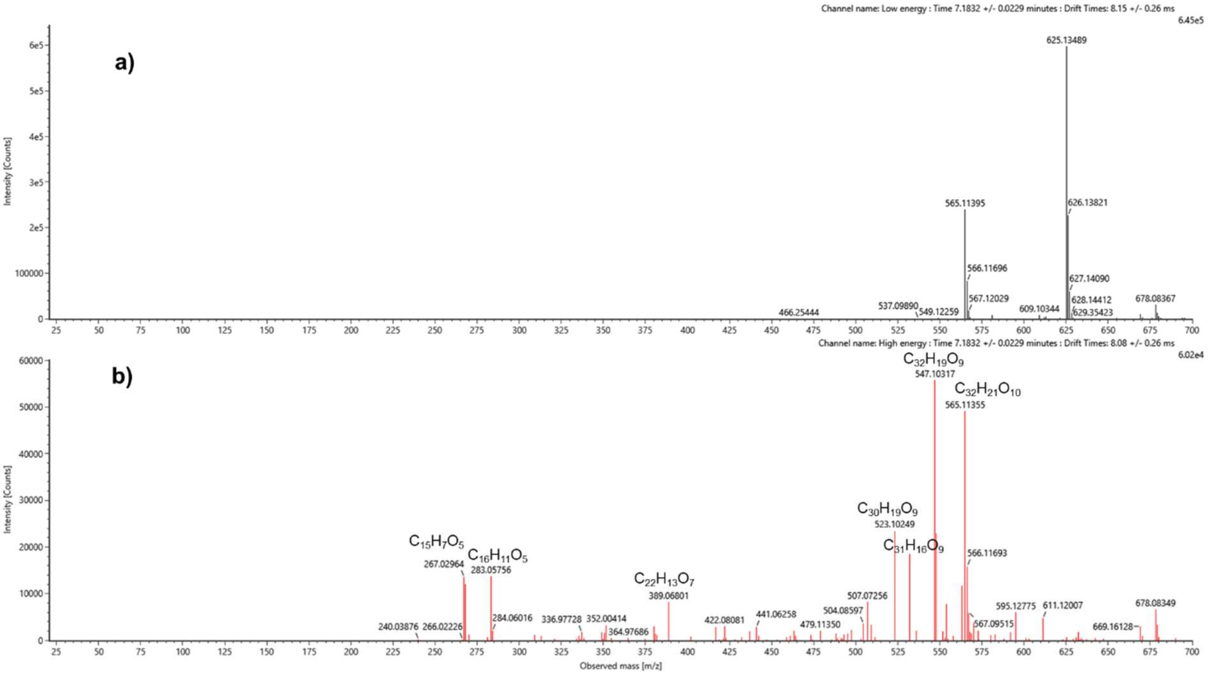

Fig. S19: Low- (**a**) and high-energy (**b**) ToF-MS^e spectra of a postulated hydroxy dimethoxypterocarp-6a-ene (**1**). ToF-MS^e: Time-of-flight-Mass Spectrometry with elevated energy.

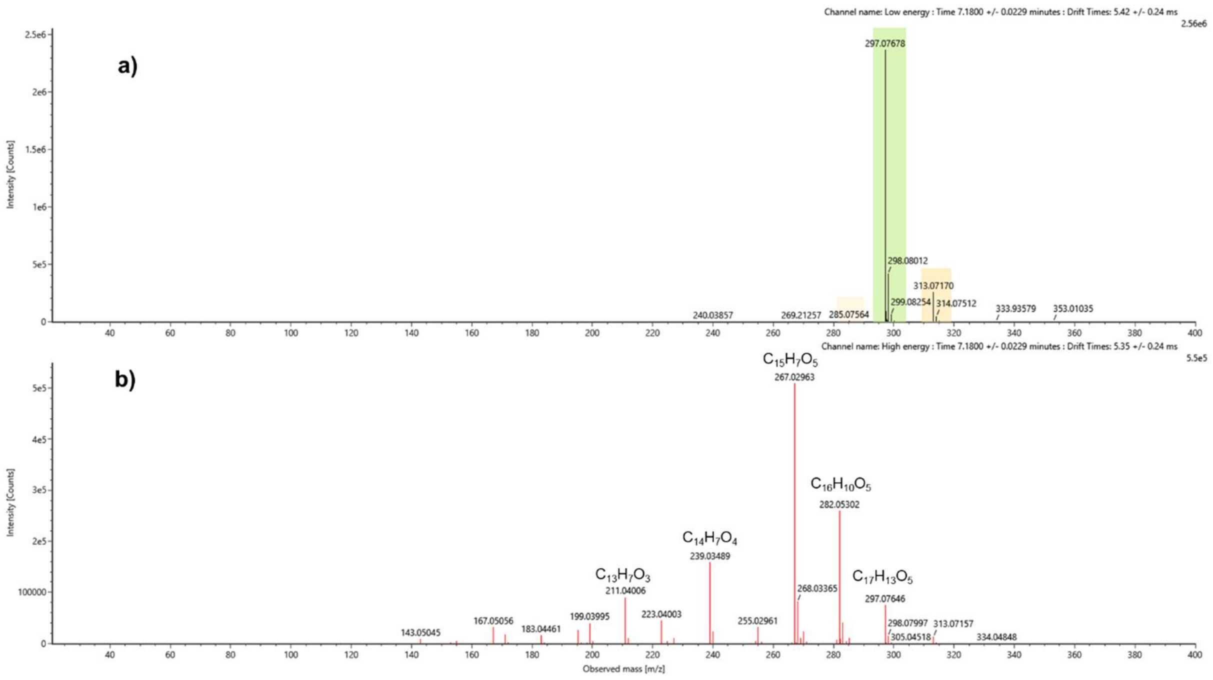

Fig. S20: Low- (**a**) and high-energy (**b**) ToF-MS^e spectra of a postulated dihydroxy trimethoxy aryl benzofuran-3-carbaldehyde (**9**). ToF-MS^e: Time-of-flight-Mass Spectrometry with elevated energy.

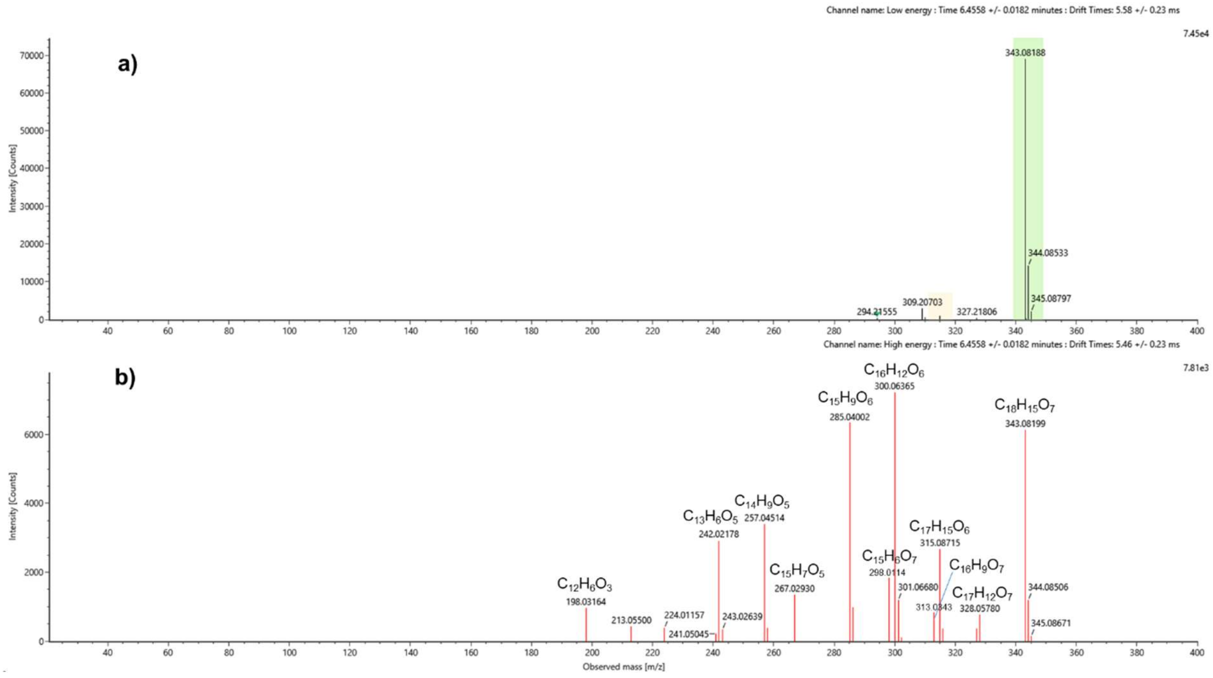

Fig. S21: Low- (**a**) and high-energy (**b**) ToF-MS^e spectra of a postulated hydroxy trimethoxypterocarp-6a-ene (**10**). ToF-MS^e: Time-of-flight-Mass Spectrometry with elevated energy.

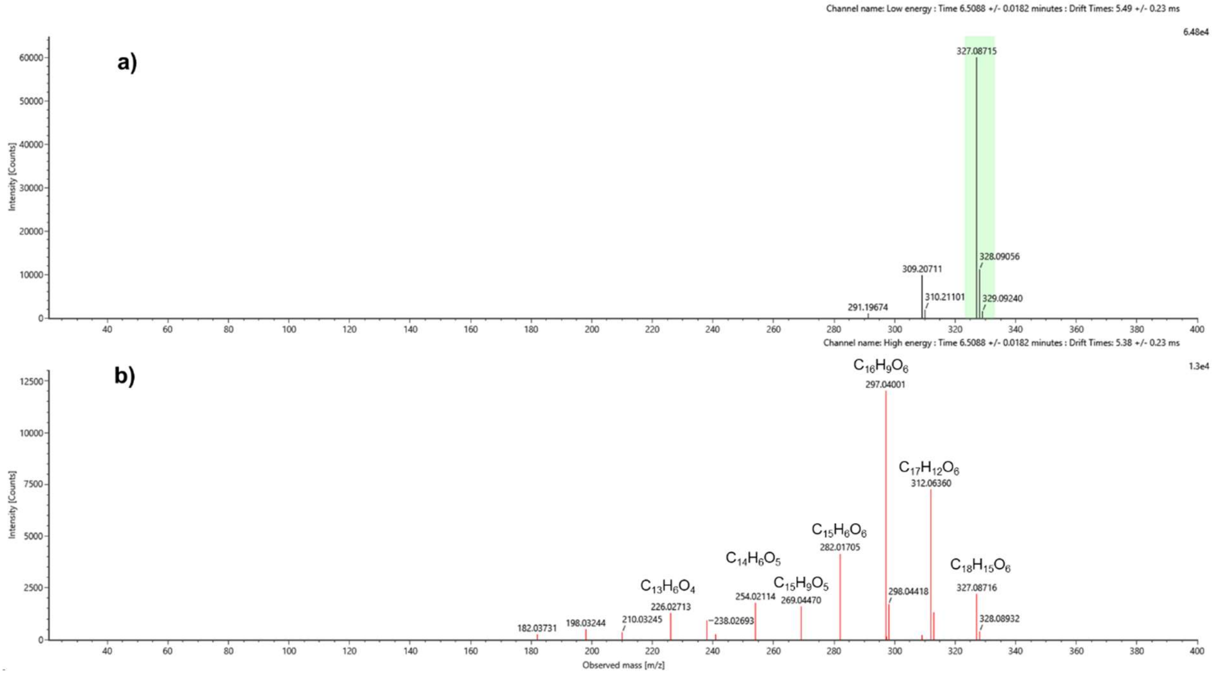

Fig. S22: Low- (**a**) and high-energy (**b**) ToF-MS^e spectra of a postulated 7-hydroxy-3,9-dimethoxycoumestan (**11**). ToF-MS^e: Time-of-flight-Mass Spectrometry with elevated energy.

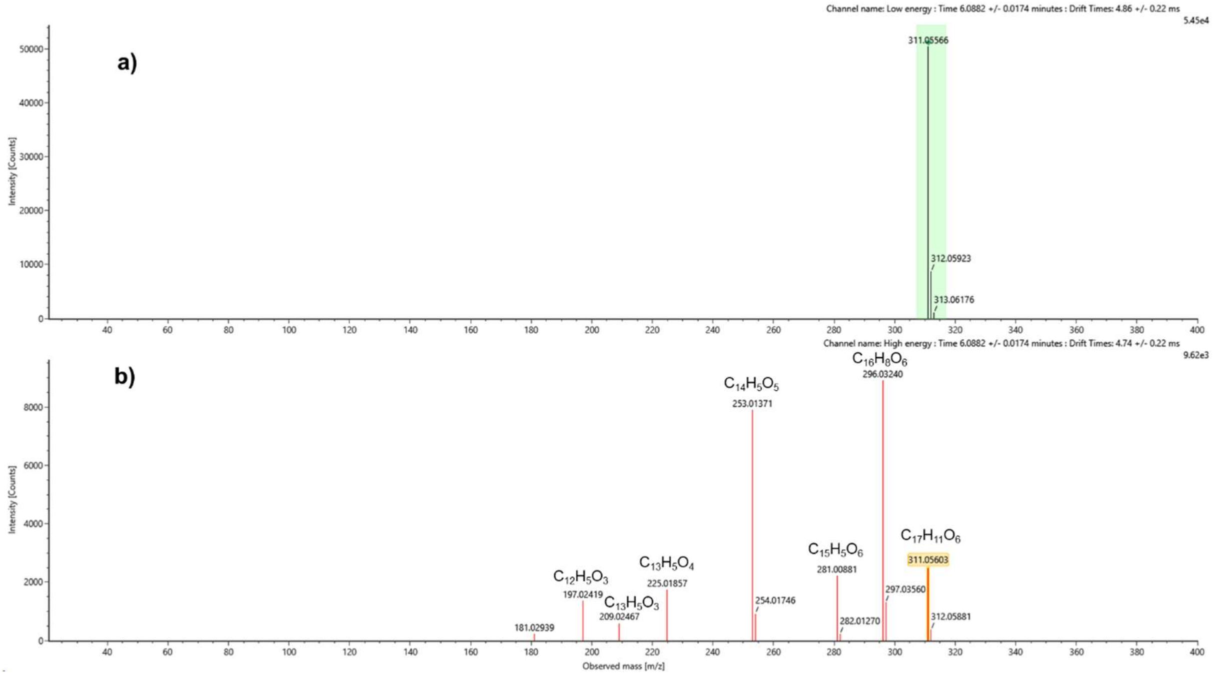

Fig. S23: Mean of semi-quantitative normalized abundances across 3 biological replicates and 3 technical replicates each of **a)** compound **1** in non-inoculated (control) and inoculated (arbuscular mycorrhiza, AM) *Lotus japonicus* wild type and mutant (*ccamk-3*, *ccamk-13*, *cyclops-3*, *cyclops-4*, *ram1-3*, *ram1-4*, *ram2-1*, *ram2-2*) roots harvested at 7 weeks post inoculation (wpi), **b)** lupinalbin B (**3**), **c)** lotuschromone (**13**), **d)** lotusaldehyde (**15**), and **e)** lotuscarpene (**16**) in control and AM wild-type and mutant roots at 7 wpi

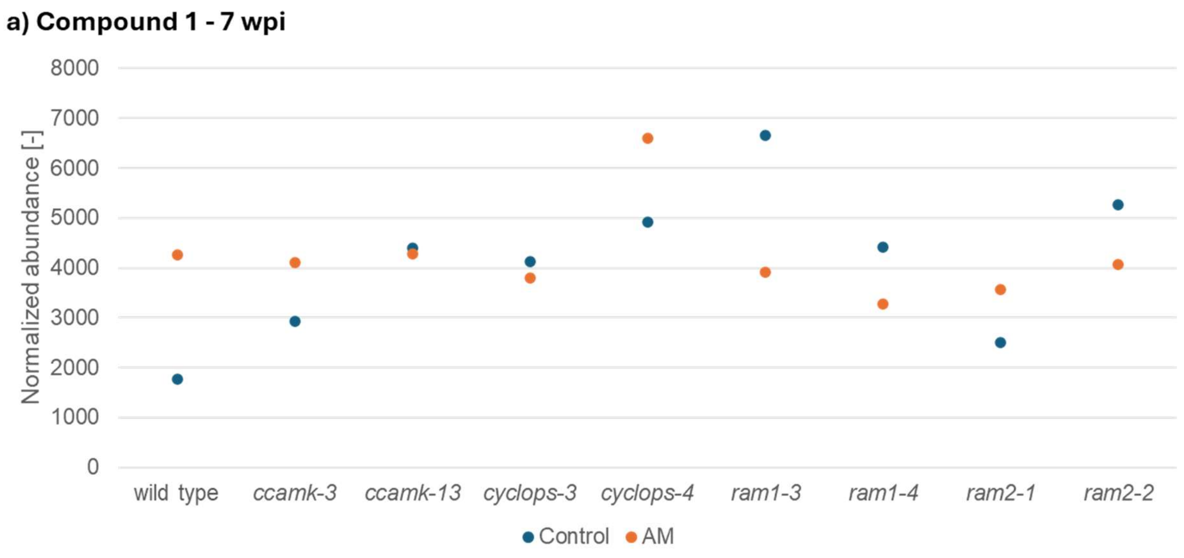

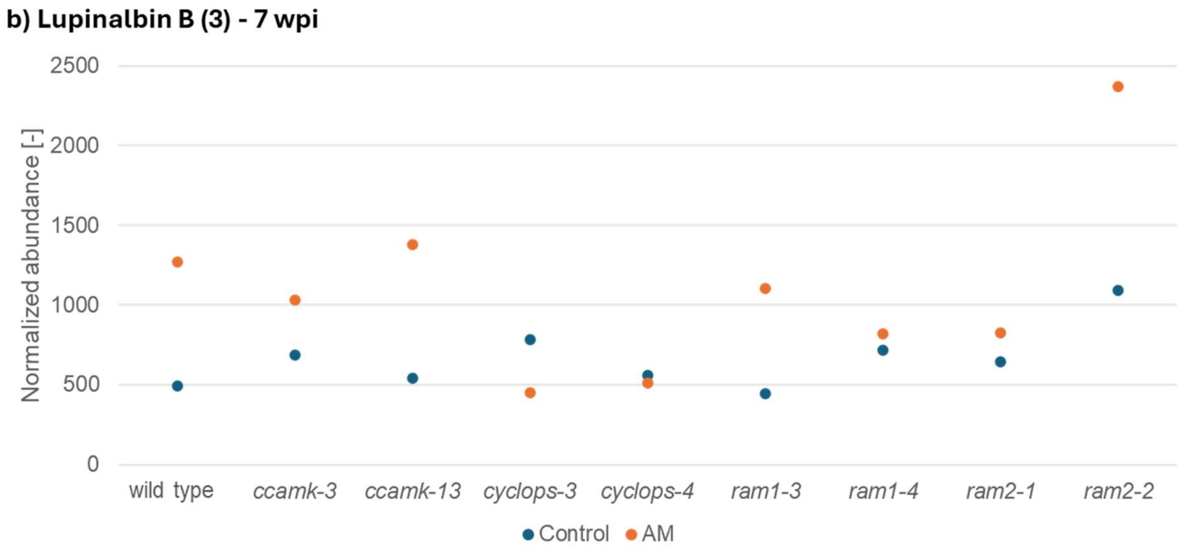

c) Lotuschromone (13) - 7 wpi

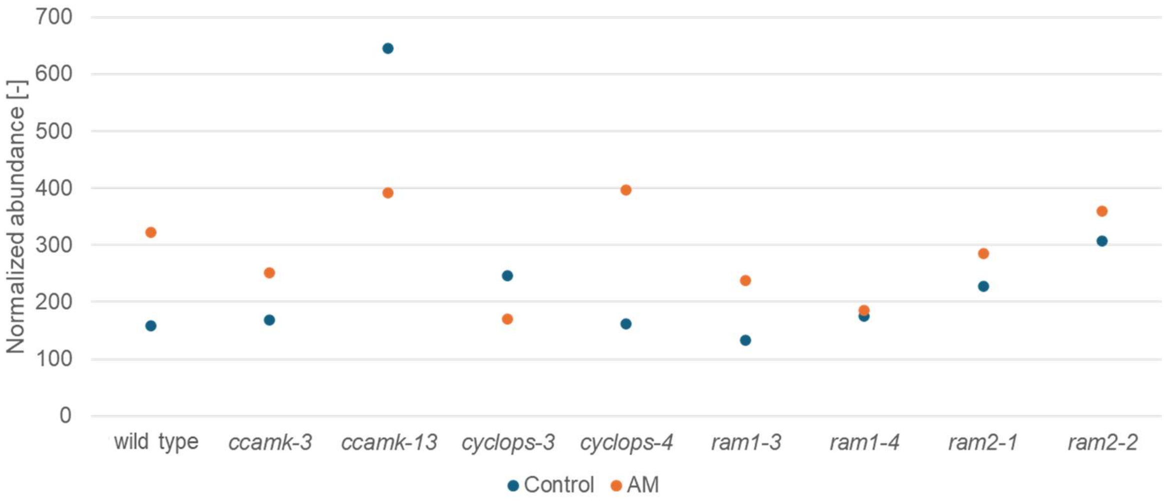

d) Lotusaldehyde (15) - 7 wpi

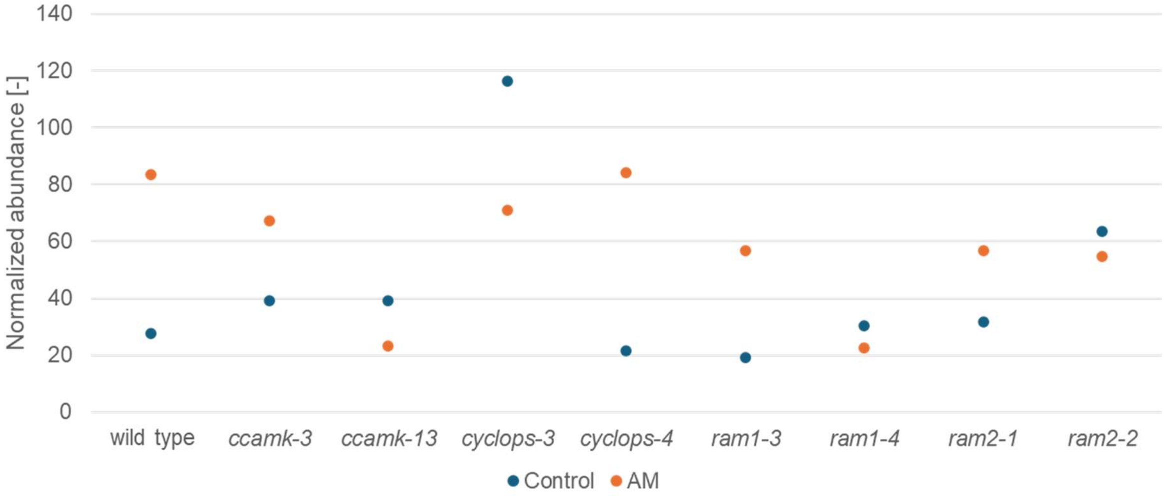

e) Lotuscarpene (16) - 7 wpi

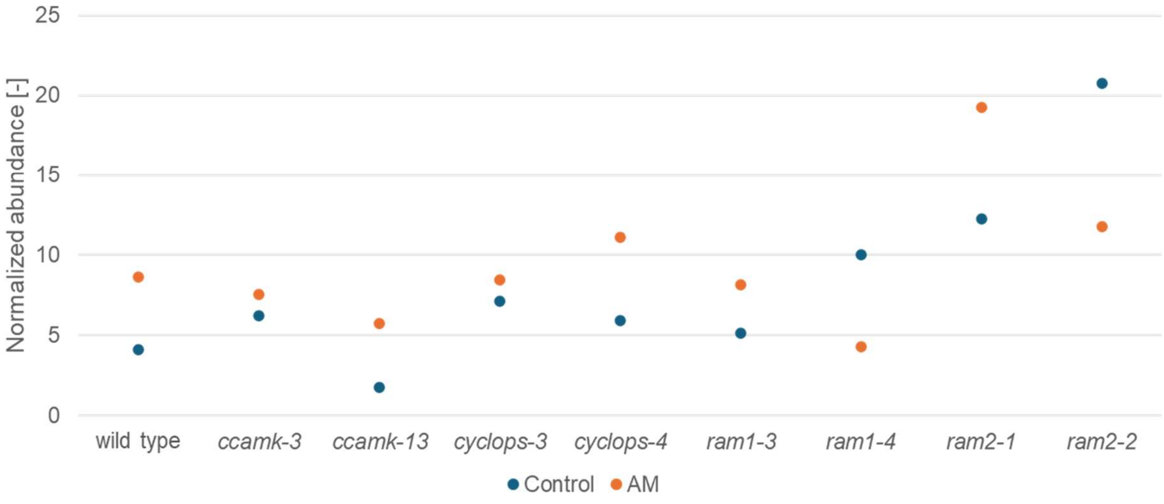

Fig. S24: Mean of semi-quantitative normalized abundances across 3 biological replicates and 3 technical replicates each of **a)** unidentified feature *m/z* 681.1050 (retention time 7.26 min) in non-inoculated (control) and inoculated (arbuscular mycorrhiza, AM) *Lotus japonicus* wild type and mutant (*ccamk-3*, *ccamk-13*, *cyclops-3*, *cyclops-4*, *ram1-3*, *ram1-4*, *ram2-1*, *ram2-2*) roots harvested at 7 weeks post inoculation (7 wpi), and **b)** unidentified feature *m/z* 710.1082 (retention time 7.25 min) in control and AM wild-type and mutant roots harvested at 7 wpi.

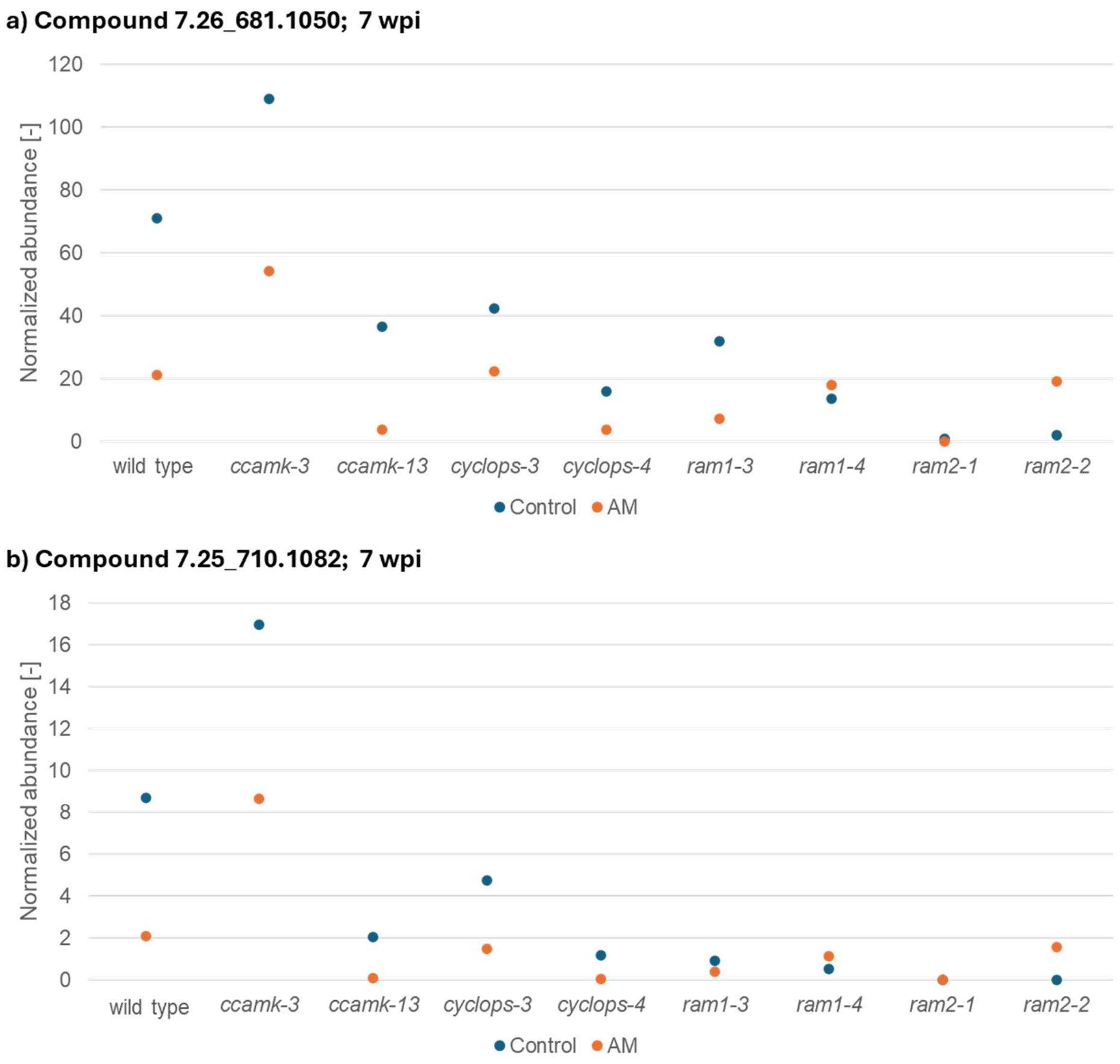

Fig. S25: Low- (**a**) and high-energy (**b**) ToF-MS^{e} spectra of compound **2**. ToF-MS^{e}: Time-of-flight-Mass Spectrometry with elevated energy.

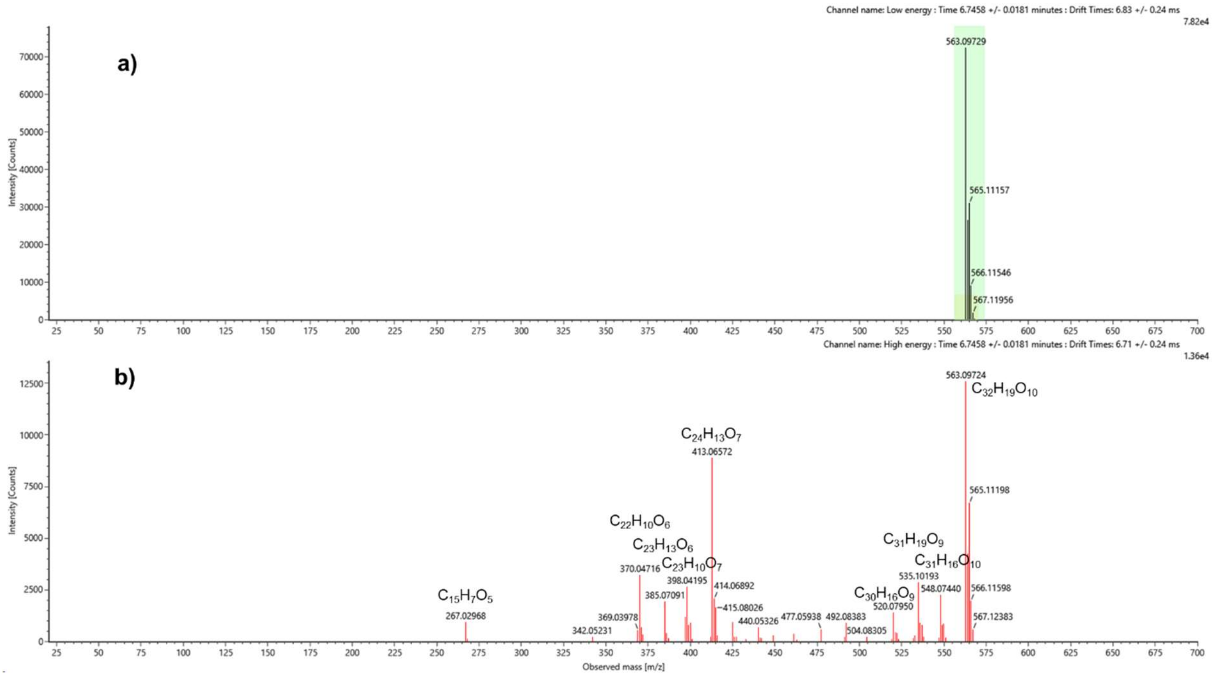

Fig. S26: Low- (**a**) and high-energy (**b**) ToF-MS^{e} spectra of compound **5**. ToF-MS^{e}: Time-of-flight-Mass Spectrometry with elevated energy.

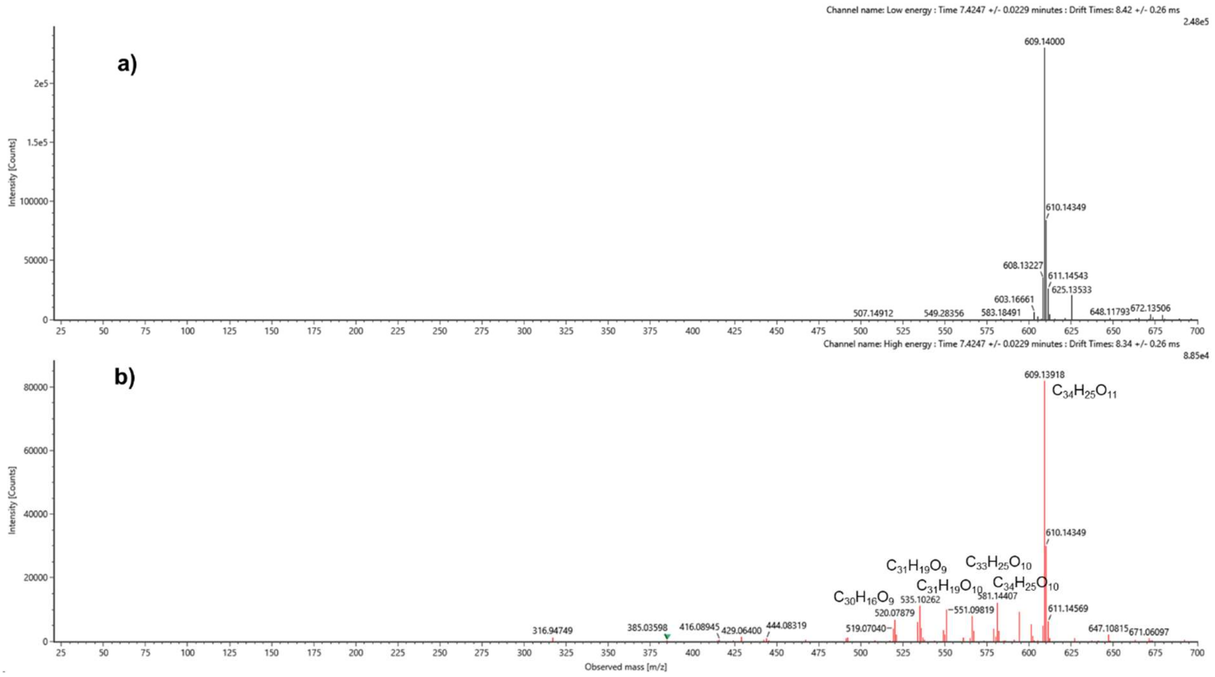

Fig. S27: Low- (**a**) and high-energy (**b**) ToF-MS^e spectra of compound **6**. ToF-MS^e: Time-of-flight-Mass Spectrometry with elevated energy.

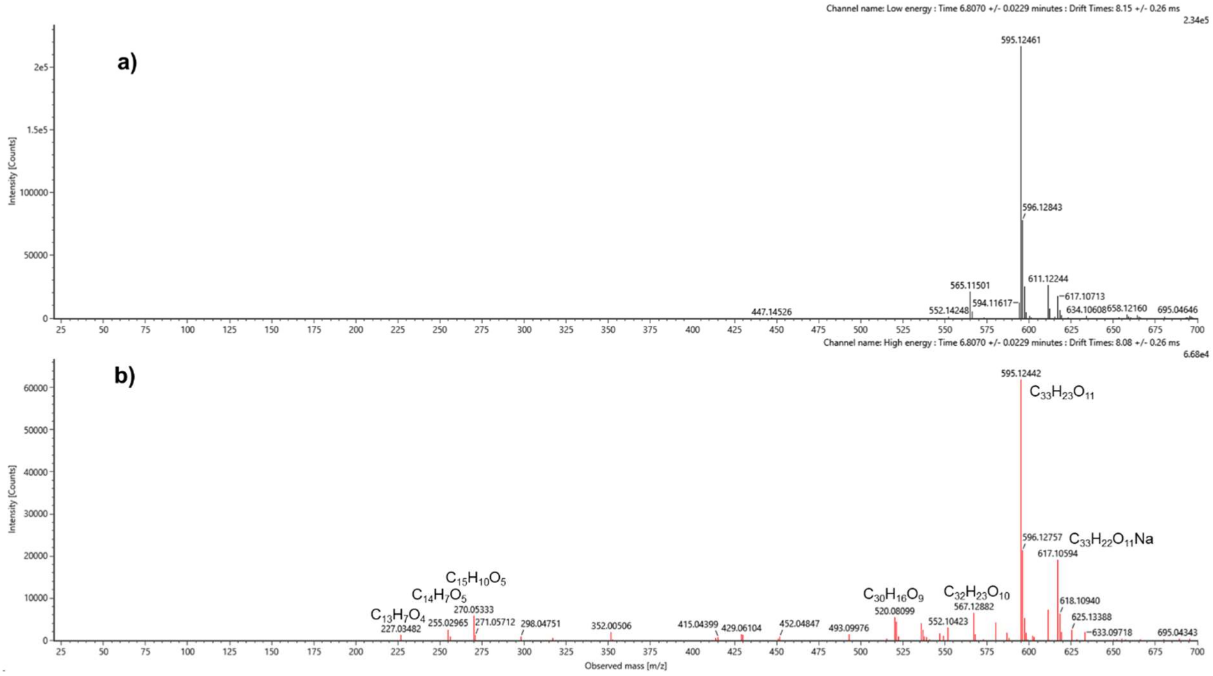

Fig. S28: Low- (**a**) and high-energy (**b**) ToF-MS^e spectra of compound **17**. ToF-MS^e: Time-of-flight-Mass Spectrometry with elevated energy.

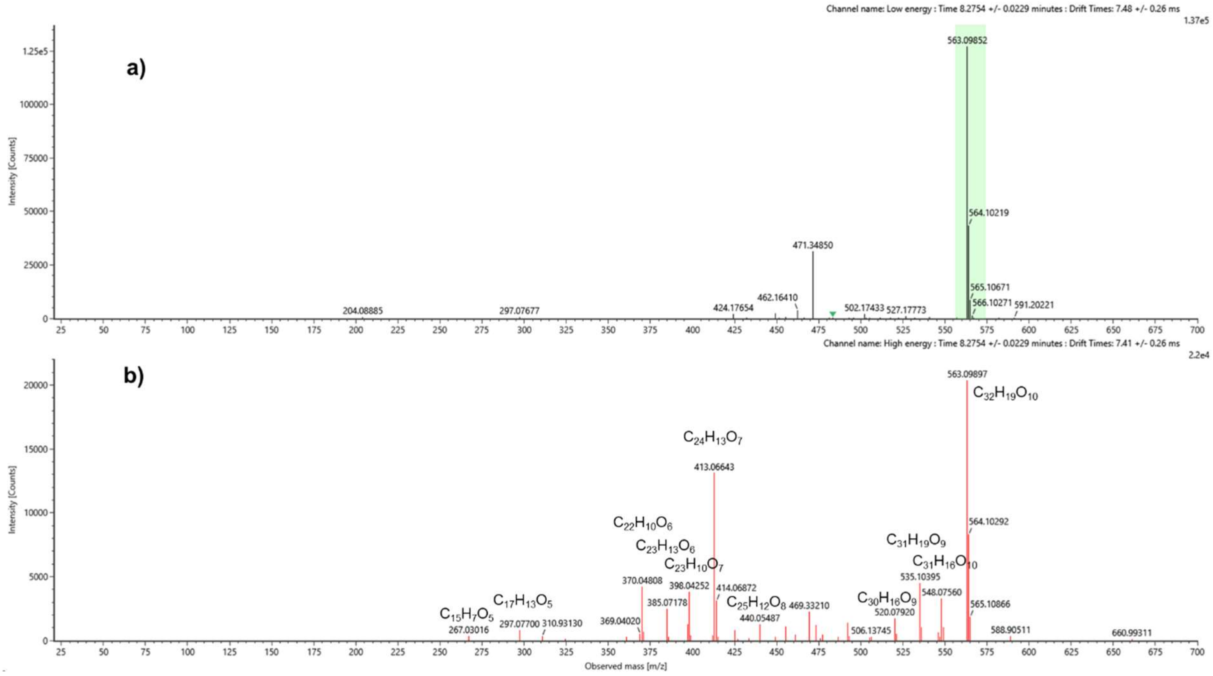

Fig. S29: Low- (**a**) and high-energy (**b**) ToF-MS^e spectra of compound **18**. ToF-MS^e: Time-of-flight-Mass Spectrometry with elevated energy.

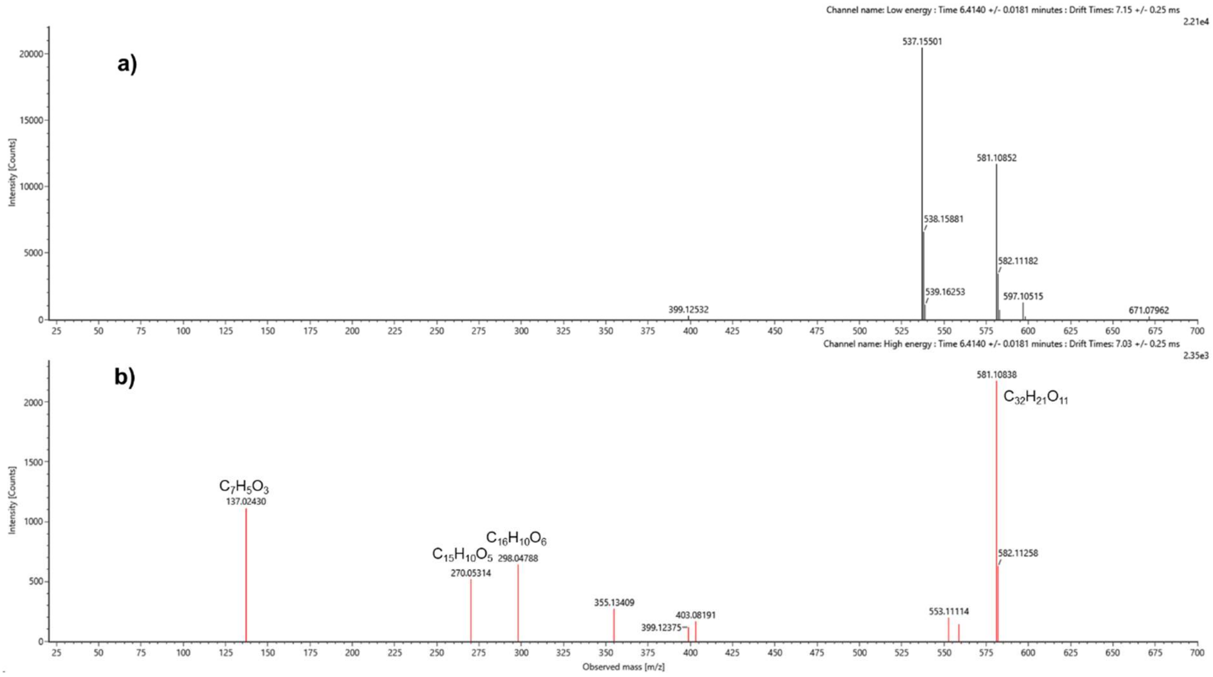

Fig. S30: Low- (**a**) and high-energy (**b**) ToF-MS^e spectra of compound **19**. ToF-MS^e: Time-of-flight-Mass Spectrometry with elevated energy.

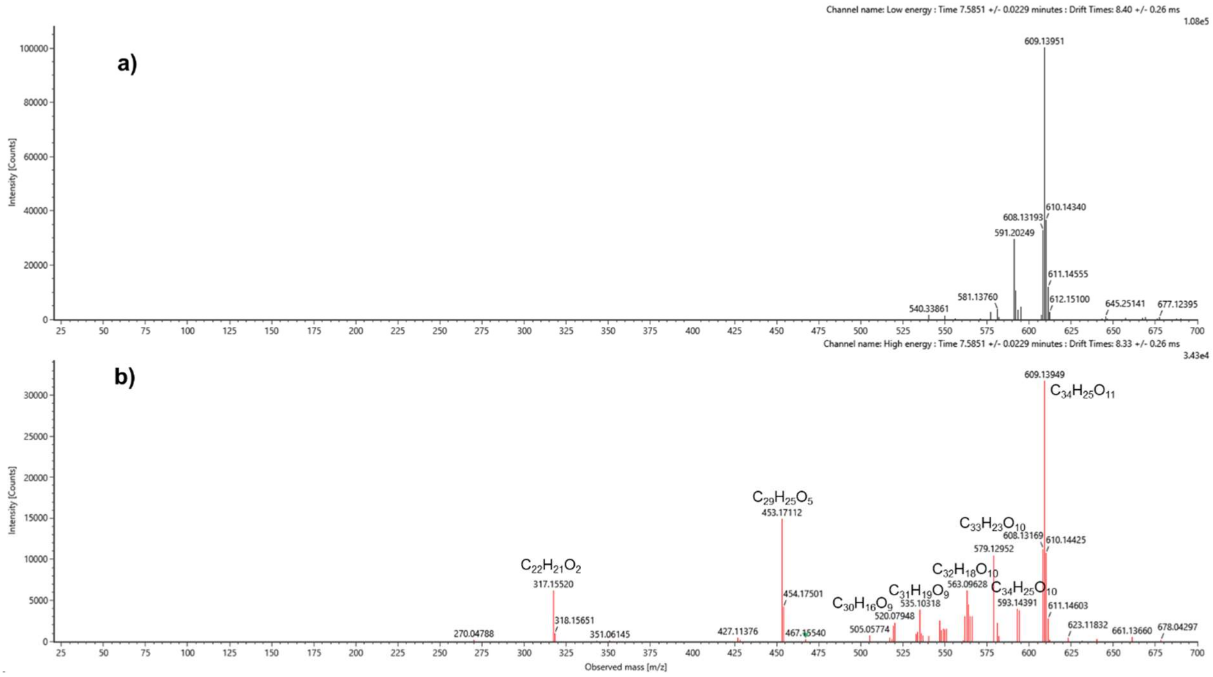

Table S1: Chromatographic conditions for compound isolation from *Lotus japonicus*. MPLC: medium pressure liquid chromatography; SPE: solid phase extraction; HPLC: high performance liquid chromatography.

Sample	System	Column	Flow rate [mL min^{-1}]	Solvent A	Solvent B	UV/VIS [nm]	Gradient
L. japonicus non-colonized root methanolic extract (30 g)	MPLC	LiChroprep RP18 (25-40 µm, Merck, Darmstadt, Germany)	40	0.1% formic acid in water	methanol	220	0% B for 8 min, 10% B for 8 min, 20% B for 8 min, 30% B for 8 min, 40% B for 8 min, 50% B for 8 min, 60% B for 8 min, 70% B for 8 min, 80% B for 8 min, 90% B for 8 min, 100% B for 10 min
L. japonicus colonized root methanolic extract (13 g)	SPE	Chromabond C18ec (Macherey-Nagel, Düren, Germany)	vacuum applied	water	methanol	/	preconditioned with MeOH (60 mL), 70% MeOH in water (v/v, 60 mL), 30% MeOH in water (v/v, 60 mL), and water (60 mL), eluted with 0% B (60 mL), 10% B (60 mL), 20% B (60 mL), 30% B (60 mL), 40% B (60 mL), 50% B (60 mL), 60% B (60 mL), 70% B (60 mL), 80% B (60 mL), 90% B (60 mL), 100% B (120 mL)
Fraction M9 (1000 mg)	preparative HPLC	VarioPrep VP Nucleodur C18 Pyramid column (250 × 21 mm, 5 µm; Macherey-Nagel)	20	0.1% formic acid in water	acetonitrile	210	From 50% B to 65% B within 12 min, to 100% B within 5 min, keep at 100% B for 4 min, to 50% B for 4 min, keep at 50% B for 3 min
Subfraction M9-7 (35 mg)	preparative HPLC	Phenomenex Luna 5u Phenyl-Hexyl column (250 × 21.2 mm, 5 µm, Phenomenex, Aschaffenburg, Germany)	20	0.1% formic acid in water	methanol	220	50% B for 2 min, to 60% B within 10 min, to 100% B within 5 min, keep at 100% B for 4 min, to 50% B within 4 min, keep at 50% B for 3 min
Subfraction M9-7-4 (5 mg)	semi-preparative HPLC	Phenomenex Luna PFP (2) column (250 × 10 mm, 5 µm)	4.5	0.1% formic acid in water	acetonitrile	220	40% B to 45% B within 15 min, to 100% B within 5 min, keep at 100% B for 5 min, to 40% B within 2 min, keep at 40% B for 3 min
Fraction M10 (750 mg)	preparative HPLC	VarioPrep VP Nucleodur C18 Pyramid column (250 × 21 mm, 5 µm; Macherey-Nagel)	20	0.1% formic acid in water	acetonitrile	210	50% B for 2 min, to 65% B within 10 min, to 100% B within 5 min, keep at 100% B for 4 min, to 50% B within 4 min, keep at 50% B for 3 min
Subfraction M10-10 (5 mg)	semi-preparative HPLC	Phenomenex Luna PFP (2) column (250 × 10 mm, 5 µm)	4.5	0.1% formic acid in water	methanol	220	80% B to 90% B within 2.5 min, to 100% B within 9.5 min, keep at 100% B for 8 min, to 80% B within 2 min, keep at 80% B for 3 min
Fraction S8 (470 mg)	preparative HPLC	Phenomenex Luna 5u Phenyl-Hexyl column (250 × 21.2 mm, 5 µm)	20	0.1% formic acid in water	methanol	210	80% B for 2 min, to 90% B within 8 min, to 100% B within 7 min, keep at 100% B for 4 min, to 80% within 4 min, 80% for 3 min
Subfraction S8-9 (47 mg)	semi-preparative HPLC	Phenomenex Luna PFP (2) column (250 × 10 mm, 5 µm)	4.5	0.1% formic acid in water	acetonitrile	220	50% B to 57.5% B within 4 min, keep at 57.5% B for 11 min, to 100% B within 4.5 min, keep at 100% B for 4 min, to 50% B within 3.5 min, keep at 50% B for 3 min
Subfraction S8-10 (25 mg)	semi-preparative HPLC	Phenomenex Luna PFP (2) column (250 × 10 mm, 5 µm)	4.5	0.1% formic acid in water	acetonitrile	220	80% B to 85% B within 7 min, keep at 85% B for 8 min, to 100% B within 4 min, keep at 100% B for 4 min, to 80% B within 3 min, keep at 80% B for 4 min
Subfraction S8-6 (35 mg)	semi-preparative HPLC	Phenomenex Luna PFP (2) column (250 × 10 mm, 5 µm)	4.5	0.1% formic acid in water	acetonitrile	220	50% B for 12 min, to 55% B within 3 min, to 100% B within 4.5 min, keep at 100% B for 4 min, to 50% B within 3.5 min, keep at 50% B for 3 min

Subfraction S8-11 (21 mg)	semi-preparative HPLC	Phenomenex Luna PFP (2) column (250 × 10 mm, 5 µm)	4.5	0.1% formic acid in water	acetonitrile	220	50% B to 60% B within 4 min, keep at 60% B for 11 min, to 100% B within 4.5 min, keep at 100% B for 4 min, to 50% B within 3.5 min, keep at 50% B for 3 min
Subfraction S8-14 (8 mg)	semi-preparative HPLC	Phenomenex Luna PFP (2) column (250 × 10 mm, 5 µm)	4.5	0.1% formic acid in water	acetonitrile	220	60% B, keep for 8 min, to 100% B within 8 min, keep at 100% B for 5 min, to 60% B within 2 min, keep at 60% for 4 min

Table S2: Colonization data including intraradical hyphae, arbuscules, vesicles, total root length colonization and mean colonization per genotype (cf. xlsx-file)

Table S3: Potential marker candidates in *Lotus japonicus* wild-type and mutant roots (cf. xlsx-file)

Table S4: Metabolites included in the in-house *in silico* database (cf. xlsx-file)

Table S5: *In silico* database hits for mass spectral features in 7 wpi root samples (cf. xlsx-file)

Table S6: *In silico* database hits for mass spectral features in 10 wpi root samples (cf. xlsx-file)

Table S7: ToF-MS^e and NMR data of isolated compounds. ToF-MS^e: Time-of-flight-Mass Spectrometry with elevated energy; NMR: nuclear magnetic resonance spectroscopy; CCS: collision cross-section; δ: chemical shift; *J*: coupling constant.

Ayamenin D (**14**)			
TOF-MS calculated (*m/z*)	313.0354	$[C_{16}H_9O_7]^-$	
TOF-MS measured (*m/z*)	313.0349		
MS^e fragments; high collision energy ramp −20 – −60 eV *m/z* (%)	313.03 (14), 298.01 (100), 269.01 (30), 242.02 (6)		
CCS $[M-H]^-$, in nitrogen [Å^2]	165.8		
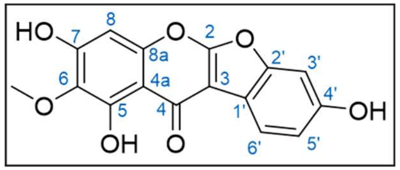			
^{1}H NMR	600 MHz, 300 K, CD_3CN		
^{13}C NMR	151 MHz, 300 K, CD_3CN		
**Position**	**δ_C [ppm]**	**δ_H [ppm] (m)**	***J* [Hz]**
2	165.0		
3	97.4		
4	179.2		
4a	104.1		
5	154.0		
6	131.7		
7	155.7		
8	94.5	6.66 (s)	
8a	150.3		
1′	114.4		
2′	150.4		
3′	98.7	7.09 (d)	2.1
4′	155.9		
5′	113.6	6.95 (dd)	2.1, 8.3
6′	121.5	7.79 (d)	8.3
6-OMe	60.1	3.90 (s)	

Lupinalbin A (**4**)			
TOF-MS calculated (*m/z*)	283.0248	$[C_{15}H_7O_6]^-$	
TOF-MS measured (*m/z*)	283.0245		
MS^e fragments; high collision energy ramp −20 – −60 eV *m/z* (%)	283.02 (100), 265.01 (8), 255.03 (41), 239.03 (31), 227.03 (14), 211.04 (7), 173.02 (35), 149.02 (7)		
CCS $[M-H]^-$, in nitrogen [Å^2]	155.8		
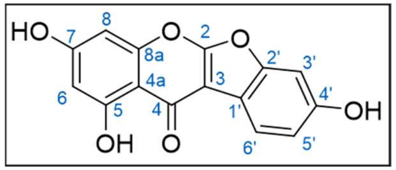			
^{1}H NMR	600 MHz, 300 K, CD_3CN		
^{13}C NMR	151 MHz, 300 K, CD_3CN		
**Position**	**δ_C [ppm]**	**δ_H [ppm] (m)**	***J* [Hz]**
2	165.0		
3	97.5		
4	178.8		
4a	103.5		
5	162.9		
6	99.9	6.35 (d)	2.2
7	163.9		
8	94.9	6.57 (d)	2.2
8a	155.3		
1′	114.6		
2′	150.4		
3′	98.8	7.12 (d)	2.2
4′	155.9		
5′	113.6	6.97 (dd)	2.2, 8.3
6′	121.4	7.81 (d)	8.3

5,7-Dihydroxy-4′-methoxycoumaronochromone (Lotuschromone, **13**)			
TOF-MS calculated (*m/z*)	297.0405	$[C_{16}H_9O_6]^-$	
TOF-MS measured (*m/z*)	297.0401		
MS^e fragments; high collision energy ramp −20 – −60 eV *m/z* (%)	297.04 (76), 282.02 (100), 269.05 (7), 254.02 (86), 238.03 (7), 226.03 (33), 198.03 (16), 182.04 (6), 172.02 (9)		
CCS $[M-H]^-$, in nitrogen [Å^2]	162.7		
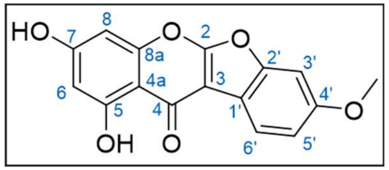			
^{1}H NMR	950 MHz, 300 K, CD_3CN		
^{13}C NMR	239 MHz, 300 K, CD_3CN		
**Position**	**δ_C [ppm]**	**δ_H [ppm] (m)**	***J* [Hz]**
2	165.1		
3	97.5		
4	178.9		
4a	103.4		
5	162.9		
6	100.0	6.36 (d)	2.1
7	164.0		
8	95.0	6.58 (d)	2.1
8a	155.3		
1′	115.3		
2′	150.4		
3′	97.2	7.28 (d)	2.2
4′	158.6		
5′	113.1	7.08 (dd)	2.2, 8.4
6′	121.3	7.89 (d)	8.4
4′-OMe	55.6	3.90 (s)	

4-Hydroxy-2-(2′-hydroxy-4′-methoxyphenyl)-6-methoxybenzofuran-3-carbaldehyde (Lotusaldehyde, **15**)		
TOF-MS calculated (*m/z*)	313.0718	$[C_{17}H_{13}O_6]^-$
TOF-MS measured (*m/z*)	313.0715	
MS^e fragments; high collision energy ramp −20 – −60 eV *m/z* (%)	313.07 (40), 298.05 (9), 285.08 (36), 270.05 (95), 255.03 (100), 227.03 (51), 183.04 (23)	
CCS $[M-H]^-$, in nitrogen [Å^2]	169.7	

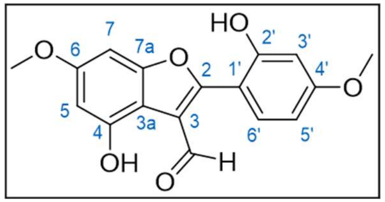

^1H NMR	600 MHz, 300 K, CD_3OD		
^{13}C NMR	151 MHz, 300 K, CD_3OD		
**Position**	**δ_C [ppm]**	**δ_H [ppm] (m)**	***J* [Hz]**
2	163.6		
3	117.7		
3a	106.8		
4	151.5		
5	97.7	6.35 (d)	1.6
6	161.0		
7	87.3	6.68 (d)	1.6
7a	156.6		
1′	108.4		
2′	156.9		
3′	101.3	6.59 (d)	1.9
4′	163.5		
5′	106.1	6.66 (dd)	1.9, 8.6
6′	132.0	7.55 (d)	8.6
3-CHO	190.3	9.95 (s)	
6-OMe	54.8	3.84 (s)	
4′-OMe	54.5	3.87 (s)	

7-Hydroxy-3,9-dimethoxypterocarp-6a-ene (Lotuscarpene, **16**)		
TOF-MS calculated (*m/z*)	297.0768	$[C_{17}H_{13}O_5]^-$
TOF-MS measured (*m/z*)	297.0763	
MS^e fragments; high collision energy ramp −20 – −60 eV *m/z* (%)	297.08 (20), 282.05 (46), 267.03 (100), 255.03 (22), 239.03 (33), 227.03 (12), 223.04 (9), 211.04 (22), 199.04 (10), 183.04 (10), 167.05 (8)	
CCS $[M-H]^-$, in nitrogen [Å^2]	169.2	

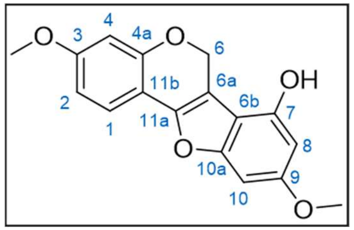

^1H NMR	600 MHz, 300 K, CD_3OD		
^{13}C NMR	151 MHz, 300 K, CD_3OD		
**Position**	**δ_C [ppm]**	**δ_H [ppm] (m)**	***J* [Hz]**
1	131.2	7.30 (d)	8.5
2	105.3	6.55 (dd)	2.2, 8.5
3	161.6		
4	101.1	6.51 (d)	2.2
4a	156.0		
6	56.4	4.75 (s)	
6a	114.8		
6b	111.5		
7	150.6		
8	96.7	6.29 (d)	1.9
9	159.2		
10	87.3	6.60 (d)	1.9
10a	156.7		
11a	146.2		
11b	110.4		
3-OMe	54.7	3.82 (s)	
9-OMe	54.3	3.81 (s)	

4,6-Dihydroxy-2-(2′-hydroxy-4′-methoxyphenyl)-3-(methoxymethyl)benzofuran (**25**)		
TOF-MS calculated (*m/z*)	315.0874	$[C_{17}H_{15}O_6]^-$
TOF-MS measured (*m/z*)	315.0872	
MS^e fragments; high collision energy ramp −20 – −60 eV *m/z* (%)	315.09 (19), 283.06 (24), 268.04 (100), 267.03 (36), 240.04 (25), 239.03 (16), 223.04 (5), 211.04 (13), 195.05 (8), 167.05 (4)	
CCS $[M-H]^-$, in nitrogen [Å^2]	174.3	

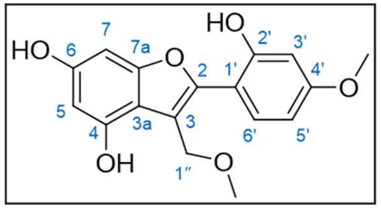

^1H NMR	600 MHz, 300 K, CD_3CN		
^{13}C NMR	151 MHz, 300 K, CD_3CN		
**Position**	**δ_C [ppm]**	**δ_H [ppm] (m)**	***J* [Hz]**
2	146.6		
3	112.7		
3a	110.2		
4	151.0		
5	97.8	6.24 (d)	1.9
6	156.6		
7	89.8	6.51 (d)	1.9
7a	156.7		
1′	109.7		
2′	156.0		
3′	101.8	6.58 (d)	2.4
4′	161.8		
5′	106.3	6.60 (dd)	2.4, 8.5
6′	131.7	7.26 (d)	8.5
1″	66.5	4.65 (s)	
4′-OMe	55.1	3.83 (s)	
1″-OMe	57.5	3.48 (s)	

4-Hydroxy-2-(2′-hydroxy-4′-methoxyphenyl)-6-methoxy-3-(methoxymethyl)benzofuran (**26**)			
TOF-MS calculated (*m/z*)	329.1031	$[C_{18}H_{17}O_6]^-$	
TOF-MS measured (*m/z*)	329.1029		
MS^e fragments; high collision energy ramp −20 – −60 eV *m/z* (%)	329.10 (20), 297.08 (22), 282.05 (56), 267.03 (100), 239.03 (30), 223.04 (7), 211.04 (14), 199.04 (6), 53.59 (8)		
CCS $[M-H]^-$, in nitrogen [Å^2]	179.6		
^{1}H NMR	600 MHz, 300 K, CD_3CN		
^{13}C NMR	151 MHz, 300 K, CD_3CN		
**Position**	**δ_C [ppm]**	**δ_H [ppm] (m)**	***J* [Hz]**
2	147.2		
3	112.5		
3a	110.9		
4	150.9		
5	97.2	6.34 (d)	1.9
6	159.5		
7	87.9	6.65 (d)	1.9
7a	156.7		
1′	109.8		
2′	156.1		
3′	101.7	6.56 (d)	2.3
4′	161.9		
5′	106.1	6.59 (dd)	2.3, 8.5
6′	131.7	7.29 (d)	8.5
1″	66.5	4.66 (s)	
6-OMe	55.4	3.81 (s)	
4′-OMe	55.1	3.82 (s)	
1″-OMe	57.5	3.47 (s)	

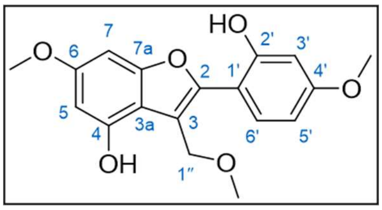

Dimeric polyphenol artefact (**29**)			
TOF-MS calculated (*m/z*)	597.1402	$[C_{33}H_{25}O_{11}]^-$	
TOF-MS measured (*m/z*)	597.1396		
MS^e fragments; high collision energy ramp −20 – −60 eV *m/z* (%)	597.14 (100), 579.13 (36), 565.12 (78), 547.17 (12), 521.13 (11), 497.13 (9), 479.11 (7), 441.06 (15), 315.09 (14), 283.06 (78)		
CCS $[M-H]^-$, in nitrogen [Å^2]	248.8		
^{1}H NMR	600 MHz, 300 K, $(CD_3)_2CO$		
^{13}C NMR	151 MHz, 300 K, $(CD_3)_2CO$		
**Position**	**δ_C [ppm]**	**δ_H [ppm] (m)**	***J* [Hz]**
2	147.5		
3	112.7		
3a	112.2		
4/6	153.7		
5/7	92.8	6.24 (s)	
4/6	159.6		
5/7	103.6		
7a	151.0		
1′	109.9		
2′	156.3		
3′	102.0	6.69 (d)	2.1
4′	162.0		
5′	106.1	6.70 (dd)	2.1, 8.2
6′	131.6	7.45 (d)	8.2
1″	66.5	4.68, 4.78 (s, s)	
4′-OMe	54.8	3.87 (s)	
1″-OMe	57.3	3.48 (s)	
j	130.4	8.34 (d)	8.7
h	109.3	6.76 (dd)	2.6, 8.7
m	160.6		
f	101.7	6.47 (d)	2.6
k	155.2		
a	69.1	4.56, 4.71 (s, s)	
d	95.8		
g	102.8		
l	156.4		
n	161.8		
o	162.2		
b	89.8	5.83 (d)	1.9
e	95.9	6.05 (d)	1.9
c	92.1		
i	116.4		
m-OMe	54.8	3.78 (s)	

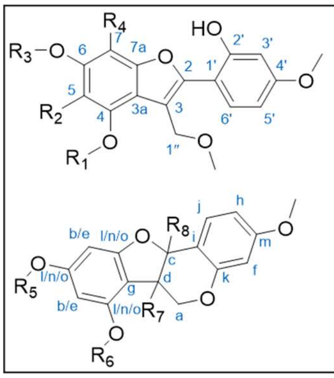

Table S8: Annotated transcriptome data of *Lotus japonicus* AM and control root samples (cf. xlsx-file)

Table S9: Significantly AM-induced polyphenol biosynthesis genes (cf. xlsx-file)

Methods S1: Marker compound isolation.

The methanol (MeOH) extract of non-colonized roots (30 g) was suspended in MeOH (240 mL) and fractionated by MPLC (Table S1) to collect eleven fractions (M1 to M11).

The MeOH extract of colonized roots (13 g) was suspended in water (1000 mL) and subjected to solid-phase extraction (SPE, Table S1) using Chromabond C18 ec silica cartridges (Macherey-Nagel, Düren, Germany) and a Chromabond SPE Vacuum Manifold (Macherey-Nagel) to collect eleven fractions (S1 to S11).

Isolation of Ayamenin D (14) and Lupinalbin A (4) from Fraction M9. An aliquot of M9 (1000 mg) was dissolved in MeOH (50 mL) and further fractionated using preparative HPLC (Table S1) to collect 14 fractions (M9-1 to M9-14). An aliquot of M9-7 (35 mg) was dissolved in MeOH (10 mL) and fractionated using preparative HPLC (Table S1) to obtain nine fractions (M9-7-1 to M9-7-9). Fraction M9-7-4 (5 mg) was dissolved in MeOH (2.5 mL) and further purified using semi-preparative HPLC (Table S1) which yielded nine fractions (M9-7-4-1 to M9-7-4-9). 5,7,4′-Trihydroxy-6-methoxycoumaronochromone (ayamenin D, **14**, 2.0 mg) was obtained from fraction M9-7-4-7 and fraction M9-7-4-8 contained 5,7,4′-trihydroxycoumaronochromone (lupinalbin A, **4**, 1.7 mg). The residues were used for structural analysis and as an analytical reference compound. 1/2D NMR and ToF-MS^e (See Table S7) experiments verified the structures (see Fig. 3), and the data were also in accordance to previously published results (Hanawa *et al.*, 1991a, 1991b; Ateba *et al.*, 2014).

Isolation of 5,7-Dihydroxy-4′-methoxycoumaronochromone (Lotuschromone, 13) from Fraction M10. An aliquot of M10 (750 mg) was dissolved in MeOH (100 mL) and fractionated using preparative HPLC (Table S1) to collect 14 fractions (M10-1 to M10-14). Fraction M10-10 (5 mg) was dissolved in MeOH (2.5 mL) and further purified using semi-preparative HPLC (Table S1) to collect twelve fractions (M10-10-1 to M10-10-12). 5,7-Dihydroxy-4′-methoxycoumaronochromone (**13**, 0.5 mg) was yielded in fraction M10-10-7. The residue was used for structural analysis and as authentic reference compound. 1/2D NMR and ToF-MS^e experiments (Table S7) verified the structure (see Fig. 3).

Separation of S8 by Preparative HPLC. Fraction S8 (470 mg) was dissolved in MeOH (50 mL) and further purified by preparative HPLC (Table S1) to collect 21 fractions (S8-1 to S8-21).

Isolation of 4-Hydroxy-2-(2′-hydroxy-4′-methoxyphenyl)-6-methoxybenzofuran-3-carbaldehyde (Lotusaldehyde, 15) from Fraction S8-9. An aliquot of S8-9 (47 mg) was dissolved in MeCN (20 mL) and fractionated by semi-preparative HPLC (Table S1) to collect 20 fractions (S8-9-1 to S8-9-20). In fraction S8-9-14, 4-hydroxy-2-(2′-hydroxy-4′-methoxyphenyl)-6-methoxybenzofuran-3-carbaldehyde (**15**, 2.7 mg) was yielded and used for

structural analysis and as a reference compound. 1/2D NMR and ToF-MS^e experiments (Table S7) verified the structure (see Fig. 3).

Isolation of 7-Hydroxy-3,9-dimethoxypterocarp-6a-ene (Lotuscarpene, 16) from Fraction S8-10. An aliquot of S8-10 (25 mg) was dissolved in MeCN (20 mL) and fractionated by semi-preparative HPLC (Table S1) to collect 14 fractions (S8-10-1 to S8-10-14). In fraction S8-10-4, 7-hydroxy-3,9-dimethoxypterocarp-6a-ene (**16**, 3.3 mg) was yielded and used for structural analysis and as an analytical standard compound. 1/2D NMR and ToF-MS^e experiments (Table S7) verified the structure (see Fig. 3).

Isolation of 4,6-Dihydroxy-2-(2′-hydroxy-4′-methoxyphenyl)-3-(methoxymethyl)benzofuran (25) from Fraction S8-6. Fraction S8-6 (35 mg) was dissolved in MeOH (20 mL) and purified by semi-preparative HPLC (Table S1) to collect 15 fractions (S8-6-1 to S8-6-15). 4,6-Dihydroxy-2-(2′-hydroxy-4′-methoxyphenyl)-3-(methoxymethyl)benzofuran (**25**, 1.5 mg) was yielded in fraction S8-6-10 and used for structural analysis. 1/2D NMR and ToF-MS^e experiments (Table S7) verified the structure (see Fig. 3).

Isolation of 4-Hydroxy-2-(2′-hydroxy-4′-methoxyphenyl)-6-methoxy-3-(methoxymethyl)benzofuran (26) from Fraction S8-11. Fraction S8-11 (21 mg) was dissolved in MeOH (20 mL) and purified by semi-preparative HPLC (Table S1) to collect 15 fractions (S8-11-1 to S8-11-15). 4-Hydroxy-2-(2′-hydroxy-4′-methoxyphenyl)-6-methoxy-3-(methoxymethyl)benzofuran (**26**, 2.0 mg) was yielded in fraction S8-11-11 and used for structural analysis. 1/2D NMR and ToF-MS^e experiments (Table S7) verified the structure (see Fig. 3).

Isolation of a Dimeric Polyphenol Artefact (29) from Fraction S8-14. Subfraction S8-14 (8 mg) was dissolved in MeCN (5 mL) and purified by semi-preparative HPLC (Table S1) to collect 3 fractions (S8-14-1 to S8-14-3). From fraction S8-14-2, an unknown dimeric polyphenol artefact (**29**, 2.0 mg) was obtained, and 1/2D NMR and ToF-MS^e experiments (Table S7) were performed.

Methods S2: Omics correlation analysis

The transcriptomics data set consists of 30586 genes across eight samples (four AM and four control samples). Using KEGG (Kyoto Encyclopedia of Genes and Genomes, Kanehisa & Goto, 2000; Kanehisa, 2019; Kanehisa *et al.*, 2023) database, the data were screened for *Arabidopsis thaliana* (AT) IDs from ortholog tables of phenylpropanoid (map00940), flavonoid (map00941), anthocyanin (map00942), isoflavonoid (map00943), flavone and flavonol (map00944), and stilbenoid, diarylheptanoid and gingerol (map00945) biosynthesis. For 149

unique AT IDs, 242 *L. japonicus* genes were linked. After applying significance (*p*-value <0.05) and fold change (>1.5) thresholds, 19 *L. japonicus* genes were obtained. Heatmap visualization of transcriptomics data, metabolomics data, and fold change comparison was performed using R package gplot (version 3.1.3) with row wise scaling. Metabolomics *p*-values and fold change was obtained from Progenesis analysis of UPLC-ToF-MS data.

Notes S1: Analysis of NMR Spectra of 5,7-dihydroxy-4′-methoxycoumaronochromone (lotuschromone, **13**)

The ^{1}H NMR signals of **13** were very similar to the ones of **4** with four doublets, one doublet of doublets, and one additional signal of a methyl group singlet at δ_H 3.90 ppm (Fig. S8). The ^{13}C, ^{1}H-^{1}H-COSY, ^{1}H-^{13}C-HSQC, and ^{1}H-^{13}C-HMBC NMR spectra (Fig. S8) reveal one carbonyl carbon (δ_C 178.9 ppm), six quaternary carbons located directly next to an oxygen atom (δ_C 150.4–165.1 ppm), five aryl-bound methine carbons and three further quaternary carbon atoms (δ_C 95.0–121.3 ppm), and one methyl carbon (δ_C 55.6 ppm). The signals confirm the identical carbon skeleton of **13** and **4**. Carbon atoms C(4a) (δ_C 103.4 ppm) and C(7) (δ_C 164.0 ppm) lacking signals in the ^{13}C spectrum were assigned according to HMBC correlations with H-C(6) (δ_H 6.4 ppm) and H-C(8) (δ_H 6.6 ppm) (see Fig. S8, Table S7). The correlation of MeO-C(4′) (δ_H 3.9 ppm) with C(4′) (δ_C 158.6 ppm) highlights the position of the methoxy group. Fig. S8**f** provides a summary of other significant HMBC correlations, and the full assignment of ^{1}H and ^{13}C NMR signals is available in Table S7. **13** was identified as 5,7-dihydroxy-4′-methoxycoumaronochromone.

Notes S2: Analysis of NMR Spectra of 4-hydroxy-2-(2′-hydroxy-4′-methoxyphenyl)-6-methoxybenzofuran-3-carbaldehyde (lotusaldehyde, **15**)

In the ^{1}H and ^{1}H-^{1}H-COSY NMR spectra, two methyl group singlets (δ_H 3.8 and 3.9 ppm), two doublets (δ_H 6.4 and 6.7 ppm; $^{4}J_{H,H}$ = 1.6 Hz) of vicinal protons and an AMX spin system (δ_H 6.6, 6.7, and 7.6 ppm; $^{3}J_{H,H}$ = 8.6 Hz and $^{4}J_{H,H}$ = 1.9 Hz) implying two separate aromatic ring systems were detected (Fig. S9). One exposed singlet in the low field at δ_H 10.0 ppm indicates an aldehyde proton. The double bond equivalent (DBE) number for $C_{17}H_{14}O_6$ equals 11, so, including two aromatic rings (2 × DBE +4) and one aldehyde group (DBE +1), two molecular features contributing to the DBE number are missing. Those features can be rings and double bonds. The ^{13}C, ^{1}H-^{13}C-HSQC, and ^{1}H-^{13}C-HMBC NMR spectra show the aldehyde carbon (δ_C 190.3 ppm), six quaternary carbon atoms bound to an oxygen atom (δ_C 151.5–163.6 ppm), five aryl methine carbon atoms and three further quaternary carbons (δ_C 87.3–132.0 ppm), as well as two methyl carbons (δ_C 54.5–54.8 ppm). These signals confirm the two aromatic ring systems leaving two quaternary carbon atoms to complete the two missing DBE features. This is enabled by one five-membered furan ring annealed to one of the aromatic rings (DBE +2) to form a benzofuran moiety.

The carbon at δ_C 106.8 ppm shows HMBC correlations to the vicinal protons (δ_H 6.4 and 6.7 ppm) and the aldehyde proton (δ_H 10.0 ppm) revealing it is part of the aromatic ring (C(3a)) of the benzofuran moiety and the aldehyde function is bound at position 3 of the furan ring (^{3}J-

coupling to C(3a)). The vicinal protons of the benzofuran ring are located at position 5 and 7, as both show intense HMBC signals indicating two times a ^3J-coupling to C(3a). One methoxy function is located at position 6 (δ_C 161.0 ppm) of the benzofuran ring, as there are HMBC correlations of H-C(5) and H-C(7) to C(6) as well. The missing two carbon signals of the aromatic ring are both directly linked to an oxygen atom with the carbon at δ_C 156.6 ppm showing a correlation to H-C(7) but lacking a correlation to H-C(5). This indicates its position at C(7a) connecting the benzene and the furan ring. Therefore, C(4) (δ_C 151.5 ppm) is left with a hydroxy function. The second aromatic ring is linked to the benzofuran at position two. For the AMX spin system, there are three possible combinations with the protons being bound at positions 3′, 4′, and 6′; at positions 3′, 5′, and 6′; or at position 2′, 5′, and 6′. The first combination can be ruled out as the proton with a dd-multiplet (δ_H 6.7 ppm) shows an intense HMBC ^3J-correlation to C(1′), which is linked to the benzofuran C(2). The carbon atoms which are linked to the protons at δ_H 6.6 ppm and δ_H 6.7 ppm and C(1′) are shifted to rather high field at δ_C 101.3, 106.1, and 108.4 ppm due to a two-fold shielding +M-effect of the hydroxy and methoxy function in the ring. Therefore, the AMX spin system protons are located at C(3′), C(5′), and C(6′). The methoxy function is linked to C(4′), as C(4′) also shows HMBC correlations to all AMX protons. Position C(2′) with the hydroxy group attached does not show an HMBC correlation to H-C(5′).

Further, the signals of **15** were well in line with the ones of the corresponding 2′-methoxy-4′-hydroxy- and 2′,4′-dihydroxy-derivates Sesbagrandiflorain A and B (Noviany *et al.*, 2021). Fig. S9**g** provides a summary of all significant HMBC correlations, and the full assignment of ^{1}H and ^{13}C NMR signals is available in Table S7. **15** was identified as 4-hydroxy-2-(2′-hydroxy-4′-methoxyphenyl)-6-methoxybenzofuran-3-carbaldehyde.

Notes S3: Analysis of NMR Spectra of 7-hydroxy-3,9-dimethoxypterocarp-6a-ene (lotuscarpene, **16**)

The ^{1}H and ^{1}H-^{1}H-COSY NMR spectra (Fig. S10**a** and **c**) showed signals of the two methoxy group singlets (both at δ_H 3.8 ppm), one methylene singlet (δ_H 4.8 ppm), two doublets (δ_H 6.3 and 6.6 ppm; $^4J_{H,H}$ = 1.9 Hz) of vicinal protons, and one AMX spin system (δ_H 6.5, 6.6, and 7.3 ppm; $^3J_{H,H}$ = 8.5 Hz and $^4J_{H,H}$ = 2.2 Hz) of two isolated aromatic ring systems. Based on the DBE number of 11 for $C_{17}H_{14}O_5$, subtracting two aromatic rings (2 × DBE +4), three DBE relevant molecular features are left. The ^{13}C, ^{1}H-^{13}C-HSQC, and ^{1}H-^{13}C-HMBC NMR spectra reveal, besides the two methoxy group signals (δ_C 54.3 and 54.7 ppm) and one oxygen-bound methylene signal (δ_C 56.4 ppm), six quaternary carbon atoms linked to an oxygen atom (δ_C 146.2–161.6 ppm), the five aryl methine carbon atoms of the two aromatic spin systems, and

three further quaternary carbon atoms (δ_C 87.3–131.2 ppm). By assigning two carbon atoms to the methoxy groups and twelve carbon atoms to the two aromatic rings, three carbon atoms are left to fulfill the DBE number of 11. This can be accomplished by two additional heterocyclic ring systems and one double bond resulting in a tetracyclic molecule. Contrary to the tetracyclic coumaronochromones **4**, **13**, and **14**, there was no carbonyl signal in the ^{13}C spectrum (Fig. S10**b**).

The HMBC spectrum (Fig. S10**e**) permitts assignment of one methoxy group (δ_C 54.7 ppm) at C(3) (δ_C 161.6 ppm) to complete the aromatic A-ring with the AMX spin system by correlations of H-C(1) and H-C(2) (δ_H 6.6 and 7.3 ppm) to C(3). H-C(1) and the methylene group H-C(6) (δ_H 4.8 ppm) both exhibit correlation signals with oxygen-linked C(11a) (δ_C 146.2 ppm) and H-C(6) further correlates with C(6a) (δ_C 114.8 ppm) and C(6b) (δ_C 111.5 ppm) of the second aromatic ring fixing the core structure of a pterocarp-6a-ene with a double bond between C(6a) and C(11a). The substitution pattern of the second aromatic ring shows a methoxy group (δ_C 54.3 ppm) (δ_C 159.2 ppm), the two meta-coupling doublet protons at C(8) (δ_C 96.7 ppm) and C(10) (δ_C 87.3 ppm), and one hydroxy group at C(7) (δ_C 150.6 ppm). The signals obtained, especially the ones of the aromatic B ring, were in good accordance with ^{1}H and ^{13}C data from the structurally related aracarpene 2 (3,4,7-trihydroxy-9-methoxypterocarp-6a-ene) (Sobolev *et al.*, 2010). Accordingly, **16** was identified as 7-hydroxy-3,9-dimethoxypterocarp-6a-ene.

Notes S4: Analysis of NMR Spectra of the dimeric polyphenol artefact **29**

MS^e revealed a sum formula of $C_{33}H_{26}O_{11}$ and 21 DBE. The first part of the molecule was elucidated by comparing the signals with the ones of the methanol artefact **25** (Table S7). Signals highlighted an aromatic AMX spin system (δ_H 6.6, 6.7, and 7.5 ppm; $^3J_{H,H}$ = 8.2 Hz and $^4J_{H,H}$ = 2.0 Hz), a methoxymethyl group with a diastereotopic methylene moiety (δ_H 4.7 and 4.8 ppm; $^2J_{H,H}$ = 12.9 Hz), and one benzofuran ring that is linked to the second part of the molecule (Fig. S13, Table S7). This discloses the first part as a 2-(hydroxymethoxyphenyl)-3-(methoxymethyl)benzofuran. The benzofuran ring is characterized by a proton giving a singlet signal (δ_H 6.2 ppm) proving that the second part is linked somewhere at the aromatic ring.

For the second part, one AMX spin system was detected (δ_H 6.5, 6.8, and 8.3 ppm; $^3J_{H,H}$ = 8.7 Hz and $^4J_{H,H}$ = 2.6 Hz) along with another spin system of two *meta*-coupling protons (δ_H 5.8 and 6.1 ppm; $^4J_{H,H}$ = 1.9 Hz) giving a total of four aromatic rings in the compound. Further, another diastereotopic methylene group was found (δ_H 4.6 and 4.7 ppm; $^2J_{H,H}$ = 11.9 Hz), indicating neighboring chirality. HMBC cross correlations revealed the location of three methoxy functions, which are the methoxymethyl group and one at each AMX-ring in *ortho*-position to the X-proton. Chemical shifts showed that the other two aromatic rings both

bear three oxygen atoms in *meta*-position to each other. HMBC correlation of the methylene protons with carbon atoms of both aromatic rings (δ_C 102.8 and 155.2 ppm) and two further quarternary carbons (δ_C 92.1 and 95.8 ppm, labeled as C(c) and C(d)) indicate a tetracyclic structure of the second moiety in the shape of a pterocarpan or a pterocarpene. There are no HMBC signals that disclose how the two moieties are linked to each other as all protons that could be considered for cross correlations are in ^4J or ^5J coupling range to the quarternary carbon-atoms in question. To fulfill the DBE rule (DBE 21), one double bond or ring is remaining in the molecule. A double bond between C(c) and C(d) in conjugation to the methylene group would significantly de-shield the oxygen-bound carbon atom of the pterocarpene-structure which would result in a signal at roughly 150 ppm. Therefore, the actual chemical shifts of C(c) and C(d) propose that the two parts are linked at two positions via a five-membered ring comprising C(c), C(d), C(5) or C(7), and C(4) or C(6) including the attached oxygen atom of the benzofuran moiety of the first molecular part. Whether the molecule is linked via C(5) and C(4), C(5) and C(6), or C(7) and C(6) cannot be clarified (Fig. S13 and S14). The chemical shifts of C(c) and C(d) imply that both are connected to an oxygen atom which means that C(d) is linked to C(4) or C(6) via an ether bridge. Similar polyphenolic ether bridges have been described before in the six-membered rings of procyanidin A_1 and A_2 (Schilling *et al.*, 1973). Further HMBC experiments varying in different coupling constants as well as ROESY did not gain more insights, therefore, the 3 structure predictions depicted in Fig. S14 were suggested.

References

Ateba SB, Njamen D, Medjakovic S, Zehl M, Kaehlig H, Jungbauer A, Krenn L. 2014. Lupinalbin A as the most potent estrogen receptor α- and aryl hydrocarbon receptor agonist in *Eriosema laurentii* de Wild. (Leguminosae). *BMC complementary and alternative medicine* **14**: 294.

Hanawa F, Tahara S, Mizutani J. 1991a. Isoflavonoids produced by *Iris pseudacorus* leaves treated with cupric chloride. *Phytochemistry* **30**: 157–163.

Hanawa F, Tahara S, Mizutani J. 1991b. Retro-Diels-Alder fragmentation of coumaronochromones and complete assignments of ^{13}C NMR data for lupinalbin A and ayamenin B. *Heterocycles* **32**: 1563.

Kanehisa M. 2019. Toward understanding the origin and evolution of cellular organisms. *Protein science : a publication of the Protein Society* **28**: 1947–1951.

Kanehisa M, Furumichi M, Sato Y, Kawashima M, Ishiguro-Watanabe M. 2023. KEGG for taxonomy-based analysis of pathways and genomes. *Nucleic acids research* **51**: D587-D592.

Kanehisa M, Goto S. 2000. KEGG: Kyoto encyclopedia of genes and genomes. *Nucleic acids research* **28**: 27–30.

Noviany N, Samadi A, Carpenter EL, Abugrain ME, Hadi S, Purwitasari N, Indra G, Indra A, Mahmud T. 2021. Structural revision of sesbagrandiflorains A and B, and synthesis and biological evaluation of 6-methoxy-2-arylbenzofuran derivatives. *Journal of natural medicines* **75**: 66–75.

Schilling G, Weinges K, Müller O, Mayer W. 1973. ^{13}C-NMR-Spektroskopische Konstitutionsermittlung der $C_{30}H_{24}O_{12}$-Procyanidine. *Justus Liebigs Annalen der Chemie* **1973**: 1471–1475.

Sobolev VS, Neff SA, Gloer JB, Khan SI, Tabanca N, Lucca AJ de, **Wedge DE. 2010.** Pterocarpenes elicited by *Aspergillus caelatus* in peanut (*Arachis hypogaea*) seeds. *Phytochemistry* **71**: 2099–2107.
